# Green synthesis of 1,3,5-triazine derivatives using a sonochemical protocol

**DOI:** 10.1016/j.ultsonch.2024.106951

**Published:** 2024-06-12

**Authors:** Damian Kułaga, Anna K. Drabczyk, Przemysław Zaręba, Jolanta Jaśkowska, Julia Chrzan, Katarzyna Ewa Greber, Krzesimir Ciura, Damian Plażuk, Ewelina Wielgus

**Affiliations:** a Department of Organic Chemistry and Technology, Faculty of Chemical Engineering and Technology, Cracow University of Technology, 24 Warszawska Street, 31-155 Cracow, Poland; b Department of Chemical Technology and Environmental Analytics, Faculty of Chemical Engineering and Technology, Cracow University of Technology, 24 Warszawska Street, 31-155 Cracow, Poland; c Department of Physical Chemistry, Faculty of Pharmacy, Medical University of Gdansk, Aleja Generała Józefa Hallera 107, 80-416 Gdansk, Poland; d Laboratory of Environmental Chemoinformatics, Faculty of Chemistry, University of Gdansk, Wita Stwosza 63, 80-308 Gdansk, Poland; e University of Lodz, Faculty of Chemistry, Department of Organic Chemistry, Laboratory of Molecular Spectroscopy, 12 Tamka Street, 91-403 Łódź, Poland; f Centre of Molecular and Macromolecular Studies, Polish Academy of Science,112 Sienkiewicza Street, 90-363 Łódź, Poland

**Keywords:** Sonochemistry, 1,3,5-triazines, Green chemistry, Eco-friendly

## Abstract

1,3,5-triazine derivatives are useful compounds with potential applications in various branches of chemical industry, including pharmaceutical chemistry, cosmetic chemistry, photochemistry, and organic chemistry. Due to the growing environmental requirements on conducting efficient, economical, and safe syntheses, development of new methods for synthesizing organic compounds is highly desirable. In this publication, we present a protocol for the synthesis of 1,3,5-triazine derivatives using a sonochemical approach. In as little as 5 min, it is possible to obtain most of the investigated compounds with a yield of over 75%. An undeniable advantage of this method, besides its short time, is the use of water as the solvent. Furthermore, we provide examples that the sonochemical method may be more versatile than the competing microwave method. Analysis conducted using the DOZN^TM^ 2.0 tool revealed that in terms of the 12 principles of green chemistry, the developed sonochemical method is 13 times “greener” than the classical one. Additionally, it has been demonstrated that the investigated molecules are attractive for their application as drug-like compounds.

## Highlights

• Triazine derivatives can be obtained with more than 70% of yield within 5 mins.
• Sonochemical approach requires water as solvent.
• Sonochemical protocol is 13 times “greener” than conventional heating.
• Compounds exhibit high lipophilicity, which may enable them to pass through BBB.

## Graphical abstract

**Figure:**
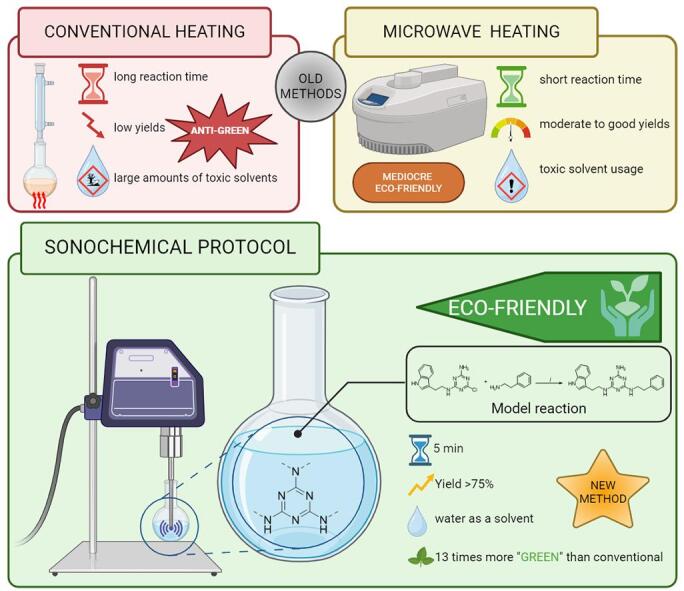

## 1 Introduction

**Scheme 1:**
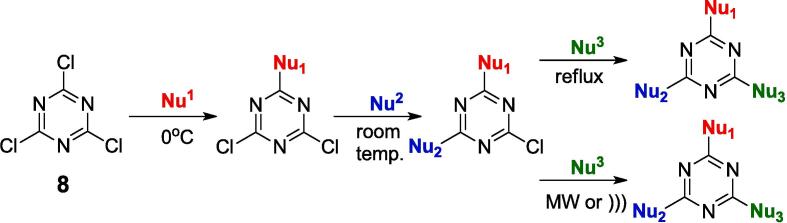
General approach of the 1,3,5-triazine core synthesis *via* a 3-step alkylation reaction. Nu – nucleophile. The third step may be conducted *vi*a the classic approach and the microwave (MW) or sonochemistry (*)))*) approach.

The 1,3,5-triazine core is widely encountered in organic chemistry [1], medicinal chemistry [2], photochemistry [3] and even cosmetics.[4] It appears as one of the leading motifs in many compounds with potential biological activity in anti-cancer [5], antibacterial [6], antiviral [7], anti-inflammatory [8], and antimalarial [9] agents, etc. Recent papers indicate that 1,3,5-triazine derivatives could be utilized for treating central nervous system (CNS) diseases as 5-HT_6_ or 5-HT_7_ receptor antagonists. [10], [11] The most common starting material for the synthesis of a range of compounds with the 1,3,5-triazine core is cyanuric chloride. Its involvement in synthesis is predictable and allows the gradual nucleophilic substitution of successive chlorine atoms by controlling the reaction temperature (Scheme 1). [12] The initial chlorine substitution typically occurs at 0 °C. Substitution of the second chlorine atom occurs at room temperature, while the third chlorine atom is substituted at higher temperatures, often at the boiling point of the solvent. This happens because the chlorine atoms in TCT act as strong electron acceptors, thereby facilitating the nucleophile attack. The substituted nucleophile introduces additional electrons into the triazine ring, making the ring increasingly electron-rich, which in turn makes it more difficult to introduce subsequent nucleophiles into the system. The electrons from the introduced nucleophile can also participate in the delocalization of the ring's electrons, making it less reactive to further substitution. Therefore, one of the ways to facilitate the substitution of the chlorine atom is to gradually increase the temperature, which helps the reaction to proceed.[33] This general approach facilitates easy and efficient production of a diverse range of compounds containing the 1,3,5-triazine core.

In most cases, the described third step of the reaction involves classical heating.[10] Unfortunately, this stage is the most energy-consuming, time-consuming, and consequently costly. Furthermore, the substantial resource consumption (energy and water for cooling) renders this methodology unfriendly to the environment. While this effect might be less noticeable on a small laboratory scale, scaling up the processes significantly increases reaction cost. Conventional heating, although technically straightforward, is not entirely efficient. This is due to heat transfer through the reactor walls before penetrating into the depth of the reaction mixture.[13] Additionally, the delivered heat undergoes partial dissipation.[13] Another problematic issue is the type of solvents used. In most cases, commonly used solvents include *N*,*N*-dimethylformamide (DMF), acetonitrile (ACN), DMSO (dimethyl sulfoxide), 1,4-dioxane or tetrahydrofuran (THF). These media are expensive, environmentally hazardous, and require proper disposal after the reaction.[14] The past Covid-19 pandemic revealed how fragile the supply chain of *Active Pharmaceutical Ingredients* (APIs) is from Asian (where the majority of known APIs are produced) to European countries, thereby disrupting drug production. To prevent such issues in the future, many organizations emphasize the need for increasing API production in the EU countries. However, production should be sustainable and adhere to stringent manufacturing rules, including environmental standards.[15] These issues indicate that the synthetic method for compounds including the 1,3,5-triazine core does not align with the principles of green chemistry, necessitating the exploration of new synthesis methods, which is fully justified.

**Scheme 2:**
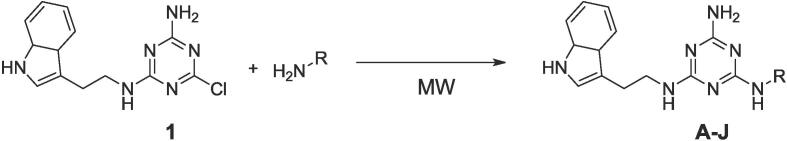
Microwave synthesis of known 1,3,5-triazines with the indole motif. MW − microwave synthesis.

Our previous research has shown that reactions under microwave irradiation can be carried out with higher yield, in a shorter period and be safer to the environment.[16], [17] This procedure, known as the *solvent-free* approach, usually uses TBAB (tetrabutylammonium bromide) as a phase transfer catalyst (PTC) and a catalytical amount of DMF, ACN or water serving an additional role as an energy transfer medium only. According to this procedure, olanzapine [18], [19], trazodone [20] or aripiprazole [16] may be obtained with a yield of more than 90 % over a few minutes. Aminotriazine derivatives with an indole motif [17] may be obtained in a shorter reaction time of up to 150 s. (Scheme 2, Table 1, reaction A vs. reactions B-D) with higher yields of up to 88 %. Thanks to microwave irradiation it was possible to reduce the excess of amine from 5 eq. (reaction A) to 3 eq. (reaction B).

**Table 1:** Classical and microwave synthesis of known 1,3,5-triazines with the indole motif (5-HT_7_ antagonists). % yield determined as the quantity of the isolated product after purification.

No.	R	Base	PTC	Solvent	Conditions	Time	Yield [%]	Ref.
A	CH_3_	DIPEA	−	THF	reflux	24 hrs.	42	17
B	CH_3_	K_2_CO_3_	TBAB	DMF	MW	150 s	86	17
C	CH_3_	K_2_CO_3_	TBAB	ACN	MW	150 s	75	17
D	CH_3_	K_2_CO_3_	TBAB	water	MW	150 s	37	17
E		K_2_CO_3_	TBAB	DMF	MW	150 s	88	17
F		K_2_CO_3_	TBAB	DMF	MW	150 s	59	21
G		K_2_CO_3_	TBAB	DMF	MW	150 s	59	21
H		K_2_CO_3_	TBAB	DMF	MW	150 s	43	21
I		K_2_CO_3_	TBAB	DMF	MW	150 s	78	21
J		K_2_CO_3_	TBAB	DMF	MW	150 s	82	21

Our recent studies have also shown that it is possible to use the sonochemistry approach to obtain olanzapine [18], quetiapine [18] or other long-chain arylpiperazines [23] as well as arylguanidines.[24] In most cases, the reactions were carried out in DMF, ACN, NaDES (Natural Deep Eutectic Solvents) or water in the presence of various bases including potassium carbonate, sodium hydroxide or DIPEA. Over 10 min to several hours, it was possible to obtain these compounds with a yield of 6 to 93 %.[18] The sonochemical approach can be used not only for conducting *N*-alkylation reactions but also for the synthesis of many other compounds. This method allows for the efficient formation of C-C bonds, for example, in the Blais or Michael condensation reactions, as well as the formation of C-Sn bond. Furthermore, reactions can be successfully carried out in the presence of ultrasound to oxidize alcohols or ketones to carboxylic acids.[34], [35].

**Scheme 3:**
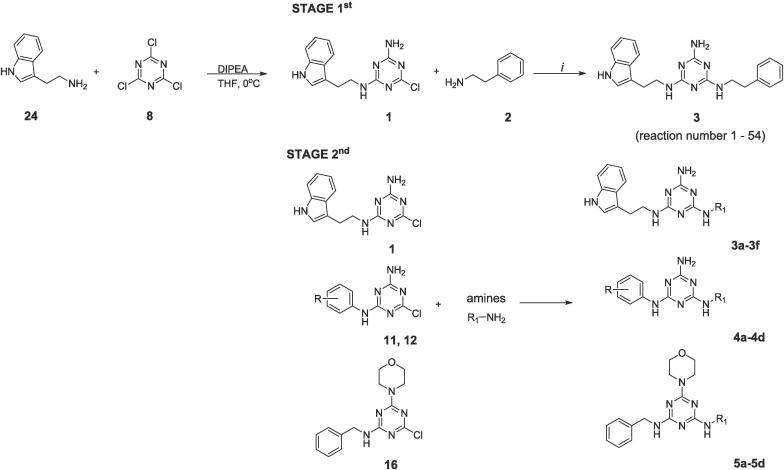
Selected model reaction for the 1st stage of research with selection of the principal cores used for synthesis in the 2nd step. amines – substituted anilines, arylpiperazines, phenylethylamines; R = H, m-Cl; i – various: bases, PTCs, solvents,))) source, reaction time. Synthesis of 1 was reported in ref. no [17].

Because of the high potency of compounds with the 1,3,5-triazine core, especially those with the indole motif that can be used in potential CNS drugs and the fact that these compounds have never been obtained through the sonochemical approach, herein we decided to develop an efficient and environmentally friendly synthesis method for 1,3,5-triazines *via* the *N*-alkylation reaction in the third step. The research was conducted in two stages: the first stage involved selecting the best synthetic conditions for the model reaction presented in Scheme 3. The second stage was assessing the utility of the developed method by synthesizing a library of compounds containing tryptamine, aniline or benzylamine scaffolds. Due to the potential application of 1,3,5-triazine derivatives as biologically useful compounds, it is essential to filter out compounds with excessively low or high values of certain parameters, such as lipophilicity or affinity for plasma proteins, at the early stages of the discovery phase of drug design. Compounds that do not meet the appropriate criteria for these parameters, despite having good pharmacological activity, will not have the chance to reach the intended site of action, such as the central nervous system. Thus selected physicochemical properties have been analyzed using a chromatographic approach to examine their lipophilicity, drug-plasma protein binding, and phospholipid affinity for further evaluation.

## 2 Material and methods

All primary substrates were purchased commercially from various sources. The solvents used for column chromatography and thin layer chromatography (TLC) had purity above 99.5 % and were purchased from Chempur. Cyanuric chloride, amines, PTCs and organic bases were purchased from Acros, Ambeed and Merck. Inorganic bases were purchased from Avantor. Solvents for LC-MS were purchased from Thermo Scientific. ^1^H and ^13^C NMR spectra were recorded using Bruker 400 MHz systems with TMS as an internal standard. Melting points were determined with the Böetius apparatus. Analytical thin-layer chromatography (TLC) was performed using 0.2 mm silica gel precoated aluminum sheets (60 F254, Merck) and UV light at 254 nm was used for visualization. Preparative thin-layer chromatography (pTLC) was performed using 2,000 µm silica gel precoated glass backed plates (F254, Silicycle). A VEVOR ultrasonic bath at 60 W power was used for the synthesis of compound **3** (reaction numbers: 1–50 and 52) over 60 min (reaction 52 over 20 min). A Sonicator VCX 130 PB ultrasonic reactor at 5–6 W power was used for: compounds **3** (reaction numbers: 53 and 54), **3a**-**3f**, **4a**-**4d**, **5a**-**5d** to obtain final compounds.

### 2.1 LC-MS, method a

HPLC-MS analyses were performed on a Shimadzu Nexera XR system equipped with PDA (SPD-M40) and LCMS-2020 detectors. Analyses were performed on a Phenomenex XB-C_18_ 1.7 µm (50 x 2.1 mm) (method 1A) column with a gradient of solvents as the mobile phase: Solvent A (0.01 % HCOOH in water) and B (0.01 % HCOOH in methanol); t = 0 min, 10 % of B, t = 4 min, 90 % of B, t = 6 min, 90 % of B, t = 6.1 min 10 % of B, stop time 11 min or a Phenomenex C_18_ 1.7 µm (50 x 2.1 mm) (method 2A) column with a gradient of solvents as the mobile phase: solvent A (0.01 % HCOOH in water) and solvent B (0.01 % HCOOH in MeOH): t = 0 min 5 % of B, t = 3 min 90 % of B, t = 4 min 90 % of B, t = 4.5 min 5 % of B stop time 7 min; flow rate 0.4 mL min-1; UV–VIS detection was performed in a range of 240–700 nm, MS data were collected in the ESI + mode in an *m*/*z* range of 100–800 with a scan speed of 15,000 u/s and an event time of 0.1 s.

### 2.2 LC-MS, method B

UPLC-MS/MS system: Waters Acquity Premier (Waters Corporation, Milford, MA, USA) coupled to a Waters Xevo TQ-S Cronos mass spectrometer (electrospray ionization mode: ESI). Chromatographic separations were carried out using an Acquity UPLC BEH (bridged ethylene hybrid) C_18_ column; 2.1 × 100 mm, and 1.7 µm particle size, equipped with an Acquity UPLC BEH C18 VanGuard pre-column; 2.1 × 5 mm, and 1.7 µm particle size. The column was maintained at 40 °C and eluted under gradient conditions using 95 % to 0 % of eluent A over 10 min, afterwards 100 % of eluent B over 3 min, at a flow rate of 0.3 mL min-1. Eluent A: water/formic acid (0.1 %, v/v); eluent B: acetonitrile/formic acid (0.1 %, v/v). Chromatograms were recorded using a Waters eλ PDA detector. Spectra were analyzed in the 200–700 nm range with 1.2 nm resolution and a sampling rate of 20 points/s. MS detection settings of the Waters Xevo TQ-S Cronos mass spectrometer were as follows: source temperature 150 °C, desolvation temperature 350 °C, desolvation gas flow rate 600 L/h, cone gas flow 100 L/h, capillary potential 3.00 kV, cone potential 30 V. Nitrogen was used as both nebulizing and drying gas. The data were obtained in a scan mode ranging from 50 to 1000 *m*/*z* in 0.5 s time intervals. Data acquisition software was MassLynx V 4.2 (Waters).

### 2.3 HRMS

High-resolution mass spectrometry (HRMS) measurements were performed using a Synapt G2-Si mass spectrometer (Waters) equipped with an ESI source and a quadrupole-Time-of-Flight mass analyzer. The mass spectrometer was operated in the positive ion detection mode. Optimized source parameters: capillary voltage 3.1 kV, cone voltage 40 V, source temperature 120 °C, desolvation gas (nitrogen) flow rate 600 L/h at 350 °C, nebulizer gas pressure 6.5 bar. To ensure accurate mass measurements, data were collected in the centroid mode, and mass was corrected during acquisition using leucine encephalin solution as an external reference, Lock-SprayTM (Waters Corp., Milford, MA, USA), which generated the reference ion at *m*/*z* 556.2771 Da ([M + H]^+^) in the positive ESI mode. Measurement results were processed using MassLynx 4.1 software (Waters).

#### 2.3.1 General procedure for the synthesis of intermediates **9**, **10**, **14**

A three-necked round-bottom flask was charged with 5 g (0.027 mol) cyanuric chloride **8** and it was dissolved in 50 mL anhydrous tetrahydrofuran followed by cooling to 0 °C. Subsequently, 1.1 equivalents of the appropriate amine (**6** or **7** or **13**) were added dropwise to the resulting mixture while monitoring temperature so as not to exceed 3 *°*C. The mixture was stirred for 1 h at 0 °C and subsequently the resulting solid was filtered off, and the filtrate was evaporated at 25 *°*C (higher temperatures may cause product decomposition) under reduced pressure. A beige solid was triturated with cold methanol to yield white solid products (**9**, **10**, **14**) which did not need further purification.

#### 2.3.2 General procedure for the synthesis of intermediates **11**, **12**

A round-bottom flask was charged with 0.020 mol intermediate **9** or **10** and it was dissolved in 50 mL tetrahydrofuran followed by the addition of 6 mL 25 % ammonia solution. The mixture was stirred overnight at room temperature. Subsequently, the precipitated solid was filtered off, and the filtrate was evaporated to dryness under reduced pressure. A beige solid was triturated with cold methanol to yield white solid products (**11**, **12**) which did not need further purification.

#### 2.3.3 Synthesis of *N*-benzyl-4-chloro-6-morpholino-1,3,5-triazin-2-amine (**16**)

A round-bottom flask was charged with 3 g (0.012 mol) intermediate **14** and it was dissolved in 50 mL tetrahydrofuran followed by the addition of 2.46 mL (0.014 mol) DIPEA. The mixture was cooled to 0–5 *°*C and 1.14 mL (0.013 mol) morpholine was added. Subsequently, the temperature was raised to room temperature and stirred overnight. After this time, the solvent was removed under reduced pressure and an amber-like residue was dissolved in dichloromethane and washed with 0.1 M HCl solution. The organic layer was dried over MgSO_4_ and then evaporated to dryness. 3 g (80 % yield) of a beige solid was obtained. UPLC-MS analysis: t = 7.35 min, 96.94 % purity, calc. for C_14_H_17_ClN_5_O *m*/*z* = 306.11 [M + H]^+^, found *m*/*z* = 306.15 [M + H]^+^.

#### 2.3.4 General procedure for the synthesis of compound **3** (Sonochemical, reaction number 1–54)

Intermediate **1** (0.25 g, 0.870 mmol), the base (2.610 mmol), and PTC (0.087 mmol) were ground in a mortar and transferred to a round-bottom flask or a sealed tube which was previously charged with an appropriate 2-phenylethylamine (2.175 mmol). Subsequently, 1 mL of an appropriate solvent was added. The mixture was reacted in the ultrasonic bath or the ultrasonic reactor (Sonicator VCX 130 PB) for 60 min or 5 min, respectively. Reaction progress was monitored *via* TLC (chloroform: MeOH 9:1 v/v). After this time, a sample was taken from the reaction mixture and analyzed by UPLC-MS.

For reaction 54: The mixture was diluted with chloroform, transferred to a separatory funnel and washed with aqueous 1 M HCl twice. The organic layer was washed with saturated NaCl again and then dried over MgSO_4_ and concentrated. The product as colorless sticky-like oil was then dissolved in acetone and crushed by the addition of cold diethyl ether. The white powder was filtered and rinsed with cold diethyl ether and then dried to yield compound **3**.

#### 2.3.5 N^2^–(2-(1H-indol-3-yl)ethyl)-N^4^-phenethyl-1,3,5-triazine-2,4,6-triamine Hydrochloride (**3**)

white solid, 93 % yield, mp: 203–205 °C; ^1^H NMR (400 MHz, MeOD) *δ* 7.56 (d, *J* = 7.8 Hz, 1H, ArH), 7.35 (d, *J* = 8.1 Hz, 1H, ArH), 7.32 – 7.15 (m, 5H, ArH), 7.09 (dd, *J* = 15.4, 4.7 Hz, 2H, ArH), 6.98 (dt, *J* = 34.0, 7.4 Hz, 1H, ArH), 3.79 – 3.66 (m, 2H, AlH), 3.59 (dt, *J* = 25.0, 7.2 Hz, 2H, AlH), 3.07 (br s, 2H, AlH), 2.87 (dt, *J* = 19.7, 7.3 Hz, 2H, AlH). ^13^C NMR (101 MHz, MeOD) *δ* 156.10 (ArC), 138.68 (ArC), 136.80 (ArC), 128.48 (ArC), 128.12 (ArC), 127.31 (ArC), 126.13 (ArC), 126.03 (ArC), 122.30 (ArC), 122.23 (ArC), 120.98 (ArC), 118.29 (ArC), 117.78 (ArC), 111.43 (ArC), 110.91 (ArC), 42.06 (AlC), 41.44 (AlC), 35.09 (AlC), 24.96 (AlC); UPLC-MS analysis: t = 5.98 min, 100 % purity, calc. for C_21_H_24_N_7_ *m*/*z* = 374.21 [M + H]^+^ found *m*/*z* = 374.26 [M + H]^+^, HRMS (+ESI) for pure product: *m*/*z* calc. for C_21_H_24_N_7_ 374.2093 [M + H]^+^; found 374.2089.

#### 2.3.6 General procedure for the synthesis of final compounds **3a** to **5d** (Sonochemical)

Intermediate **1** (0.25 g, 0.870 mmol), sodium carbonate 0.28 g (2.610 mmol), and TBAB (0.087 mmol) were ground in a mortar and transferred to a sealed tube which was previously charged with an appropriate amine **17**–**25** (2.175 mmol). Subsequently, 1 mL of water was added. The mixture was reacted in an ultrasonic reactor (Sonicator VCX 130 PB) for 5 min with amplitude equal to 70 %. Reaction progress was monitored *via* TLC (chloroform: MeOH 9:1 v/v). After this time, a sample was taken from the reaction mixture and analyzed by UPLC-MS. Subsequently, the mixture was diluted with chloroform, transferred to a separatory funnel and washed with aqueous 1 M HCl twice. The organic layer was washed with saturated NaCl again and then dried over MgSO_4_ and concentrated. The product as colorless or yellowish sticky-like oil was then dissolved in acetone and crushed by the addition of cold diethyl ether. The white or beige powder was filtered and rinsed with cold diethyl ether and then dried to yield products **3a**–**5d**.

#### 2.3.7 N^2^–(2-(1H-indol-3-yl)ethyl)-N^4^-(2-(thiophen-2-yl)ethyl)-1,3,5-triazine-2,4,6-triamine Hydrochloride (**3a**)

white solid, 93 % yield, mp: 166–168 °C; ^1^H NMR (400 MHz, MeOD) *δ* 7.56 (d, *J* = 7.7 Hz, 1H, Ar-H), 7.35 (d, *J* = 8.0 Hz, 1H, Ar-H), 7.22 (dd, *J* = 11.8, 5.0 Hz, 1H, Ar-H), 7.09 (dt, *J* = 15.5, 7.7 Hz, 2H, Ar-H), 7.05 – 6.78 (m, 3H, Ar-H), 3.74 (dd, *J* = 14.2, 7.1 Hz, 2H, Al-H), 3.65 (t, *J* = 7.0 Hz, 1H, Al-H), 3.58 (t, *J* = 7.0 Hz, 1H, AlH), 3.09 (dt, *J* = 11.1, 6.9 Hz, 4H, AlH); ^13^C NMR (101 MHz, MeOD) *δ* 156.12 (Ar-C), 140.69 (Ar-C), 140.55 (Ar-C), 136.80 (Ar-C), 127.32 (Ar-C), 126.52 (Ar-C), 125.15 (Ar-C), 123.53 (Ar-C), 123.45 (Ar-C), 122.31 (Ar-C), 120.97 (Ar-C), 118.28 (Ar-C), 117.79 (Ar-C), 111.41 (Ar-C), 110.91 (Ar-C), 42.16 (Al-C), 41.45 (Al-C), 29.05 (Al-C), 24.94 (Al-C); UPLC-MS analysis for crude product t = 5.74 min, 76 % of content; for pure product: t = 5.81 min, 99 % purity, calc. for C_19_H_22_N_7_S *m*/*z* = 380.16 [M + H]^+^, found *m*/*z* = 380.2 [M + H]^+^; HRMS (+ESI) for pure product: *m*/*z* calc. for C_19_H_22_N_7_S: 380.1657 [M + H]^+^; found 380.1647.

#### 2.3.8 N^2^–[2-(1H-indol-3-yl)ethyl]-N^4^-[2-(3-methoxyphenyl)ethyl]-1,3,5-triazine-2,4,6-triamine Hydrochloride (**3b**)

white solid, 73 % yield, mp: 99–103 °C; ^1^H NMR (400 MHz, MeOD) *δ* 7.55 (d, *J* = 7.9 Hz, 1H, Ar-H), 7.35 (d, *J* = 8.1 Hz, 1H, Ar-H), 7.22 – 6.87 (m, 4H, Ar-H), 6.81 (t, *J* = 8.1 Hz, 1H, Ar-H), 6.76 (dd, *J* = 9.0, 5.9 Hz, 2H, Ar-H), 3.81 – 3.73 (m, 2H, Al-H), 3.73 – 3.66 (m, 3H, OCH_3_), 3.59 (dt, *J* = 21.7, 7.2 Hz, 2H, Al-H), 3.05 (t, *J* = 7.1 Hz, 2H, Al-H), 2.84 (dt, *J* = 18.2, 7.2 Hz, 2H, Al-H); ^13^C NMR (101 MHz, MeOD) *δ* 159.86 (Ar-C), 156.03 (Ar-C), 140.22 (Ar-C), 136.79 (Ar-C), 129.11 (Ar-C), 127.31 (Ar-C), 122.29 (Ar-C), 120.99 (Ar-C), 120.82 (Ar-C), 120.79 (Ar-C), 118.30 (Ar-C), 117.79 (Ar-C), 114.20 (Ar-C), 114.14 (Ar-C), 111.48 (Ar-C), 111.40 (Ar-C), 110.92 (Ar-C), 54.13 (OCH_3_-C), 41.96 (Al-C), 41.47 (Al-C), 35.16 (Al-C), 24.95 (Al-C), 24.94 (Al-C); UPLC-MS analysis for crude product t = 5.89 min, 82 % of content; for pure product: t = 5.87 min, 98 % purity, calc. for C_22_H_26_N_7_O *m*/*z* = 404.22 [M + H]^+^, found *m*/*z* = 404.2 [M + H]^+^; HRMS (+ESI) for pure product: *m*/*z* calc. for C_22_H_26_N_7_O: 404.2199 [M + H]^+^; found 404.2192.

#### 2.3.9 N^2^–[2-(1H-indol-3-yl)ethyl]-6-(4-phenylpiperazin-1-yl)-1,3,5-triazine-2,4-diamine Hydrochloride (**3c**)

white solid, 82 % yield, mp: 160–163 °C; ^1^H NMR (400 MHz, MeOD) *δ* 7.58 (d, *J* = 7.8 Hz, 1H, Ar-H), 7.35 (d, *J* = 8.1 Hz, 1H, Ar-H), 7.28 (dd, *J* = 8.4, 7.5 Hz, 2H, Ar-H), 7.15 – 7.07 (m, 2H, Ar-H), 7.03 (dd, *J* = 10.7, 8.0 Hz, 3H, Ar-H), 6.91 (t, *J* = 7.3 Hz, 1H, Ar-H), 3.92 (d, *J* = 33.2 Hz, pip-4H), 3.78 (t, *J* = 6.7 Hz, 2H, Al-H), 3.25 – 3.06 (m, 6H − pip and overlapped Al-H); ^13^C NMR (101 MHz, MeOD) *δ* 156.47 (Ar-C), 150.75 (Ar-C), 136.84 (Ar-C), 128.79 (3Ar-C), 127.34 (Ar-C), 122.45 (Ar-C), 121.04 (2Ar-C), 120.48 (Ar-C), 118.28 (Ar-C), 117.69 (Ar-C), 116.67 (2Ar-C), 111.28 (Ar-C), 111.01 (Ar-C), 49.28 (Al-C), 43.44 (Al-C), 41.18 (Al-C), 24.76 (Al-C); UPLC-MS analysis for pure product: t = 6.54 min, 98 % purity, calc. for C_23_H_27_N_8_ *m*/*z* = 414.23 [M + H]^+^, found *m*/*z* = 415.2 [M + H]^+^; HRMS (+ESI) for pure product: *m*/*z* calc. for C_23_H_27_N_8_: 415.2359 [M + H]^+^; found 415.2352.

#### 2.3.10 N^2^–{2-[(4-fluorophenyl)amino]ethyl}-N^4^-[2-(1H-indol-3-yl)ethyl]-1,3,5-triazine-2,4,6-triamine hydrochloride (**3d**)

white solid, 91 % yield, mp: 149–153 °C; ^1^H NMR (400 MHz, MeOD) *δ* 7.65 – 7.55 (m, 2H, Ar-H), 7.53 – 7.47 (m, 1H, Ar-H), 7.34 (dd, *J* = 13.4, 8.4 Hz, 2H, Ar-H), 7.20 (t, *J* = 8.5 Hz, 1H, Ar-H), 7.11 (dd, *J* = 15.7, 10.0 Hz, 2H, Ar-H), 7.06 – 6.99 (m, 1H, Ar-H), 3.80 – 3.67 (m, 4H, Al-H), 3.63 – 3.48 (m, 2H, Al-H), 3.11 – 3.02 (m, 2H, Al-H); ^13^C NMR (101 MHz, MeOD) *δ* 156.18 (Ar-C), 148.08 (Ar-C), 136.79 (Ar-C), 131.46 (Ar-C), 127.29 (Ar-C), 124.50 (Ar-C), 124.38 (Ar-C), 124.29 (Ar-C), 122.46 (Ar-C), 121.04 (Ar-C), 118.32 (Ar-C), 117.71 (Ar-C), 116.99 (Ar-C), 116.76 (Ar-C), 111.25 (Ar-C), 111.02 (Ar-C), 110.93 (Ar-C), 49.66 (2Al-C), 41.35 (2Al-C), 36.59 (Al-C), 24.79 (Al-C); UPLC-MS analysis for pure product: t = 5.82 min, 98 % purity, calc. for C_21_H_24_N_8_F *m*/*z* = 407.2 [M^+^], found *m*/*z* = 407.3 [M + H]^+^; HRMS (+ESI) for pure product: *m*/*z* calc. for C_21_H_24_N_8_F: 407.2108 [M + H]^+^; found 407.2101.

#### 2.3.11 N^2^-phenyl-N^4^-(2-phenylethyl)-1,3,5-triazine-2,4,6-triamine hydrochloride (**4a**)

white solid, 66 % yield, mp: 222–225 °C; ^1^H NMR (400 MHz, DMSO) *δ* 10.35 (bp, 1H, N-H), 8.67 (bp, 1H, N-H), 8.14 (bp, 2H, N-H), 7.65 (d, *J* = 7.3 Hz, 2H, Ar-H), 7.38 – 7.28 (m, 5H, Ar-H), 7.23 (d, *J* = 6.9 Hz, 2H, Ar-H), 7.13 (s, 1H, Ar-H), 3.56 (br s, 2H, Al-H), 2.9–2.81 (m, 2H, Al-H); ^13^C NMR (101 MHz, DMSO) *δ* 139.17 (Ar-C), 129.28 (Ar-C), 129.17 (2Ar-C), 129.10 (Ar-C), 129.05 (2Ar-C), 128.85 (2Ar-C), 128.79 (2Ar-C), 126.77 (Ar-C), 126.70 (Ar-C), 124.58 (Ar-C), 122.13 (Ar-C), 42.50 (Al-C), 35.06 (Al-C).; UPLC-MS analysis for crude product t = 5.70 min, 53 % of content; for pure product: t = 5.61 min, 91 % purity, calc. for C_17_H_19_N_6_ *m*/*z* = 307.2 [M + H]^+^, found *m*/*z* = 307.2 [M + H]^+^; HRMS (+ESI) for pure product: *m*/*z* calc. for C_17_H_19_N_6_: 307.1671 [M + H]^+^; found 307.1664.

#### 2.3.12 N^2^–[2-(3-methoxyphenyl)ethyl]-N^4^-phenyl-1,3,5-triazine-2,4,6-triamine hydrochloride (**4b**)

white solid, 67 % yield, mp: 214–216 °C; ^1^H NMR (400 MHz, DMSO) *δ* 8.53 (bp, 1H, N-H), 8.18 (bp, 1H, N-H), 7.66 (s, 2H, Ar-H), 7.34 (s, 2H, Ar-H), 7.17 (d, *J* = 37.6 Hz, 2H, Ar-H), 6.78 (s, 3H, Ar-H), 3.74 (s, 3H, OCH_3_), 3.57 (s, 2H, Al-H), 2.84 (s, 2H, Al-H); ^13^C NMR (101 MHz, DMSO) *δ* 169.09 (Ar-C), 167.44 (Ar-C), 159.79 (Ar-C), 159.75 (Ar-C), 140.72 (Ar-C), 138.31 (Ar-C), 129.87 (Ar-C), 129.77 (Ar-C), 129.17 (Ar-C), 129.05 (Ar-C), 124.55 (Ar-C), 122.06 (Ar-C), 121.95 (Ar-C), 121.51 (Ar-C), 121.40 (Ar-C), 114.87 (Ar-C), 114.74 (Ar-C), 112.22 (Ar-C), 55.43 (OCH_3_), 42.28 (Al-C), 35.11 (Al-C); UPLC-MS analysis for crude product t = 5.66 min, 76 % of content; for pure product: t = 5.67 min, 88 % purity, calc. for C_18_H_21_N_6_O *m*/*z* = 337.2 [M + H]^+^, found *m*/*z* = 337.2 [M + H]^+^; HRMS (+ESI) for pure product: *m*/*z* calc. for C_18_H_21_N_6_O: 337.1777 [M + H]^+^; found 337.1771.

#### 2.3.13 N^2^–(3-chlorophenyl)-N^4^-[2-(4-fluorophenyl)ethyl]-1,3,5-triazine-2,4,6-triamine hydrochloride (**4c**)

white solid, 87 % yield, mp: 206–208 °C; ^1^H NMR (400 MHz, DMSO) *δ* 10.42 (bp, 1H, N-H), 8.72 (bp, 1H, N-H), 8.26 (bp, 2H, N-H), 7.86 (s, 1H, Ar-H), 7.61 (d, *J* = 8.5 Hz, 1H, Ar-H), 7.40 – 7.24 (m, 3H, Ar-H), 7.18 – 7.07 (m, 3H, Ar-H), 3.61–3.50 (m, 2H, Al-H), 2.86 (t, *J* = 6.6 Hz, 2H, Al-H); ^13^C NMR (101 MHz, DMSO) *δ* 162.60 (Ar-C), 160.20 (Ar-C), 140.08 (Ar-C), 135.27 (Ar-C), 133.40 (Ar-C), 131.14 (Ar-C), 131.05 (Ar-C), 130.97 (Ar-C), 130.66 (Ar-C), 124.02 (Ar-C), 121.36 (Ar-C), 121.16 (Ar-C), 120.21 (Ar-C), 115.60 (Ar-C), 115.39 (Ar-C), 42.42 (Al-C), 34.19 (Al-C); UPLC-MS analysis for crude product t = 6.41 min, 87 % of content; for pure product: t = 6.43 min, 98 % purity, calc. for C_17_H_17_N_6_FCl *m*/*z* = 359.1 [M + H]^+^, found *m*/*z* = 359.2 [M + H]^+^; HRMS (+ESI) for pure product: *m*/*z* calc. for C_17_H_17_N_6_FCl: 359.1187 [M + H]^+^; found 359.1181.

#### 2.3.14 N^2^–(3-chlorophenyl)-6-(4-phenylpiperazin-1-yl)-1,3,5-triazine-2,4-diamine hydrochloride (**4d**)

white solid, 79 % yield, mp: 146–148 °C; ^1^H NMR (400 MHz, DMSO) *δ* 10.91 (bp, 1H, N-H), 8.37 (bp, 2H, N-H), 7.76 (s, 1H, Ar-H), 7.59 (d, *J* = 8.2 Hz, 1H, Ar-H), 7.41 (t, *J* = 8.1 Hz, 1H, Ar-H), 7.35 (t, *J* = 7.8 Hz, 2H, Ar-H), 7.29 (d, *J* = 7.5 Hz, 2H, Ar-H), 7.21 (dd, *J* = 8.0, 1.1 Hz, 1H, Ar-H), 7.04 (t, *J* = 6.9 Hz, 1H, Ar-H), 4.08 (s, 4H, Al-H), 3.49 (d, *J* = 57.1 Hz, 4H, Al-H); ^13^C NMR (101 MHz, DMSO) *δ* 157.86 (Ar-C), 148.42 (Ar-C), 148.18 (Ar-C), 139.31 (2Ar-C), 133.50 (2Ar-C), 130.97 (2Ar-C), 129.80 (Ar-C), 124.47 (Ar-C), 123.01 (Ar-C), 121.49 (Ar-C), 120.37 (Ar-C), 118.09 (Ar-C), 49.91 (2Al-C), 43.50 (2Al-C).; UPLC-MS analysis for pure product: t = 7.23 min, 98 % purity, calc. for C_19_H_19_N_7_Cl *m*/*z* = 380.14 [M−H]^−^, found *m*/*z* = 380.31 [M−H]^−^; HRMS (+ESI) for pure product: *m*/*z* calc. for C_19_H_21_N_7_Cl: 382.1547 [M + H]^+^; found 382.1541.

#### 2.3.15 N^2^-benzyl-6-(morpholin-4-yl)-N^4^-(2-phenylethyl)-1,3,5-triazine-2,4-diamine hydrochloride (**5a**)

white solid, 81 % yield, mp: 165–170 °C; ^1^H NMR (400 MHz, MeOD) *δ* 7.36 (d, *J* = 4.2 Hz, 4H, Ar-H), 7.33 – 7.20 (m, 6H, Ar-H), 4.60 (s, 2H, Al-CH_2_), 3.86 (br s, 4H, morf), 3.74 – 3.68 (m, 6H, morf and overlapped Al-H), 2.92 (t, *J* = 7.0 Hz, 2H, Al-H); ^13^C NMR (101 MHz, MeOD) *δ* 161.45 (Ar-C), 154.69 (Ar-C), 138.55 (Ar-C), 137.53 (Ar-C), 128.51 (2Ar-C), 128.28 (2Ar-C), 128.21, (2Ar-C) 127.29 (2Ar-C), 127.25 (2Ar-C), 127.02 (Ar-C), 126.19 (Ar-C), 66.09 (2Al-C), 44.34 (2Al-C), 44.08 (Al-C), 41.83 (Al-C), 34.80 (Al-C); UPLC-MS analysis for crude product t = 6.49 min, 81 % of content; for pure product: t = 6.49 min, 95 % purity, calc. for C_22_H_27_N_6_O *m*/*z* = 391.2 [M + H]^+^, found *m*/*z* = 391.2 [M + H]^+^; HRMS (+ESI) for pure product: *m*/*z* calc. for C_22_H_27_N_6_O: 391.2246 [M + H]^+^; found 391.2238.

#### 2.3.16 4-[4-(1-benzothiophen-4-yl)piperazin-1-yl]-N-benzyl-6-(morpholin-4-yl)-1,3,5-triazin-2-amine hydrochloride (**5b**)

white solid, 65 % yield, mp: 124–128 °C; ^1^H NMR (400 MHz, MeOD) *δ* 7.95 – 7.67 (m, 3H, Ar-H), 7.45 – 7.34 (m, 6H, Ar-H), 7.30 (d, *J* = 7.1 Hz, 1H, Ar-H), 4.71 – 4.66 (m, 2H, Al-CH_2_), 4.37 (br s, 2H, Al-H), 4.15 (br s, 1H, Al-H), 3.93 – 3.70 (m, 9H, Al-H), 3.63 (s, 2H, Al-H), 3.48 (s, 2H, Al-H); ^13^C NMR (101 MHz, MeOD) *δ* 161.59 (Ar-C), 161.23 (Ar-C), 155.80 (Ar-C), 153.96 (Ar-C), 142.30 (Ar-C), 137.36 (Ar-C), 132.61 (Ar-C), 128.34 (Ar-C), 128.01 (Ar-C), 127.29 (Ar-C), 124.65 (Ar-C), 121.46 (Ar-C), 120.18 (Ar-C), 119.45 (Ar-C), 114.44 (Ar-C), 66.13 (Al-C), 65.58 (Al-C), 53.10 (Al-C), 51.89 (Al-C), 42.49 (Al-C); UPLC-MS analysis for crude product t = 8.38 min, 65 % of content; for pure product: t = 8.38 min, 92 % purity, calc. for C_27_H_30_N_6_OS *m*/*z* = 488.2 [M + H]^+^, found *m*/*z* = 488.2 [M + H]^+^; HRMS (+ESI) for pure product: *m*/*z* calc. for C_26_H_30_N_7_SO: 488.2233 [M + H]^+^; found 488.2226.

#### 2.3.17 N^2^-benzyl-N^4^-[2-(4-fluorophenyl)ethyl]-6-(morpholin-4-yl)-1,3,5-triazine-2,4-diamine hydrochloride (**5c**)

white solid, 78 % yield, mp: 174–178 °C; ^1^H NMR (400 MHz, MeOD) *δ* 7.36 (d, *J* = 4.2 Hz, 4H, Ar-H), 7.33 – 7.23 (m, 3H, Ar-H), 7.02 (dd, *J* = 18.5, 9.8 Hz, 2H, Ar-H), 4.60 (s, 2H, Al-CH_2_), 3.83–3.78 (m, 4H, Al-H), 3.74–3.66 (m, 6H, Al-H), 2.91 (t, *J* = 6.9 Hz, 2H, Al-H); ^13^C NMR (101 MHz, MeOD) *δ* 162.93 (Ar-C), 161.43 (Ar-C), 160.51 (Ar-C), 154.84 (Ar-C), 154.68 (Ar-C), 137.53 (Ar-C), 134.53 (Ar-C), 130.28 (Ar-C), 130.20 (Ar-C), 128.28 (2Ar-C), 127.31 (Ar-C), 127.24 (Ar-C), 114.88 (Ar-C), 114.67 (Ar-C), 66.09 (2Al-C), 44.33 (2Al-C), 44.07 (Al-C), 41.81 (Al-C), 33.95 (Al-C); UPLC-MS analysis for pure product: t = 6.52 min, 98 % purity, calc. for C_22_H_26_N_6_FO *m*/*z* = 409.2 [M + H]^+^, found *m*/*z* = 409.2 [M + H]^+^; HRMS (+ESI) for pure product: *m*/*z* calc. for C_22_H_26_N_6_FO: 409.2152 [M + H]^+^; found 409.2146.

#### 2.3.18 N^2^-benzyl-N^4^-[2-(2-chlorophenoxy)ethyl]-6-(morpholin-4-yl)-1,3,5-triazine-2,4-diamine hydrochloride (**5d**)

white solid, 82 % yield, mp: 178–180 °C; ^1^H NMR (400 MHz, MeOD) *δ* 7.36 (d, *J* = 4.0 Hz, 4H, Ar-H), 7.32 – 7.23 (m, 2H, Ar-H), 7.01 – 6.94 (m, 2H, Ar-H), 6.91 (d, *J* = 8.3 Hz, 1H, Ar-H), 4.62 (s, 1H, Al-CH_2_), 4.20 (t, *J* = 5.2 Hz, 2H), 3.86 (br s, 6H, morf), 3.70 (br s, 4H, morf overlapped with Al-H); ^13^C NMR (101 MHz, MeOD) *δ* 161.46 (Ar-H), 159.40 (Ar-H), 155.13 (Ar-H), 154.70 (Ar-H), 137.49 (Ar-H), 134.55 (Ar-H), 130.27 (Ar-H), 128.29 (Ar-H), 127.31 (Ar-H), 127.26 (Ar-H), 120.83 (Ar-H), 114.63 (Ar-H), 112.80 (Ar-H), 66.07 (Al-H), 65.94 (Al-H), 44.34 (Al-H), 44.12 (Al-H), 39.81 (Al-H). UPLC-MS analysis for crude product t = 6.60 min, 49 % of content; for pure product: t = 6.95 min, purity 98 %, calc. for C_22_H_26_ClN_6_O_2_ *m*/*z* = 441.2 found *m*/*z* = 441.2 [M + H]^+^; HRMS (+ESI) for pure product: *m*/*z* calc. for C_22_H_26_N_6_O_2_Cl: 441.1806 [M + H]^+^; found 441.1800.

## 3 Results and discussion

Intermediate **1** was obtained according to our previous procedure.[17] In the first stage (Scheme 3, Table 2), the type of the base used (organic and inorganic) and its influence on the yield of the product – N^2^–(2-(1H-indol-3-yl)ethyl)-N4-phenethyl-1,3,5-triazine-2,4,6-triamine (**3**) – was assessed. The tested bases were: potassium carbonate (K_2_CO_3_), sodium hydroxide (NaOH), potassium hydroxide (KOH), triethylamine (TEA), proton sponge (PS), 1,4-diazabicyclo[2.2.2]octane (DABCO), sodium carbonate (Na_2_CO_3_), N,N-diisopropylethylamine (DIPEA), pyridine, ammonia solution (NH3 aq.), and 1,8-Diazabicyclo[5.4.0]undec-7-ene (DBU). The constant features included the use of a phase-transfer catalyst (0.1 eq. tetrabutylammonium bromide) and 1 mL of three types of solvents: DMF, ACN and water. The reaction was conducted using an ultrasonic bath with a power of P = 60 W over 1 h.

**Table 2:** The impact of the various bases used in the model reaction on the yield of compound 3.

Reaction No	base	solvent [1 mL]	yield [%]*
1	K_2_CO_3_	DMF	96
2		ACN	99
3		water	97
4	NaOH	DMF	6
5		ACN	44
6		water	100
7	KOH	DMF	4
8		ACN	35
9		water	93
10	TEA	DMF	59
11		ACN	91
12		water	95
13	PS	DMF	40
14	DABCO	DMF	70
15		ACN	64
16		water	60
17	Na_2_CO_3_	DMF	59
18		ACN	100
19		water	98
20	DIPEA	DMF	61
21		ACN	90
22		water	94
23	pyridine	DMF	31
24		ACN	96
25		water	100
26	NH_3_ aq.	DMF	85
27		ACN	81
28		water	83
29	DBU	DMF	81
30		ACN	90
31		water	63

* Yield determined by the analysis of the post-reaction mixture composition using LC-MS;

Based on the results from Table 2 it is possible to obtain compound **3** with a good or very good yield under the conditions described above. Favorable outcomes (yields between 50 % and 90 %) were achieved when using e.g. 25 % ammonia solution, DABCO or DBU. In this case, the influence of the solvent on the reaction yield was generally marginal, but DMF seemed to be more preferable. The best results (yields mostly equal to or more than 90 %) were obtained using DIPEA, TEA, pyridine, sodium carbonate, or potassium carbonate as the base. Apart from potassium carbonate, DMF was a less preferable solvent than the other ones. With strong inorganic bases (NaOH and KOH), reactions in DMF resulted in the lowest product yield again, while using water gave 100 % or 93 % yield, respectively. Surprisingly, analysis of the type of the used solvents shows that in general 1 mL of water proved to be the most effective among the investigated solvents.

Subsequently, the expanded influence of various solvents on the yield of compound **3** was evaluated. When selecting the solvent, emphasis was put on environmental aspects, choosing only those specifically labeled by GlaxoSmithKline as 'green', such as glycerol, ethylene glycol, methanol, and 1-butyl-3-methylimidazolium tetrafluoroborate ([bmim]BF4).[14] When selecting bases, we chose K_2_CO_3_, Na_2_CO_3_ and NH_3_ aq. only. The choice was dictated by the yields obtained from Table 2, as well as technological and environmental aspects. The other parameters, such as PTC, ultrasound bath power and the reaction time, were identical as before.

Analysis of the results from Table 3 shows that compound **3** was generally obtained in a high or very high yield (more than 80 %), apart from reaction 39 in which methanol in the presence of NH_3_ aq. was used (reaction yield: 37 %). The effectiveness of all tested solvents was comparable to using water, which indicates that these solvents can be a green alternative to water. However, considering the low cost of water and the ease of technological operations, such as extraction using water, it remains the most attractive option and further tests will be conducted using water. Similar conclusions are made regarding the type of the base used: although ammonia provided a high yield, it might pose technological challenges. Additionally, its toxicity and irritant properties require extra measures for its disposal from the post-reaction mixture. Hence, sodium carbonate has been chosen for further tests.

**Table 3:** The impact of the various bases used in the model reaction on the yield of compound 3.

Reaction No	base	solvent [1 mL]	yield [%]*
32	K_2_CO_3_	glycerin	96
33		ethylene glycol	96
34		methanol	96
35		[bmim]BF_4_	95
36	NH_3_ aq.	glycerin	86
37		ethylene glycol	84
38		methanol	37
39		[bmim]BF_4_	91
40	Na_2_CO_3_	glycerin	88
41		ethylene glycol	96
42		methanol	97
43		[bmim]BF_4_	94

* Yield determined by the analysis of the post-reaction mixture composition using LC-MS.

The next step involved assessing the impact of various PTCs in an amount of 0.1 equivalents on the yield of compound **3**. The study primarily focused on quaternary ammonium salts: benzyltriethylammonium chloride (TEBA), benzyltributylammonium chloride (BTBAC), cetyltrimethylammonium bromide (CTAB), tetramethylammonium bromide (TMAB), tetraethylammonium chloride (TEAC); crown ether (18-crown-6), and potassium iodide (KI). Reactions were carried out in the presence of Na_2_CO_3_ and water using an ultrasound bath with a power of P = 60 W over 1 h.

The results presented in Table 4 indicate that all the tested PTCs can be used for the synthesis of compound **3** with a very high yield. For the remaining PTCs, the product was obtained with yields ranging from 91 % to 97 %. Notably, the use of TBAB achieved the highest efficiency of 98 % (reaction number: 19).

**Table 4:** The impact of the various PTCs used in the model reaction on the yield of compound 3.

Reaction No	PTC	yield [%]*
44	TEBA	94
45	TEAC	97
46	TMAB	91
47	CTAB	90
48	BTBAC	94
49	Crown-18	96
50	KI	96

* Yield determined by the analysis of the post-reaction mixture composition using LC-MS.

To corroborate the hypothesis that ultrasound application positively influences the acceleration of the synthesis of compound **3**, a control experiment was conducted utilizing Na_2_CO_3_, TBAB, and 1 mL of water, devoid of ultrasonic application (Table 5). Predictably, the reaction's yield in the absence of ultrasonic stimulation was limited to 56 %, conclusively validating the hypothesis that ultrasonic activation enhances the reaction yield.

**Table 5:** Synthesis of compound 3 without ultrasound application.

Reaction No	Conditions	time [min]	yield [%]*
51	Na_2_CO_3_, TBAB, 1 mL H2O	60	56

* Yield determined by the analysis of the post-reaction mixture composition using LC-MS.

Finally, the experiments involved the use of different sources of ultrasonic energy: an ultrasonic reactor (with an amplitude of A = 70 %, and a power of P = 5–7 W) was used. The best conditions obtained so far were selected for the experiments. The first experiment was conducted using an ultrasonic bath with a shorter synthesis time, while subsequent experiments were carried out using an ultrasonic reactor. The results are presented in Table 6.

**Table 6:** The impact of the applied source of ultrasonic energy and synthesis time on the yield of compound 3. Conditions: sodium carbonate, TBAB and water.

Reaction No	ultrasound source	time [min]	yield [%]*
52	ultrasonic bath	20	68
53	ultrasonic reactor	2	88
54	ultrasonic reactor	5	97 93**

* Yield determined by the analysis of the post-reaction mixture composition using LC-MS.

** Yield determined by the quantity of the isolated final product.

Reducing the reaction time from 60 min (Table 2, reaction 19, yield = 98 %) to 20 min (Table 6, reaction 52, yield = 68 %) resulted in a significant decrease in the reaction yield. Simultaneously, reactions conducted using the ultrasonic reactor proved to be more efficient and faster than those using the ultrasonic bath. Within 2 min (Table 6, reaction 53), product **3** was obtained with a yield of 88 %. Extending the synthesis time by additional 3 min (Table 6, reaction 54) led to a further increase in the yield of up to 97 %. After pH-dependent work-up only, the final product **3** was isolated with a yield of 93 % and further purification was not required.

Usefulness of the previously developed method using the ultrasonic reactor was evaluated by synthesizing a new library of 1,3,5-triazine derivatives (Table 7). The 1,3,5-triazine compounds contained three leading scaffolds: tryptamine (**3a**-**3f**), aniline (**4a**-**4d**) and benzylamine (**5a**-**5d**) were considered. The tryptamine scaffold has been extensively examined by our team due to its 5-HT_7_ receptor binding profile and potential usefulness in the treatment of CNS diseases.[17], [21], [22] Aniline or benzylamine scaffolds may be used to design new compounds with anticancer activity.[25].

**Table 7:** Example compounds obtained using the developed sonochemistry method. The yield was determined based on the analysis of the post-reaction mixture composition using LC-MS.

Tryptamines	Y [%]	Anilines	Y [%]	Benzylamines	Y [%]
	76		53		81
**3a**		**4a**		**5a**	
	82		57		65
**3b**		**4b**		**5b**	
	82*		87		78*
**3c**		**4c**		**5c**	
	91		79*		82*
**3d**		**4d**		**5d**	
	49				
**3e**					
	78				
**3f**					

* Yield determined by the quantity of the isolated final product.

**Scheme 4:**
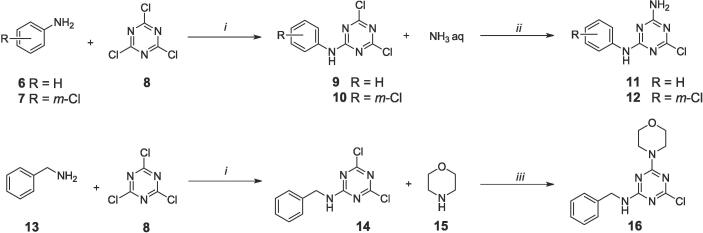
Synthesis of intermediates 11, 12, 16. *i* – THF, 0 °C; *ii* – THF, room temp.; *iii* – DIPEA, THF, room temp.

While the synthesis of intermediate **1** was described previously, the synthesis of aniline/benzylamine intermediates was performed according to Scheme 4. Aniline **6**, *m*-chloroaniline **7** or benzamine **13** was reacted with cyanuric chloride **8** in THF at 0 °C to give products **9**, **10**, **14**, respectively. Subsequently, products **9** and **10** were *N*-alkylated with ammonia water, while product **14** was reacted with morpholine in the presence of DIPEA, providing intermediates **11**, **12**, **16**, respectively.

**Scheme 5:**
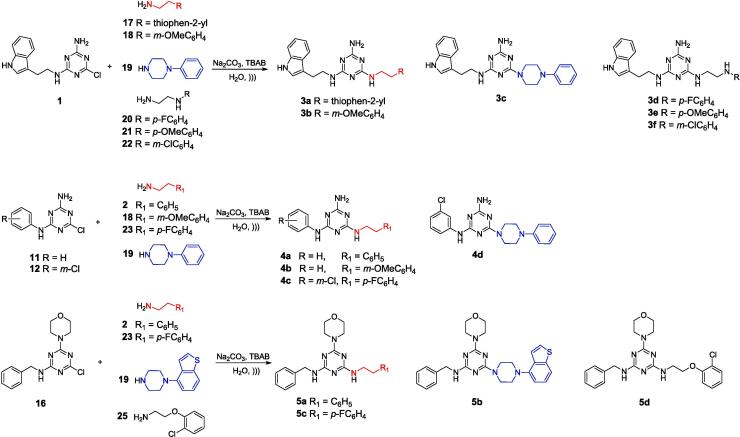
Synthesis of final compounds 3a-3f, 4a-4d, 5a-5d whose exact structural formulas are presented in Table 7.

Subsequently, according to Scheme 5, intermediates **1**, **11**, **12**, **16** were reacted with commercially available amines **2**, **17**–**25** under ultrasonic conditions using a molar ratio of substrates of 1 (tryptamine/aniline/benzylamine scaffold) to 2.5 (amines). Sodium carbonate was used as the base while the PT catalyst was TBAB. Reactions were carried out in 1 mL of water over 5 min. All final compounds were isolated from the post-reaction mixture *via* pH-dependent extraction only, showing in most cases more than 95 % purity according to LC-MS. Compounds **3e** and **3f** were analyzed by LC-MS only. According to Table 7 most of the compounds were obtained with more than 75 % yield suggesting that the developed sonochemical protocol was universal.

We described the synthesis of 1,3,5-triazine with a tryptamine scaffold (**3d**, **3e**, **3f**), primarily using potassium carbonate as the base under microwave irradiation.[22] Unfortunately, under these conditions, it was not possible to obtain the desired compounds, but replacing potassium carbonate with sodium carbonate resulted in the formation of the desired compound in good yields (more than or equal to 60 %). Herein, we decided to investigate whether the sonochemistry approach using potassium carbonate and sodium carbonate could provide desired compounds thus making this procedure more universal. It turned out that in both tested bases, compounds **3d**, **3e** and **3f** were formed (Table S1, Appendix A), which may suggest that the sonochemistry approach is more versatile and efficient than microwave irradiation. In general, using sodium carbonate rather than potassium carbonate also seems to be more preferable because of the higher yield of resulting products.

**Fig. 1:**
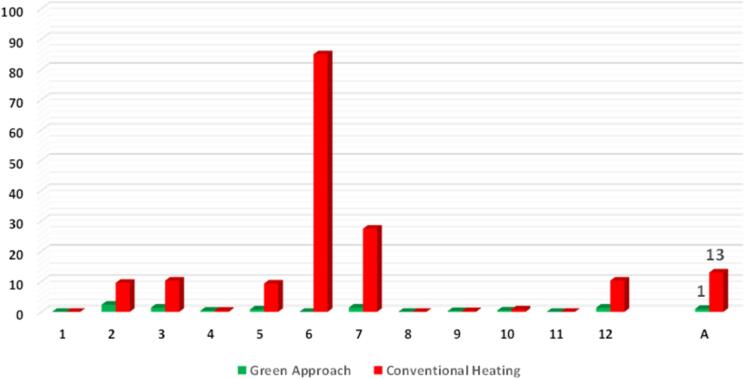
Comparison of the greenness of the “Green Approach” and “Conventional Heating” assessed by the DOZN^TM^ 2.0 tool. 12 green chemistry principles: 1. Prevention; 2. Atom Economy; 3. Less Hazardous Chemical Synthesis; 4. Designing Safer Chemicals; 5. Safer Solvents and Auxiliaries; 6. Design for Energy Efficiency; 7. Use of Renewable Feedstocks; 8. Reduce Derivatives; 9. Catalysis; 11. Real-time analysis for Pollution Prevention; 12. Inherently Safer Chemistry for Accident Prevention. A – Aggregate.

To assess whether the new approach had positive contribution to green chemistry principles, compound **3** was obtained according to two different methods. The first one (the “green approach”) was described above, and the second one was “conventional heating” using an oil bath according to our previous paper.[17] Conventional heating required the use of 5 eq. of amine **2** and 25 g DMF as the solvent and a long reaction time of 16 h. Product **3** was isolated in these conditions with 69 % yield, while the yield when using the green approach was higher (93 %). To evaluate changes between these two methods with respect to the 12 green chemistry principles, the DOZN^TM^ 2.0 tool was used.[26] The 12 green chemistry principle scores were calculated for the green approach, followed by conventional heating. According to Fig. 1, the green approach is more eco-friendly.

The most visible changes were noted for the 6^th^ principle related to energy efficiency. Reduced reaction times (from 16 h to 5 min) as well as conducting the reaction at room temperature brought forth dramatic improvement of the green aspect of the process (due to the reduced carbon footprint and water footprint). The other improved principle scores were: 2^nd^ (atom economy) – owing to a reduced amount of amine 6 and forming by-products – or 3^rd^ (less hazardous chemical synthesis) – owing to using water (a safer reagent) and reduced formation of by-products which may ultimately be hazardous; 7^th^ (using renewable feedstock) – owing to using renewable water instead of DMF. The parameter described by the “aggregate score” (A) shows how “green” the process is. A lower score means a “greener” process. According to the green approach, the aggregate score is 1, meaning that this approach is 13 times more environmentally friendly than conventional heating.

Considering the potential applicability in medicinal chemistry, lipophilicity, drug-plasma protein binding, and phospholipid affinity assays were performed using protocols developed in GlaxoSmithKline by Valko et al.[27], [28], [29] and adopted in our laboratory.[30], [31] Briefly, these methods involved determination of the Chromatographic Hydrophobicity Index (CHI), which can be easily converted to log, and % of plasma protein binding (%PPB) using one gradient elution experiment and comparison to reference substances. The experiments are described in detail in Appendix B together with raw data. The lipophilicity of molecules is a crucial physicochemical property that significantly affects their pharmacokinetics and safety. Our results indicated that the analyzed molecules had relatively high lipophilicity. Certain molecules, **4d**, **5a**, **5b**, and **5c**, had the CHI on the scale (up to 100), so they could accumulate excessively in the human body. Nevertheless, other investigated derivatives showed lipophilicity, which can be compared with commercially available CNS active drugs.[29] This is an important finding since lipophilicity is a well-known factor that determines passive diffusion through the blood–brain barrier (BBB). Generally, the target molecules had a strong affinity to phospholipids, similar to their lipophilicity, except for **5d**, which had significantly lower CHI_IAM_. Notably, only the most lipophilic structures, **4d**, **5b**, and **5c**, exceeded the value of 50 CHI_IAM_, which is the cut-off point indicating potential for promiscuous binding and affecting phospholipidosis.[29], [32] Comparing CHI values at different pH levels enables quick assessment of acid-base properties, and the analyzed substances mostly had weakly basic properties. Columns modified by plasma protein, such as human serum albumin (HAS), can also be used to estimate binding to plasma proteins (PPB). PPB affects drug pharmacokinetics, such as distribution, half-life, and clearance. Although the target substances had high %HSA binding, predominantly to PP (plasma protein), these results were still acceptable since several substances used in clinical practice show binding of up to approximately 99 %.[29].

## 4 Conclusion

Taking into account the fact that 1,3,5-triazines exhibit many interesting properties especially in medicinal chemistry and pharmacy, developing their efficient, economic and environmental friendly synthetic method is highly desirable. Herein we described a new approach for the *N*-alkylation reaction leading to 1,3,5-triazines. In the model reaction we examined various bases (organic and inorganic), solvents (from the group of “green” solvents and DMF, ACN), PTCs (mainly quaternary ammonia salts) and an ultrasound source (the ultrasonic batch and the ultrasonic reactor). It turned out that almost all bases provided model compound **3** with a good or very good yield, but the type of solvent was the differentiating factor. In nearly all examples wherein water served as the solvent, the resultant yields were maximized. From a number of potential bases, sodium carbonate was chosen as the best one because of the high obtained yields and its safety for environment. We examined other so-called “green solvents” and it turned out that they could be used as well, but required more operations during the synthesis process (such as evaporation or disposal of post-reaction solvents). Therefore, water is at this point the cheapest, the easiest to remove after the process and the safest for the environment. In addition, we reduced the reaction time from 60 min to 5 min by using the ultrasonic reactor. We confirmed the usefulness of the developed method by the synthesis of new 1,3,5-triazine derivatives with three scaffolds: tryptamine, aniline and benzylamine. Almost all compounds were obtained with yields of more than 75 % according to LC-MS analysis of the post-reaction mixture or the isolated product. Isolation of pure compounds did not require column chromatography. High yields and easy pH-dependent extraction suggest that new derivatives of 1,3,5-triazine can be obtained in a rapid, universal, economical, and environmentally friendly manner. Furthermore, the developed universal synthesis method may be incorporated into the principles of green chemistry. According to the DOZN^TM^ 2.0 tool, the newly developed synthetic method is 13 times more eco-friendly than conventional heating. Another advantage of the sonochemistry approach is that it is more versatile than e.g. microwave synthesis. We proved that using the ultrasonic reactor it was possible to obtain compounds **3d**, **3e**, **3f** in the presence of potassium carbonate, which was not possible when microwave irradiation was used. Using sodium carbonate, yields were even considerably higher. The physicochemical properties of the three proposed scaffolds suggest that they could be sustainable starting points in the drug discovery pipeline. No significant differences in lipophilicity or affinity to HSA or phospholipids were observed among the compounds tested.

## CRediT authorship contribution statement

**Damian Kułaga:** Writing – review & editing, Formal analysis, Data curation. **Anna K. Drabczyk:** Writing – review & editing, Visualization, Software, Data curation. **Przemysław Zaręba:** Writing – review & editing, Formal analysis, Data curation. **Jolanta Jaśkowska:** Writing – review & editing. **Julia Chrzan:** Data curation. **Katarzyna Ewa Greber:** Formal analysis, Data curation. **Krzesimir Ciura:** Writing – review & editing, Writing – original draft, Formal analysis, Data curation. **Damian Plażuk:** Writing – review & editing, Formal analysis, Data curation. **Ewelina Wielgus:** Formal analysis, Data curation.

## Declaration of competing interest

The authors declare that they have no known competing financial interests or personal relationships that could have appeared to influence the work reported in this paper.

## Appendix A Supplementary data

The following are the Supplementary data to this article: The authors have cited additional references within the Appendix A. In addition, Appendix A contains: all spectra, physiochemical properties of intermediates, CHIIAM and PPB/HAS procedure; Appendix B: CHIIAM and PPB/HAS binding results.
