# Supplementary material for: Green synthesis of 1,3,5-triazine derivatives using a sonochemical protocol
Source: Ultrason Sonochem. 2024 Jun 12;108:106951. doi: 10.1016/j.ultsonch.2024.106951 (PMC11227021; doi:10.1016/j.ultsonch.2024.106951)
Supplement: Supplementary Data 1 [file mmc1.docx]

**Green-friendly synthesis of 1,3,5-triazine derivatives using a sonochemical protocol**

Damian Kułaga^a^*, Anna K. Drabczyk^a^, Przemysław Zaręba^b^, Jolanta Jaśkowska^a^, Julia Chrzan^a^, Katarzyna Ewa Greber^c^, Krzesimir Ciura^c,d^, Damian Plażuk^e^ , Ewelina Wielgus^f^

^a^ Faculty of Chemical Engineering and Technology, Department of Organic Chemistry and Technology, Cracow University of Technology, 24 Warszawska Street, 31-155 Cracow, Poland

^b^Faculty of Chemical Engineering and Technology, Department of Chemical Technology and Environmental Analytics, Cracow University of Technology, 24 Warszawska Street, 31-155 Cracow, Poland

^c^ Department of Physical Chemistry, Medical University of Gdańsk, Al. Gen. Hallera 107, 80-416 Gdańsk, Poland

^d^ Laboratory of Environmental Chemoinformatics, Faculty of Chemistry, University of Gdansk, Wita Stwosza 63, 80-308 Gdansk, Poland

^e^ University of Lodz, Faculty of Chemistry, Department of Organic Chemistry, Laboratory of Molecular Spectroscopy, ul. Tamka 12, 91-403 Łódź, Poland

^f^Centre of Molecular and Macromolecular Studies, Polish Academy of Sciences, Sienkiewicza 112, 90-363 Lodz, Poland

**APPENDIX A**

* email: [damian.kulaga@pk.edu.pl](mailto:damian.kulaga@pk.edu.pl)

1. Comparison of microwave and sonochemical approaches…………………………………………….………2
2. Characterization of Intermediates………………………………………………………………………………….…..…6
3. UPLC-MS spectra for method optimization – synthesis of compound 3…………………………..……7
4. UPLC-MS spectra for crude **3a, 3b, 3d, 4a-4c, 5a, 5b** compounds……………………………………..…62
5. Spectra for final compounds **3**, **3a-3d** – **5a-5d** (UPLC-MS, HRMS, ^1^H NMR, ^13^C NMR)…….……67
6. Lipophilicity, drug-plasma proteins binding, and phospholipids affinity assays………….………119
7. References…………………………………………………………………………………………………………………………121

**Comparison of microwave and sonochemical approaches**

**Table S1.** Comparison of the influence of MW and ))) on the formation of products **3d**, **3e**, **3f** in the presence of K_2_CO_3_ and Na_2_CO_3_. For the MW protocol: yield (%) based on the yield of the product isolated from the reaction mixture; for the ))) protocol – yield (%) based on the UPLC-MS analysis of the post reaction mixture only

|  |  | MICROWAVE IRRADIATION* | | SONOCHEMISTRY  by UPLC-MS | |
| --- | --- | --- | --- | --- | --- |
| Cmpd No | structure | with K_2_CO_3_ | with Na_2_CO_3_ | with K_2_CO_3_ | with Na_2_CO_3_ |
| 3d |  | - | 81 % | 33 %  m/z = 407  [M+H] | 91 %  m/z = 407  [M+H] |
| 3e |  | - | 60 % | 30 %  m/z = 419  [M+H] | 49 %  m/z = 419  [M+H] |
| 3f |  | - | 70 % | 45 %  m/z = 423  [M+H] | 78 %  m/z = 423  [M+H] |

* according to [1]; - no product formed


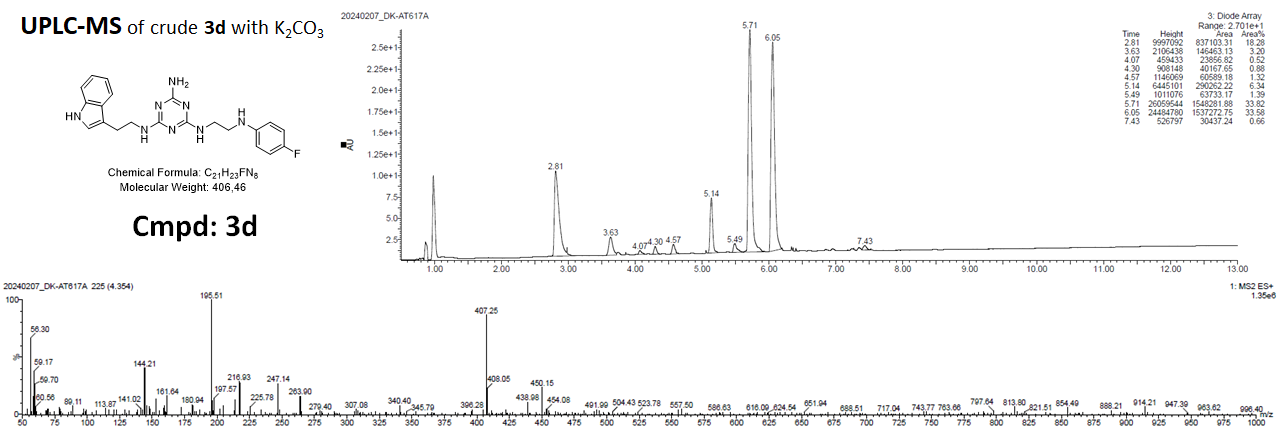


LC-MS according to method B

LC-MS according to method B

LC-MS according to method B


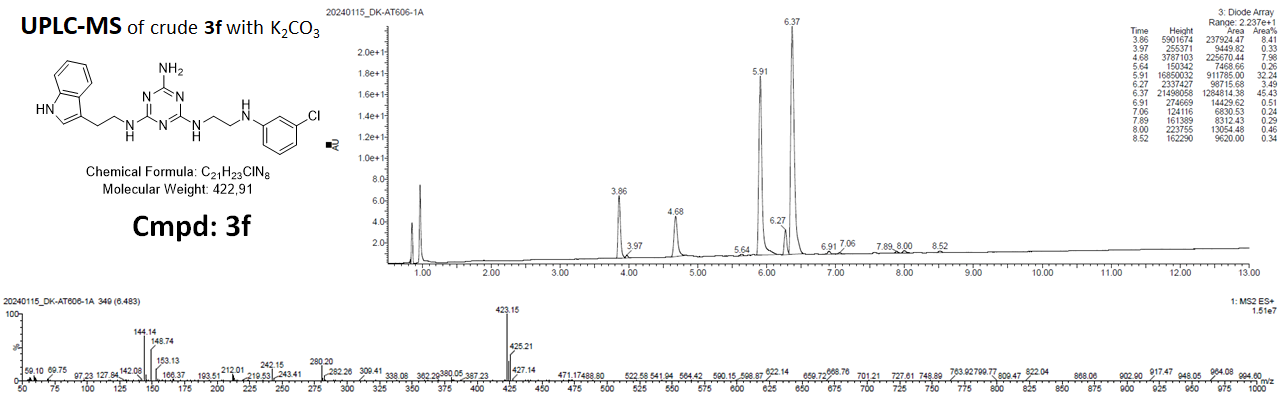


LC-MS according to method B

LC-MS according to method B

**Characterization of Intermediates**

*4,6-dichloro-N-phenyl-1,3,5-triazin-2-amine (****9****)*

White solid, 86 % yield,

*4,6-dichloro-N-(3-chlorophenyl)-1,3,5-triazin-2-amine (****10****)*

White solid, 81 % yield, ^1^H NMR (400 MHz, DMSO) δ 11.02 (s, 1H, NH), 7.58 (dd, J = 7.8, 1.5 Hz, 1H, Ar-H), 7.52 (dd, J = 7.8, 1.6 Hz, 1H, Ar-H), 7.42 (td, J = 7.6, 1.6 Hz, 1H, Ar-H), 7.36 (td, J = 7.6, 1.7 Hz, 1H, Ar-H)

*N-benzyl-4,6-dichloro-1,3,5-triazin-2-amine (****14****)*

White solid, 73% yield; UPLC-MS analysis: t = 7.62 min, purity 90%, calc. for C_10_H_9_Cl_2_N_4_ *m/z* = 255.1 found *m/z* = 255.13

*6-chloro-N^2^-phenyl-1,3,5-triazine-2,4-diamine (****11****)*

White solid, 90% yield, UPLC-MS analysis: t = 4.67 min, purity 97%, calc. for C_9_H_9_ClN_5_ *m/z* = 222,05 found *m/z* = 222,1 [M+H]^+^

*6-chloro-N^2^-(3-chlorophenyl)-1,3,5-triazine-2,4-diamine (****12****)*

White solid, 88% yield, UPLC-MS analysis: t = 5.66 min, purity 100%, calc. for C_9_H_8_Cl_2_N_5_ *m/z* = 256.02 found *m/z* = 256.06

*N-benzyl-4-chloro-6-morpholino-1,3,5-triazin-2-amine (****16****)*

White solid, 56% yield, UPLC-MS analysis: t = 7.35 min, purity 97%, calc. for C_14_H_16_ClN_5_O *m/z* = 306.11 found *m/z* = 306.15 [M+H]^+^

**UPLC-MS spectra for method optimization – synthesis of compound 3**


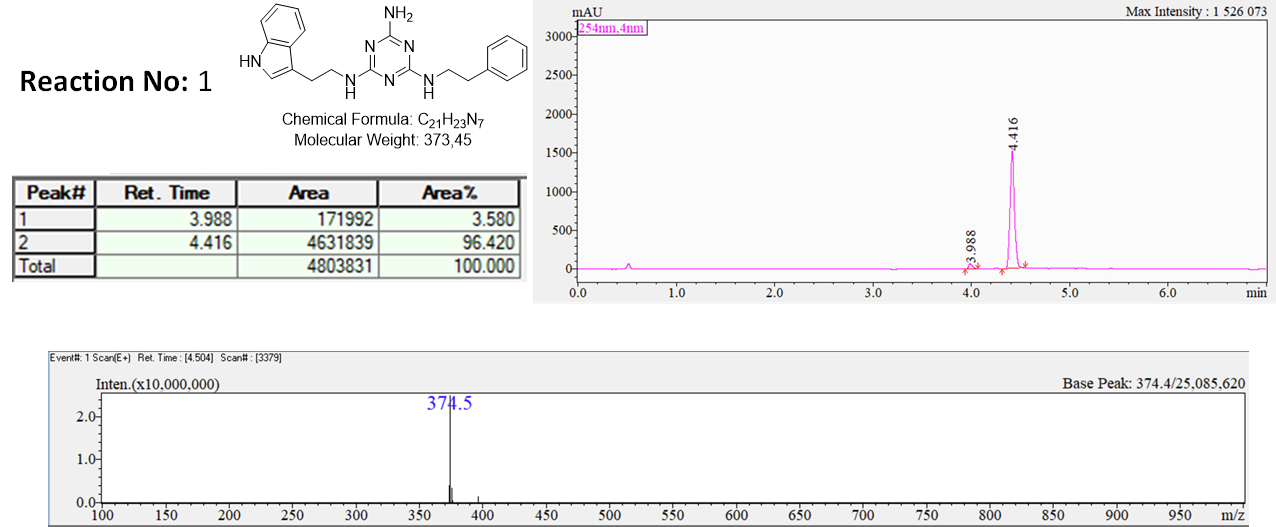


LC-MS according to method A

LC-MS according to method A


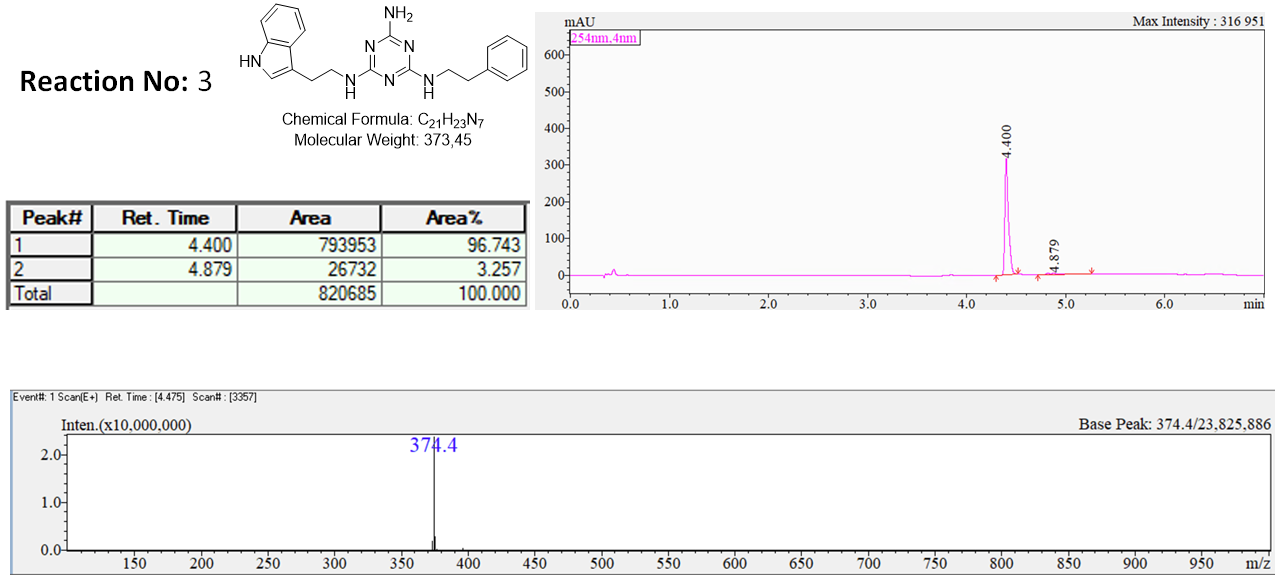


LC-MS according to method A


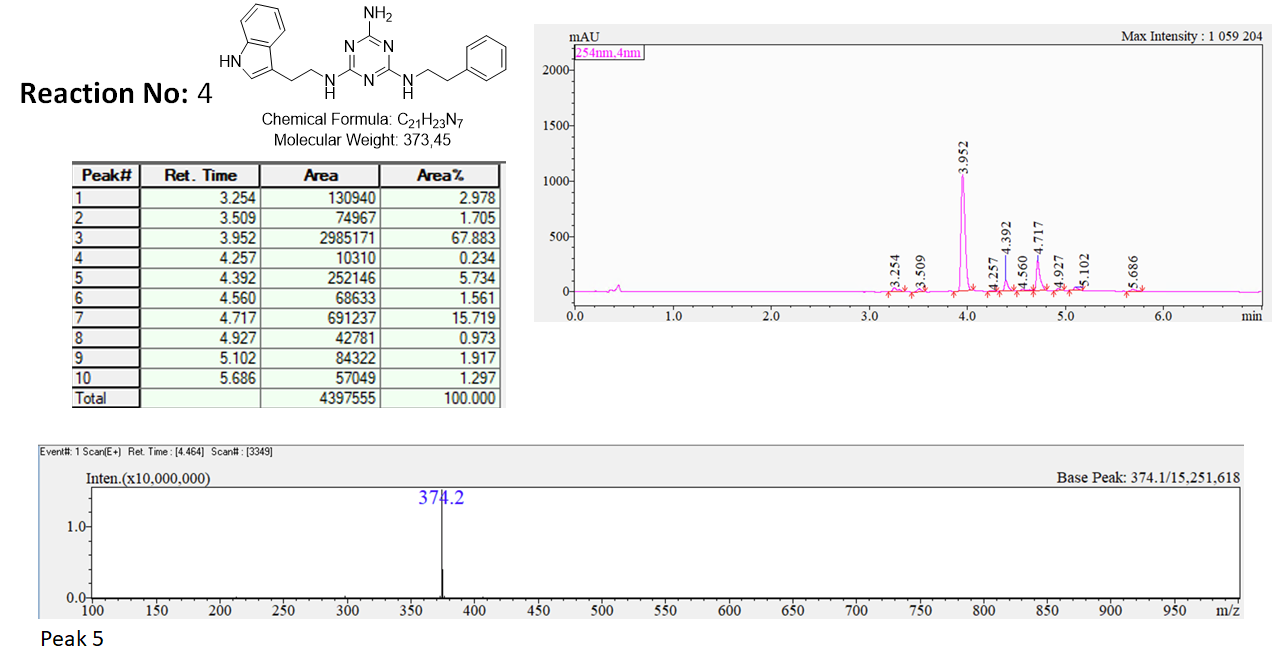


LC-MS according to method A


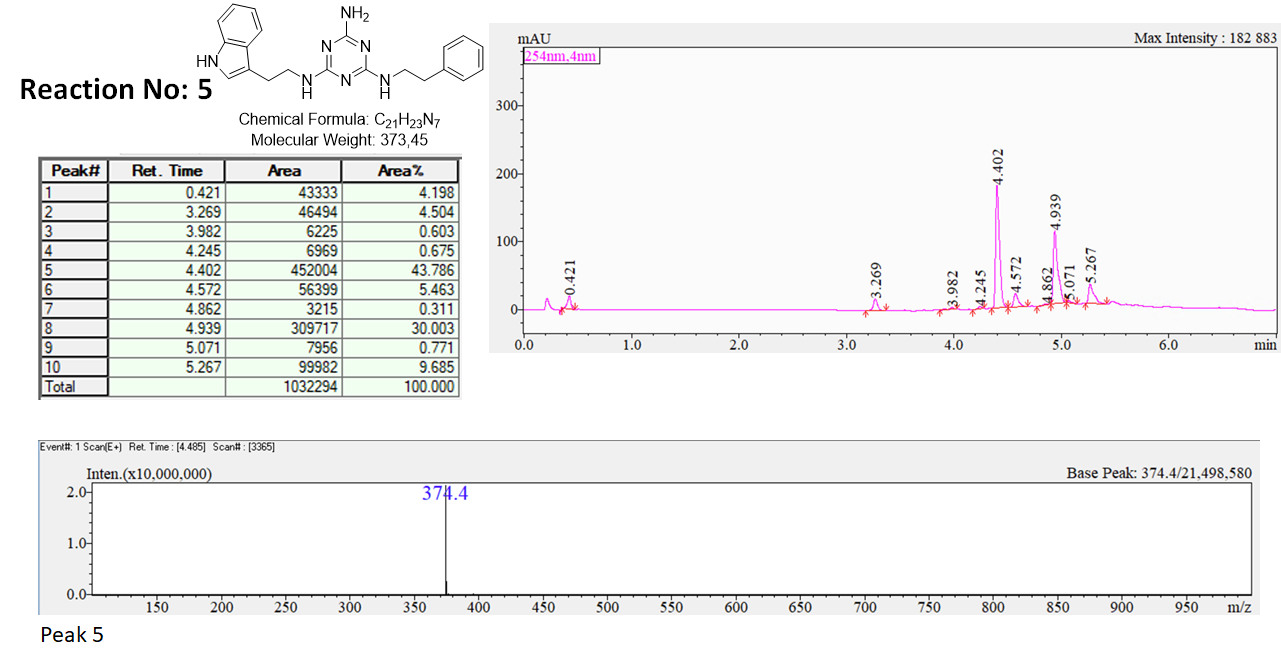


LC-MS according to method A


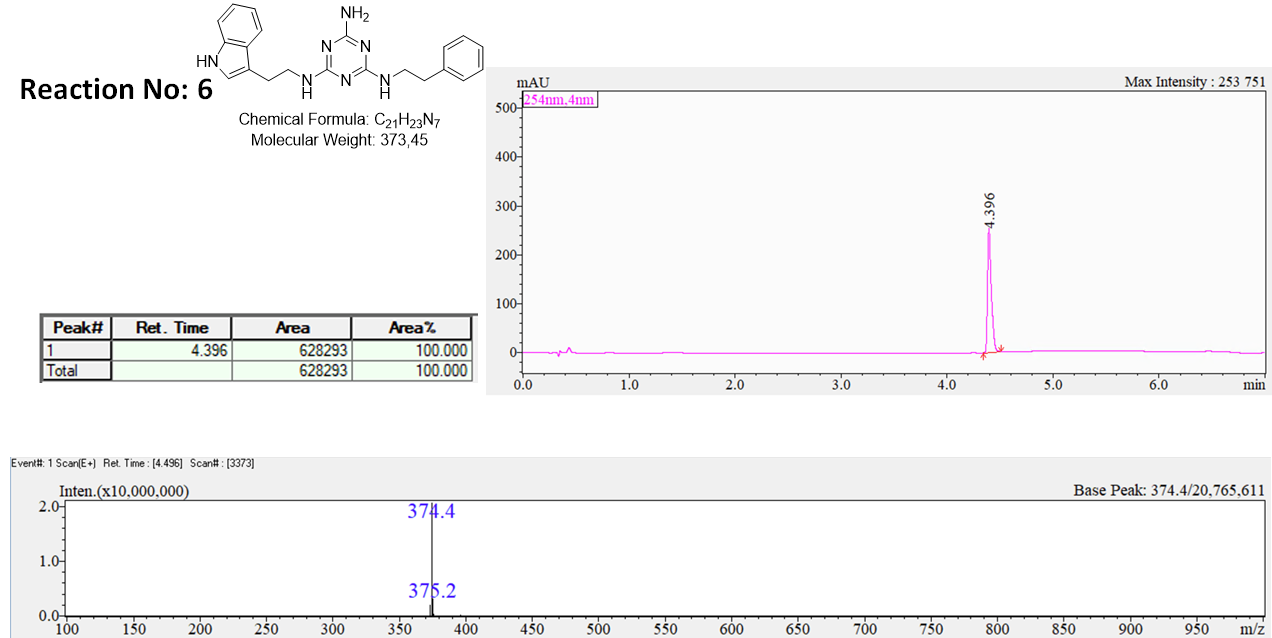


LC-MS according to method A


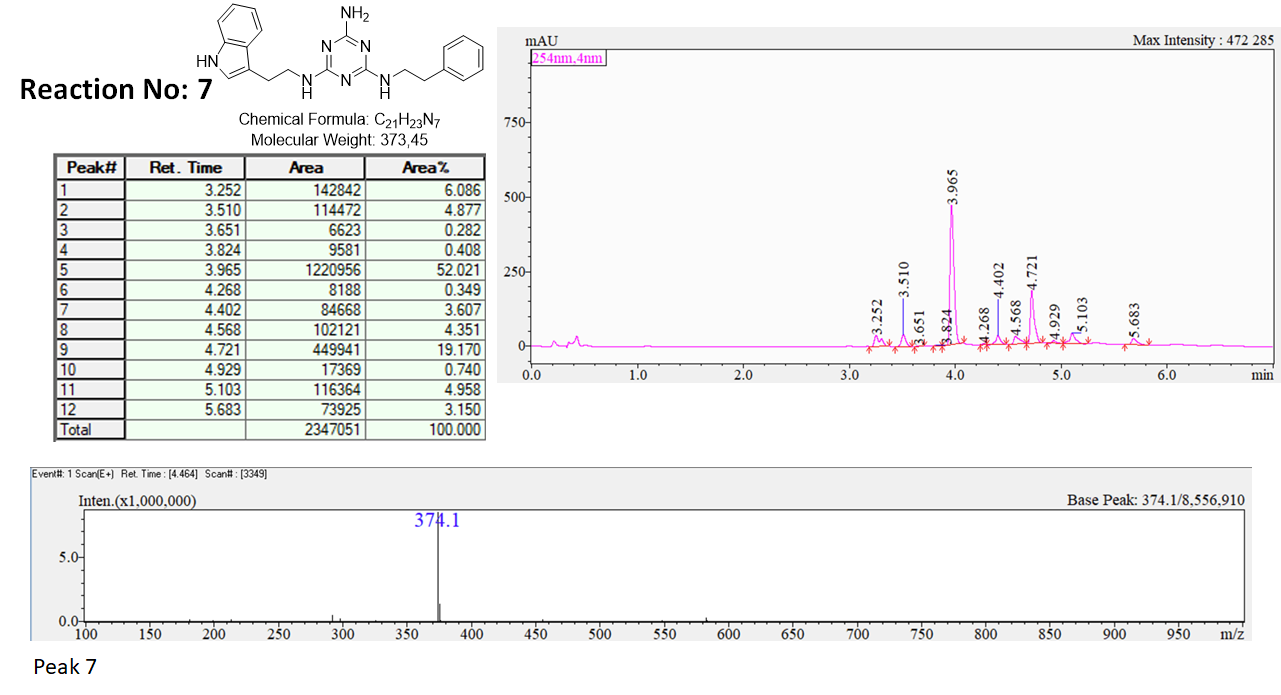


LC-MS according to method A


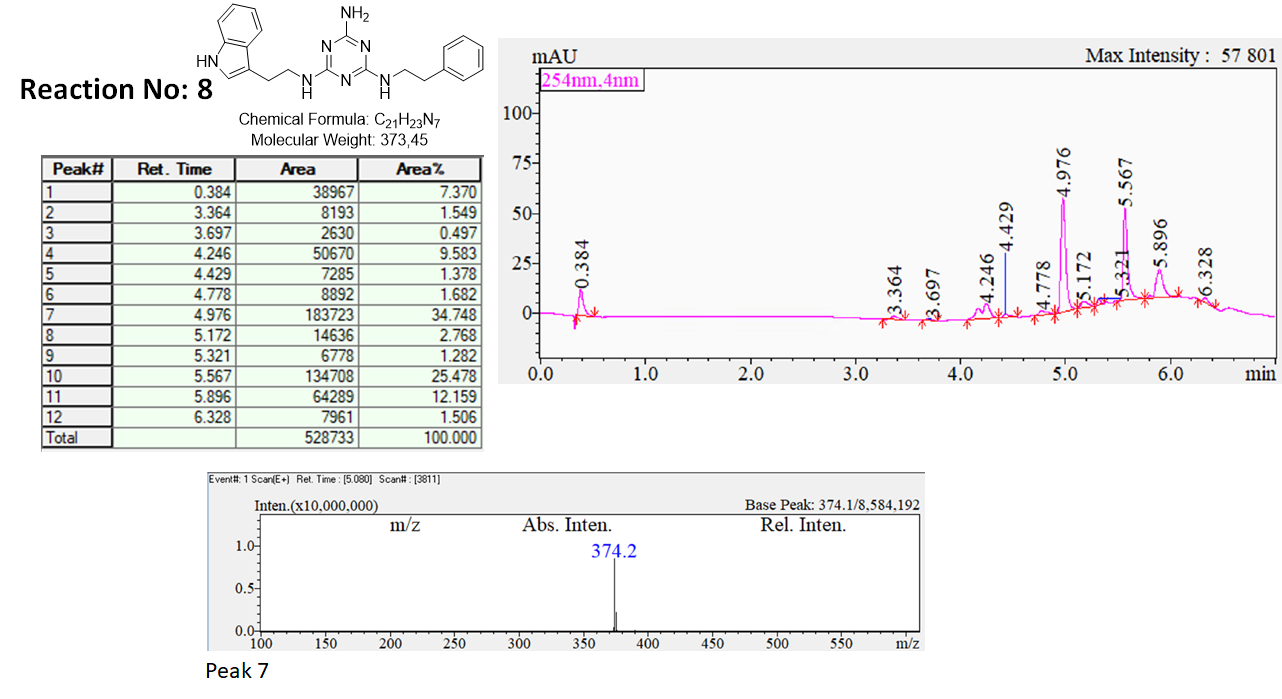


LC-MS according to method A


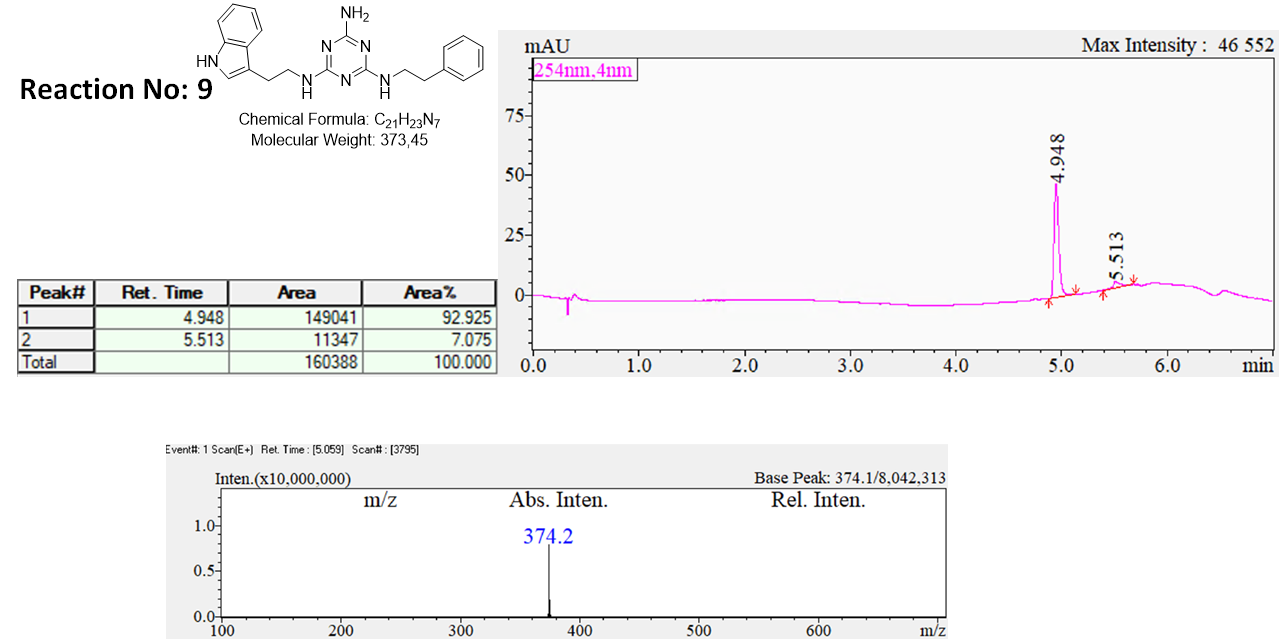


LC-MS according to method A

LC-MS according to method A


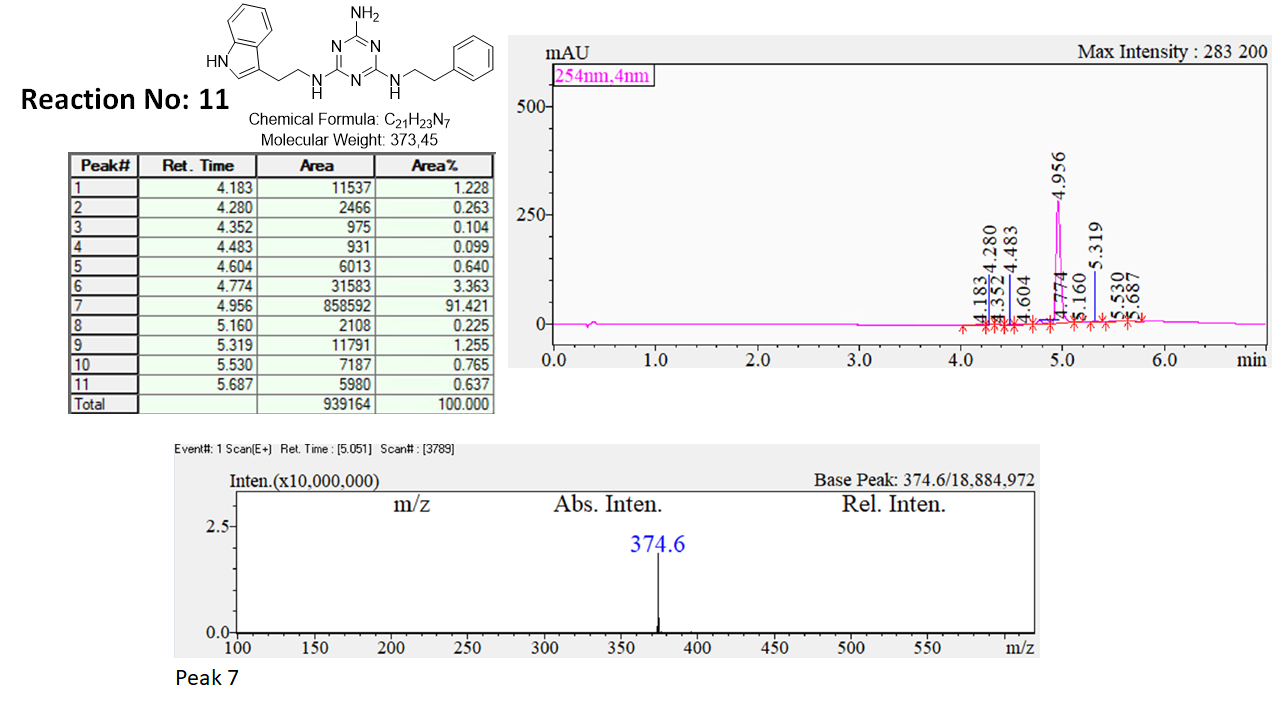


LC-MS according to method A


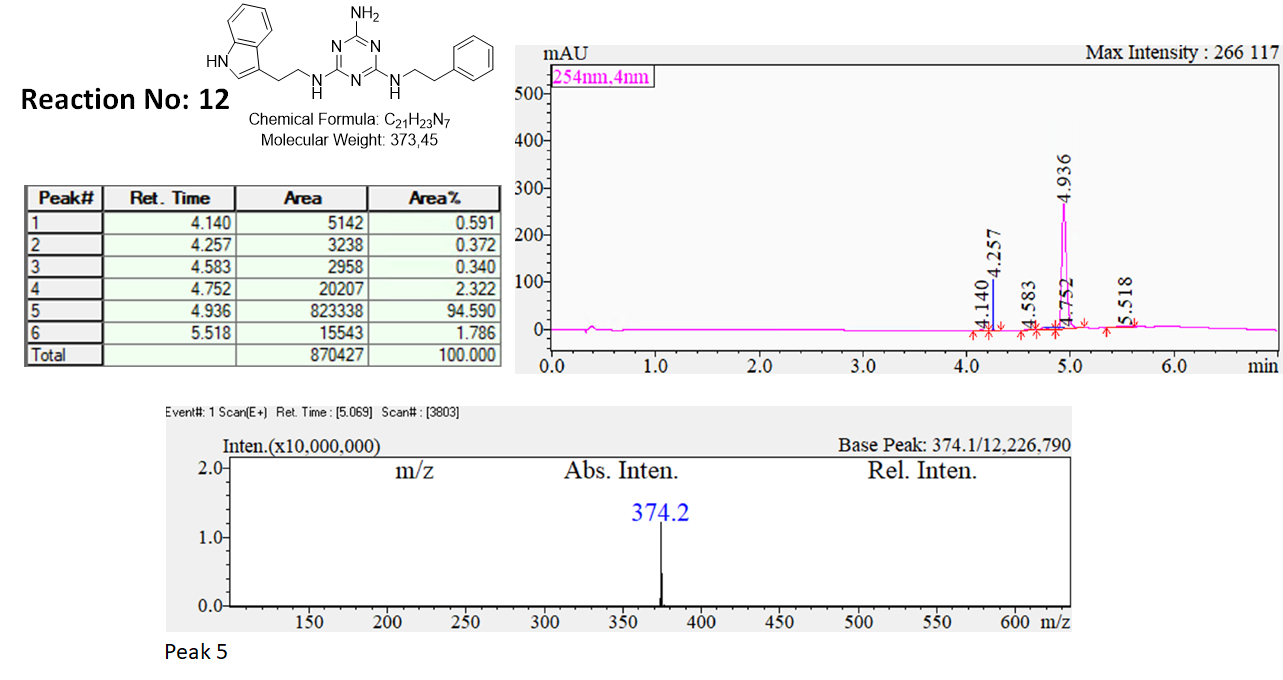


LC-MS according to method A


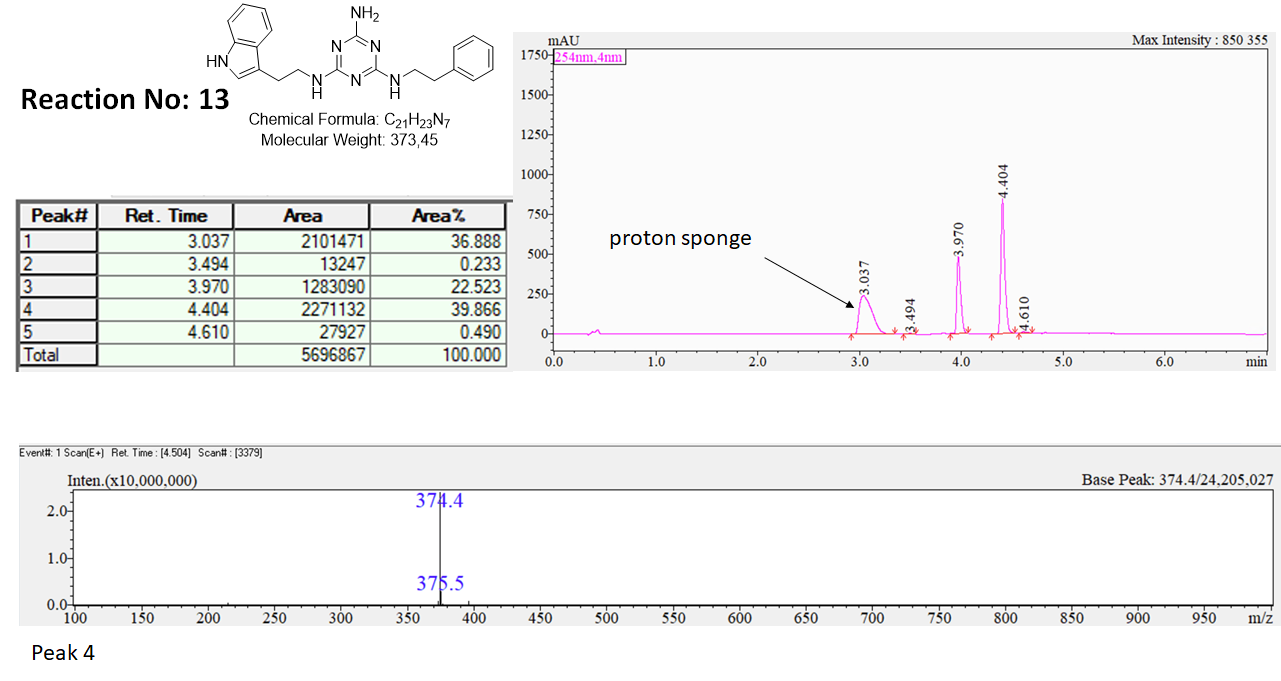


LC-MS according to method A


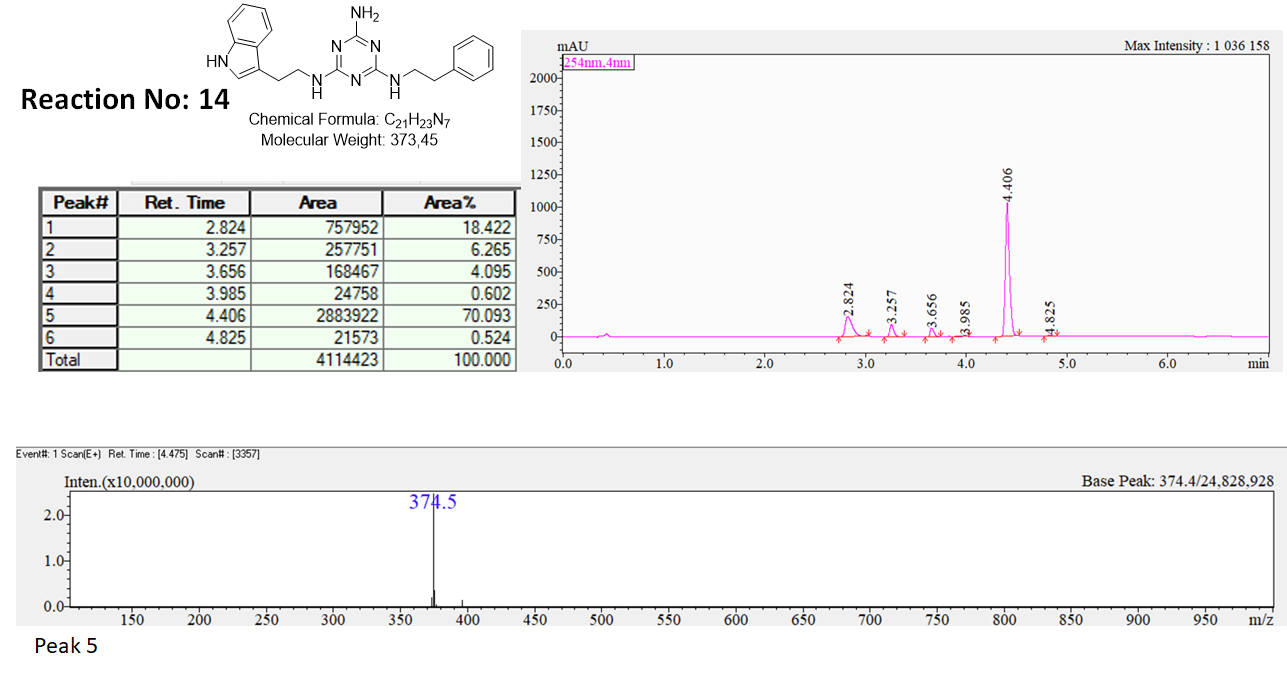


LC-MS according to method A


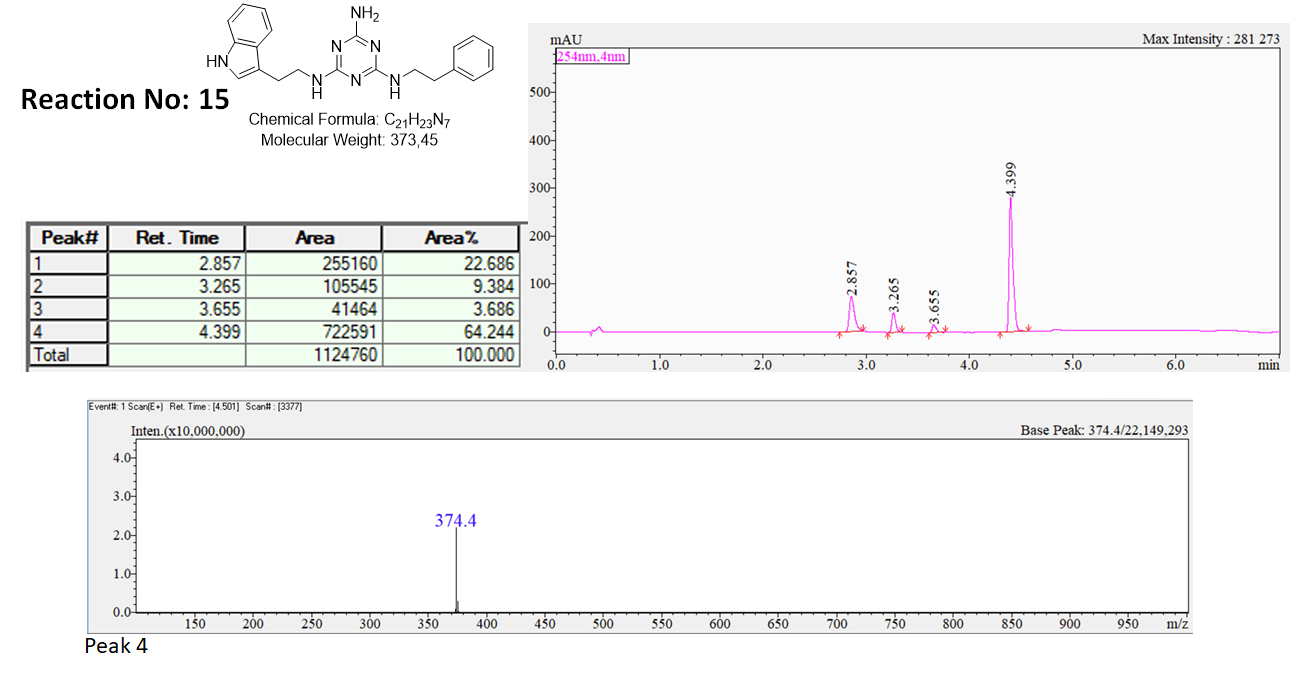


LC-MS according to method A


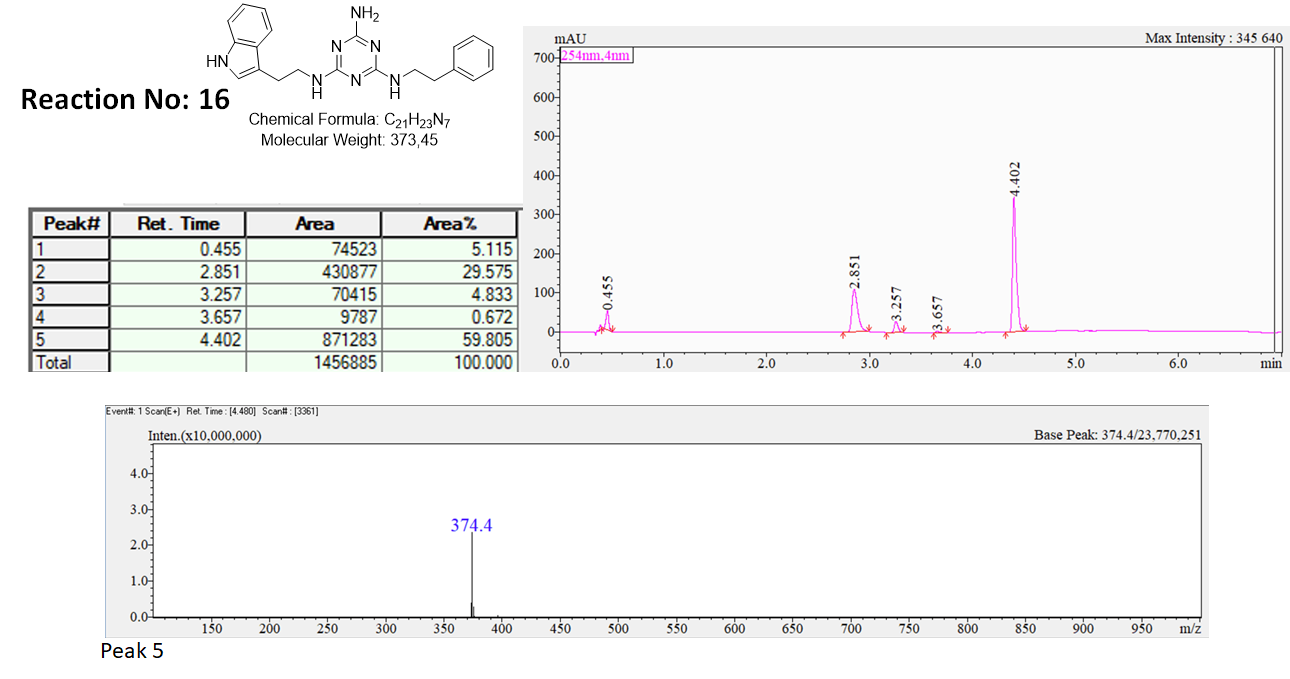


LC-MS according to method A


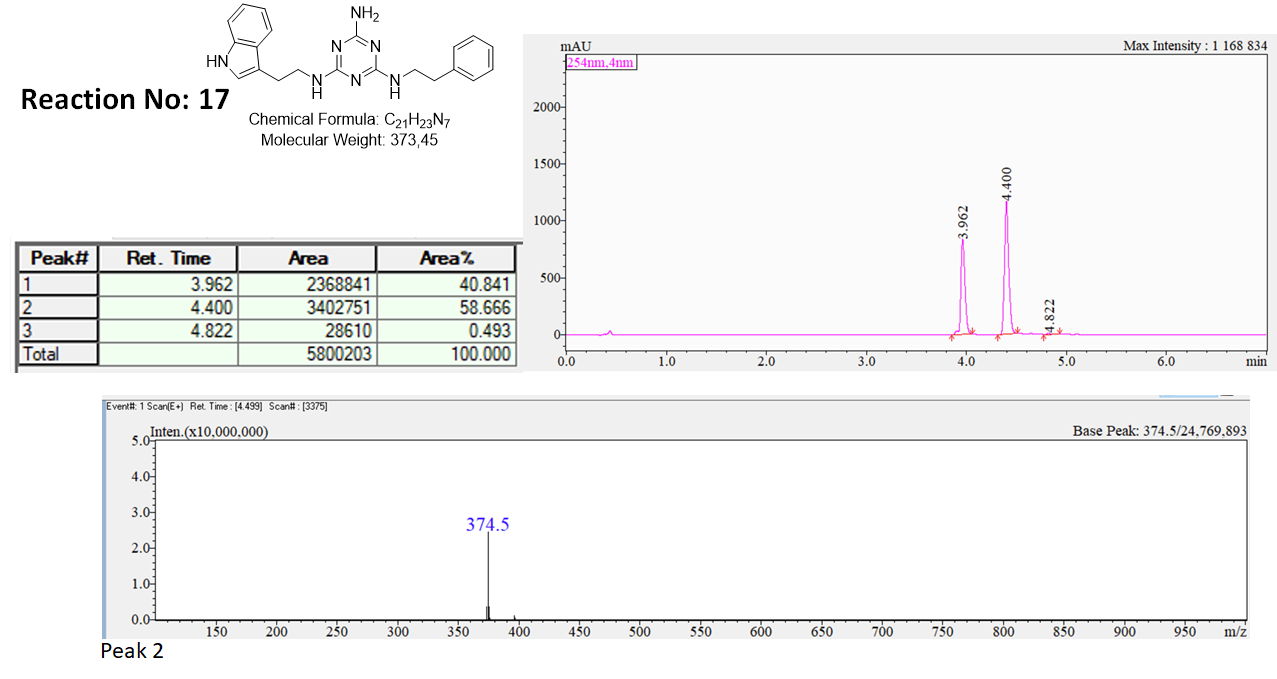


LC-MS according to method A


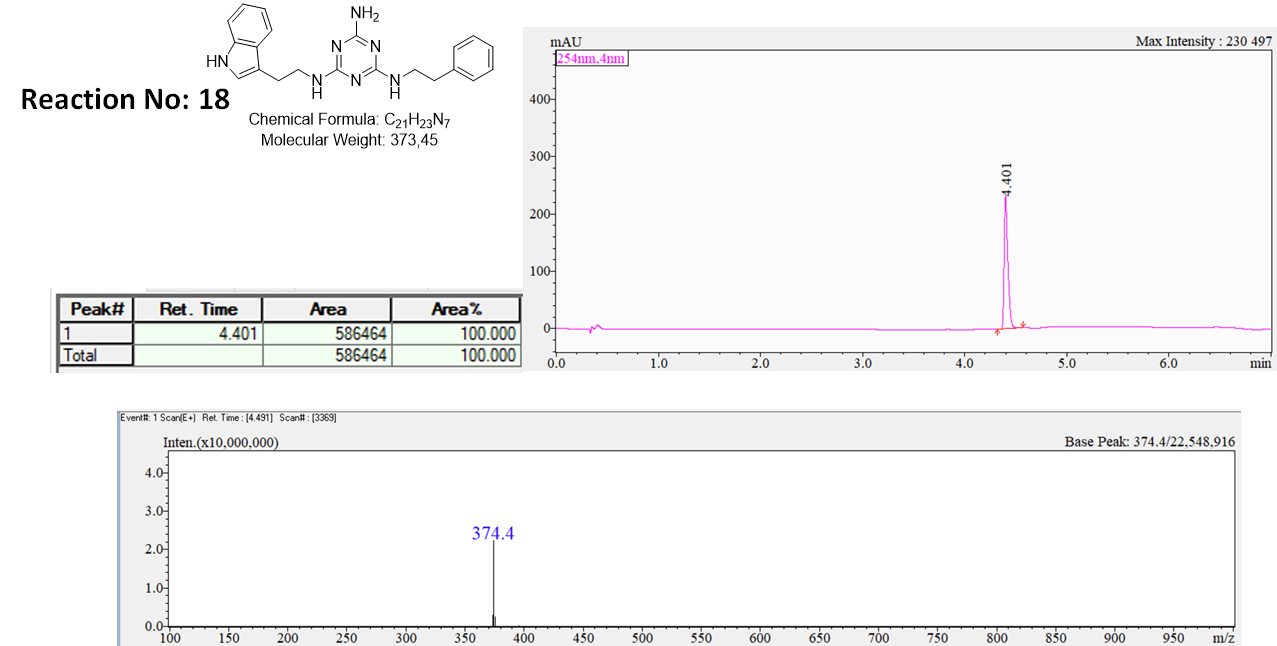


LC-MS according to method A


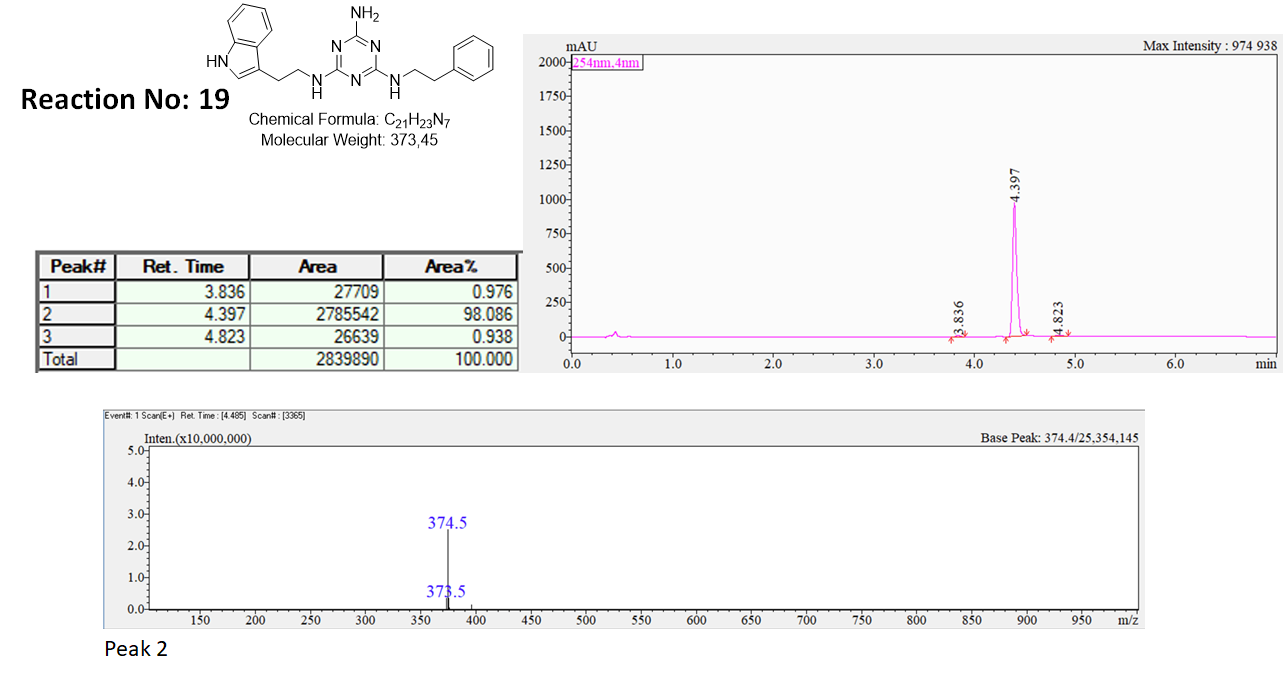


LC-MS according to method A


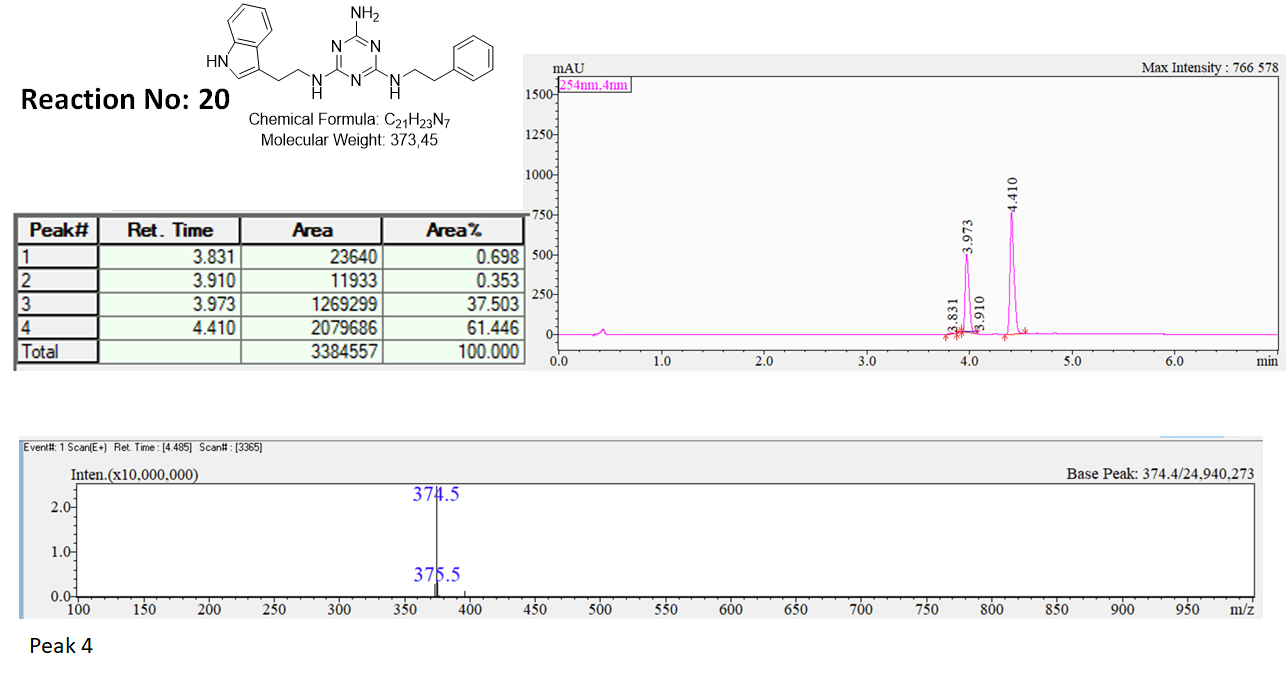


LC-MS according to method A


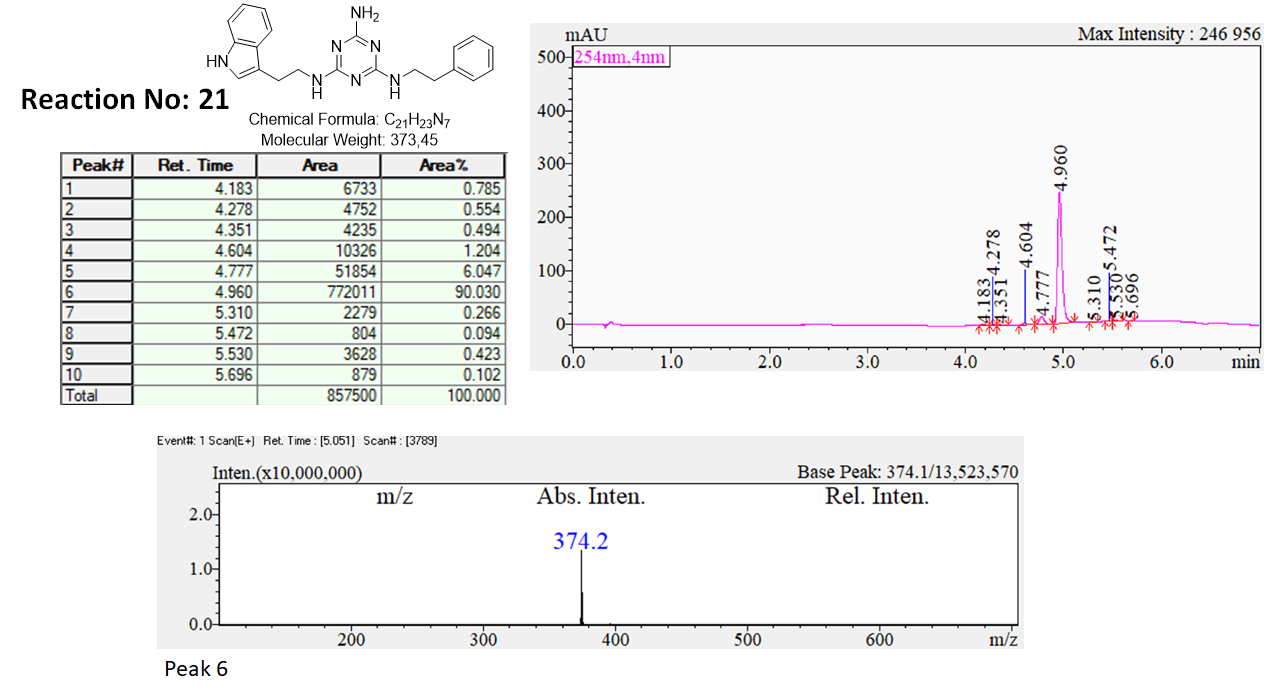


LC-MS according to method A


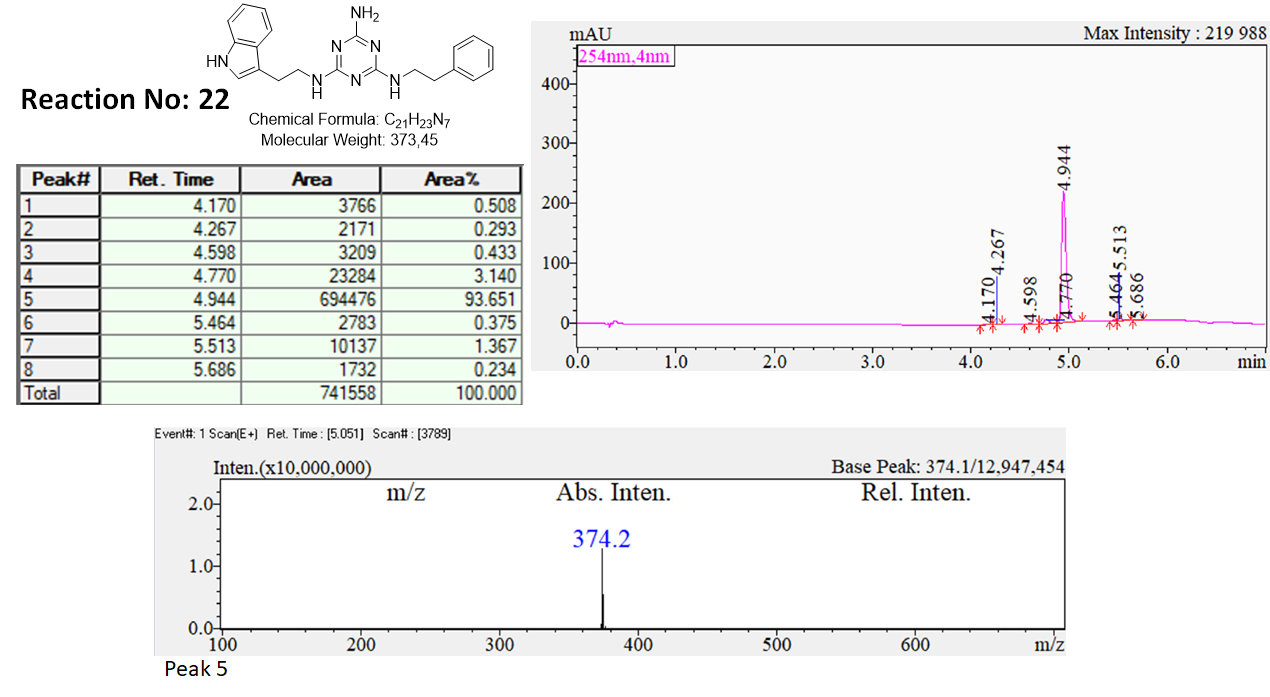


LC-MS according to method A


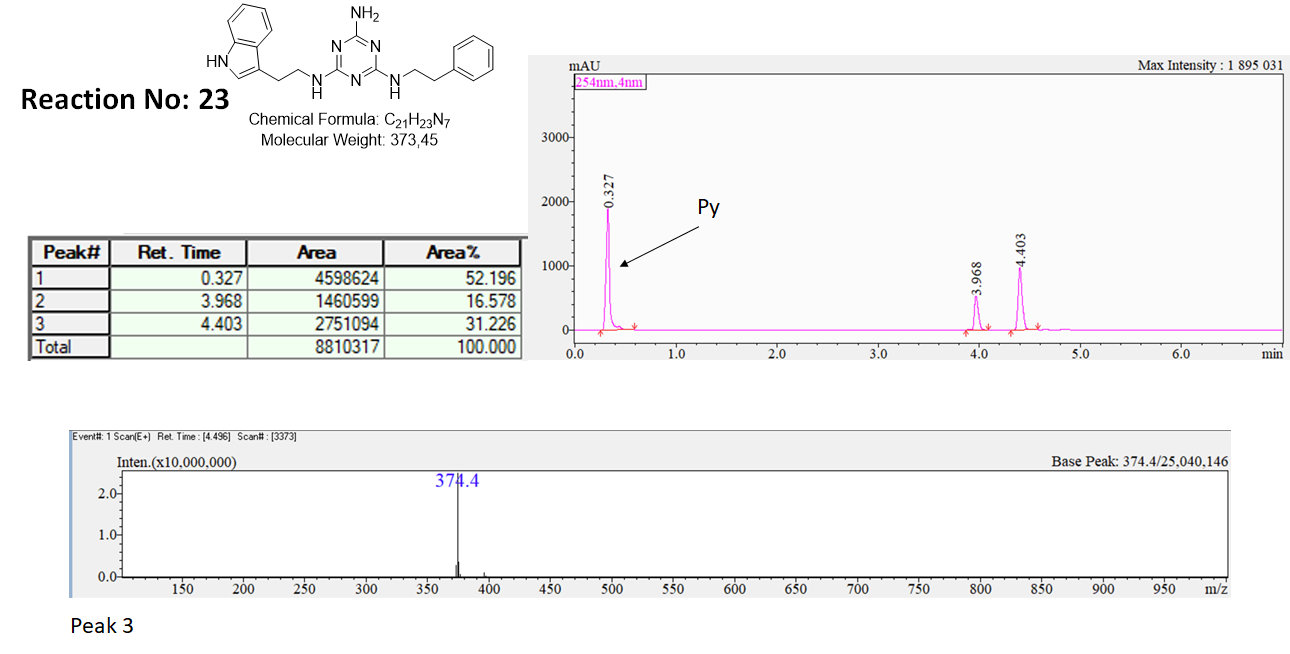


LC-MS according to method A


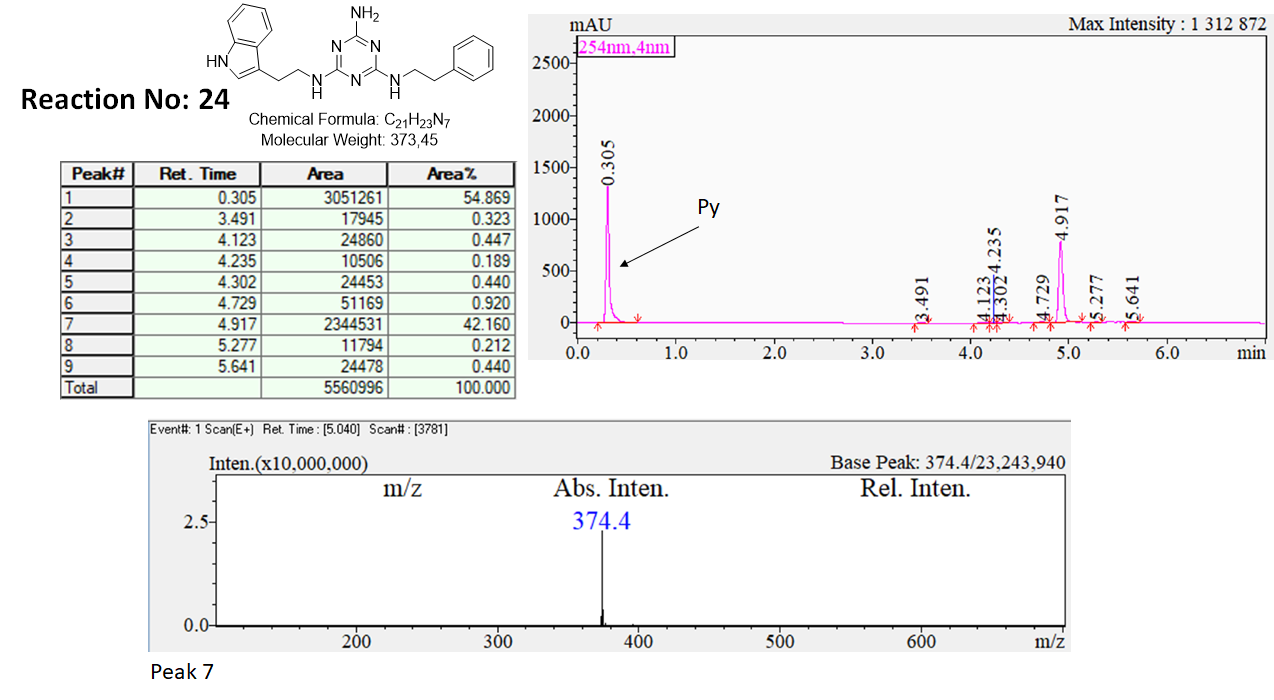


LC-MS according to method A


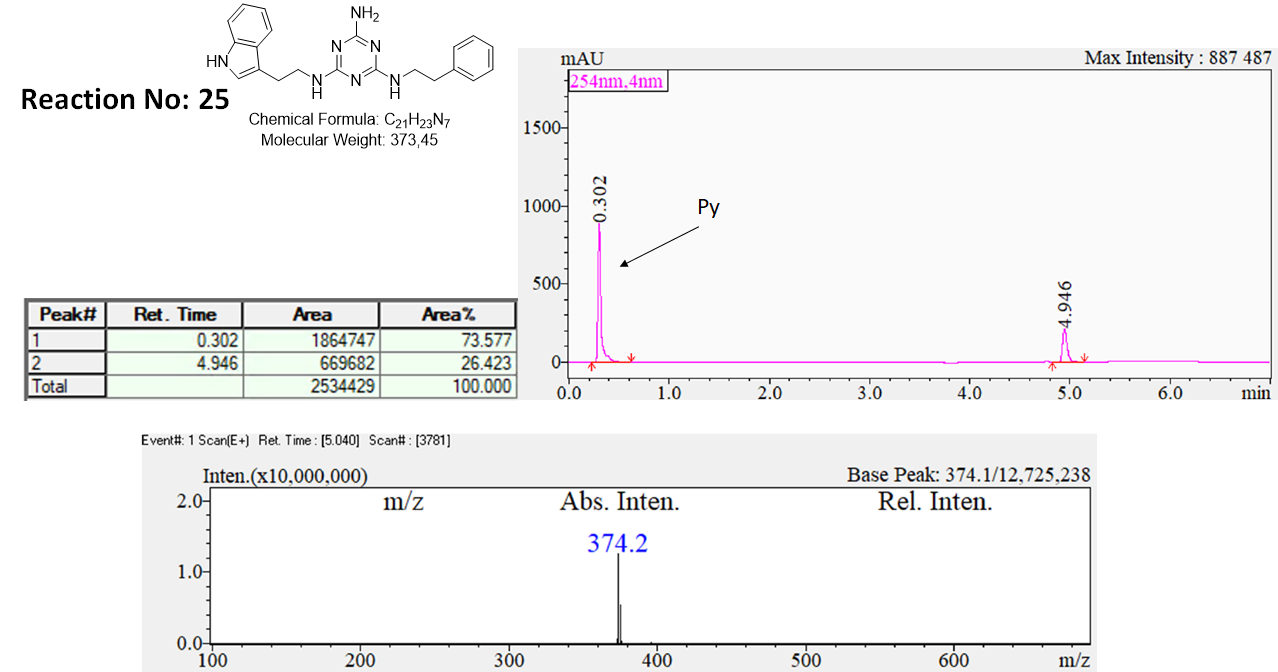


LC-MS according to method A

LC-MS according to method A

LC-MS according to method A


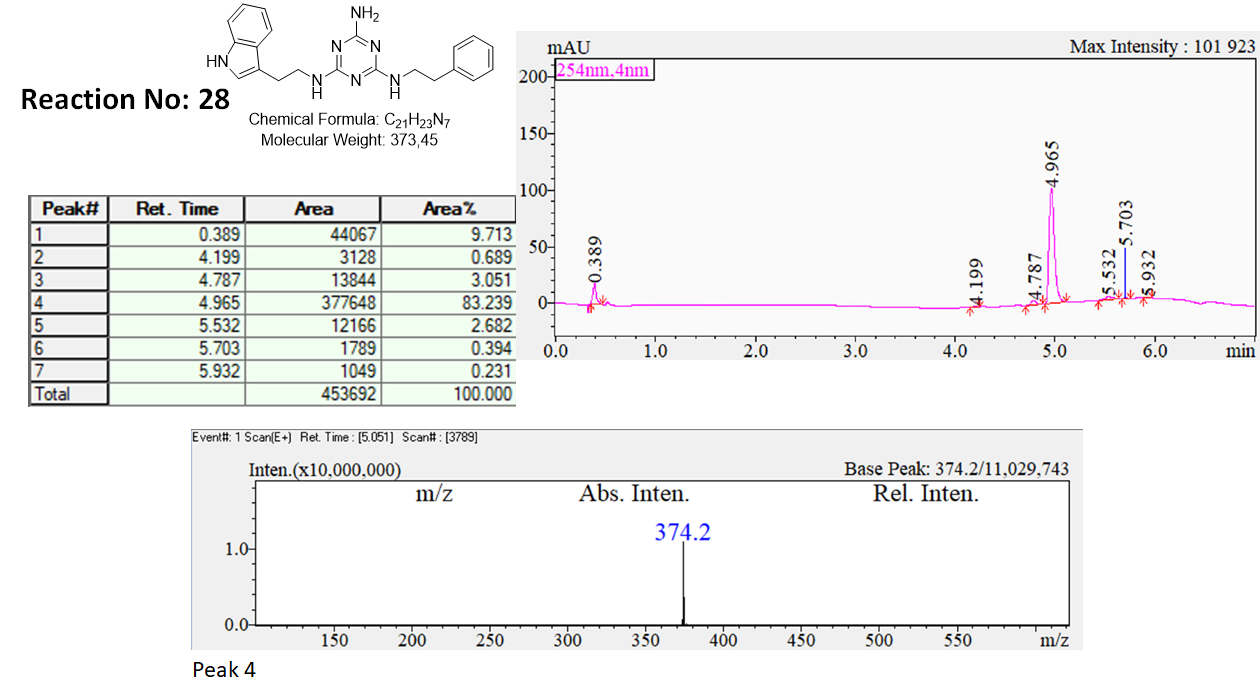


LC-MS according to method A


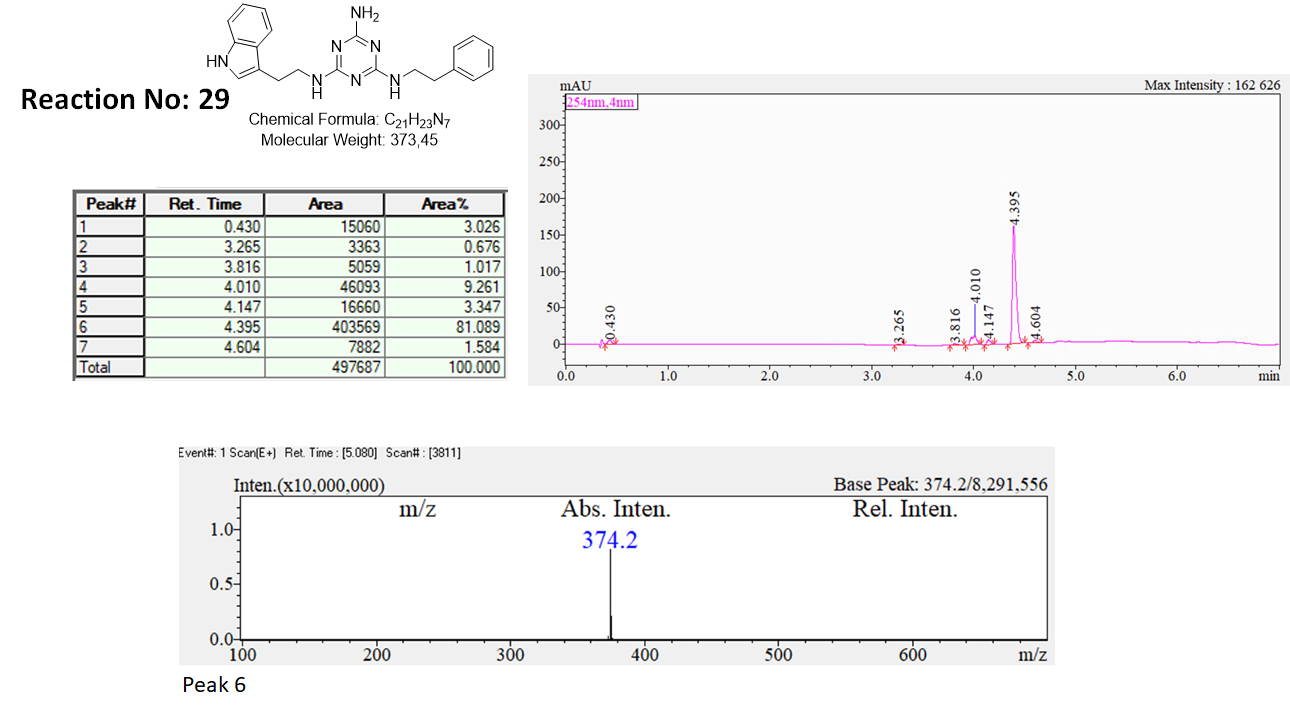


LC-MS according to method A


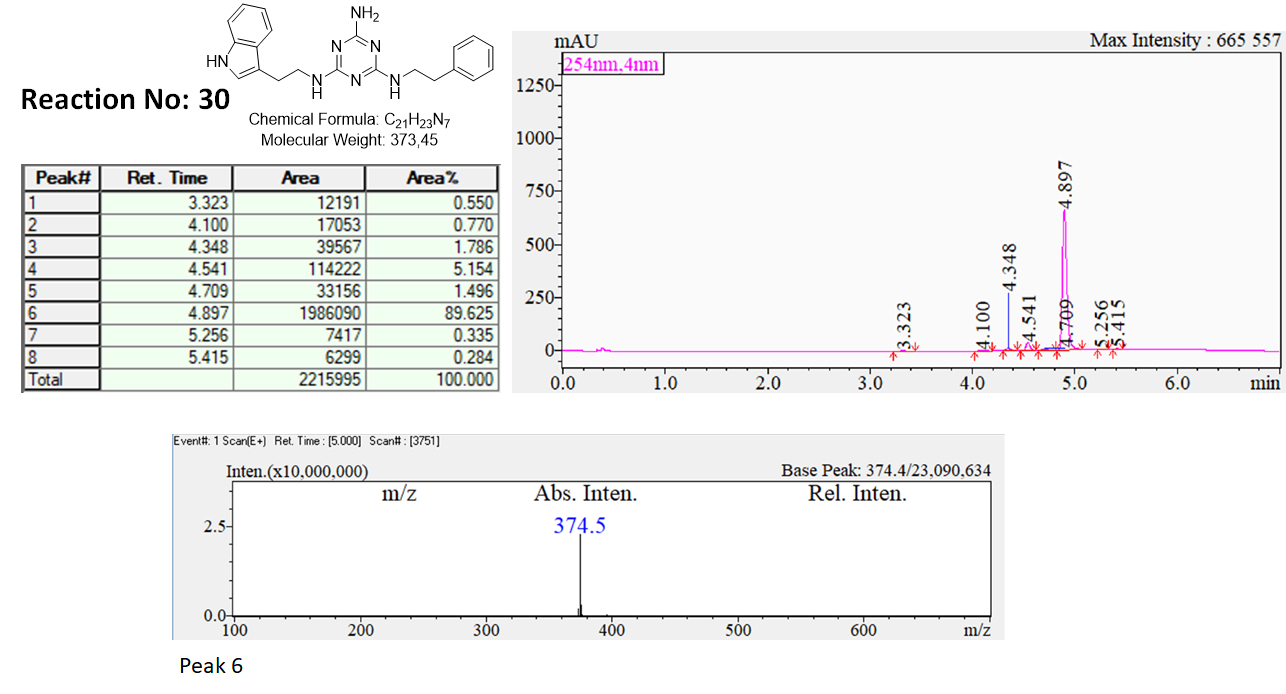


LC-MS according to method A


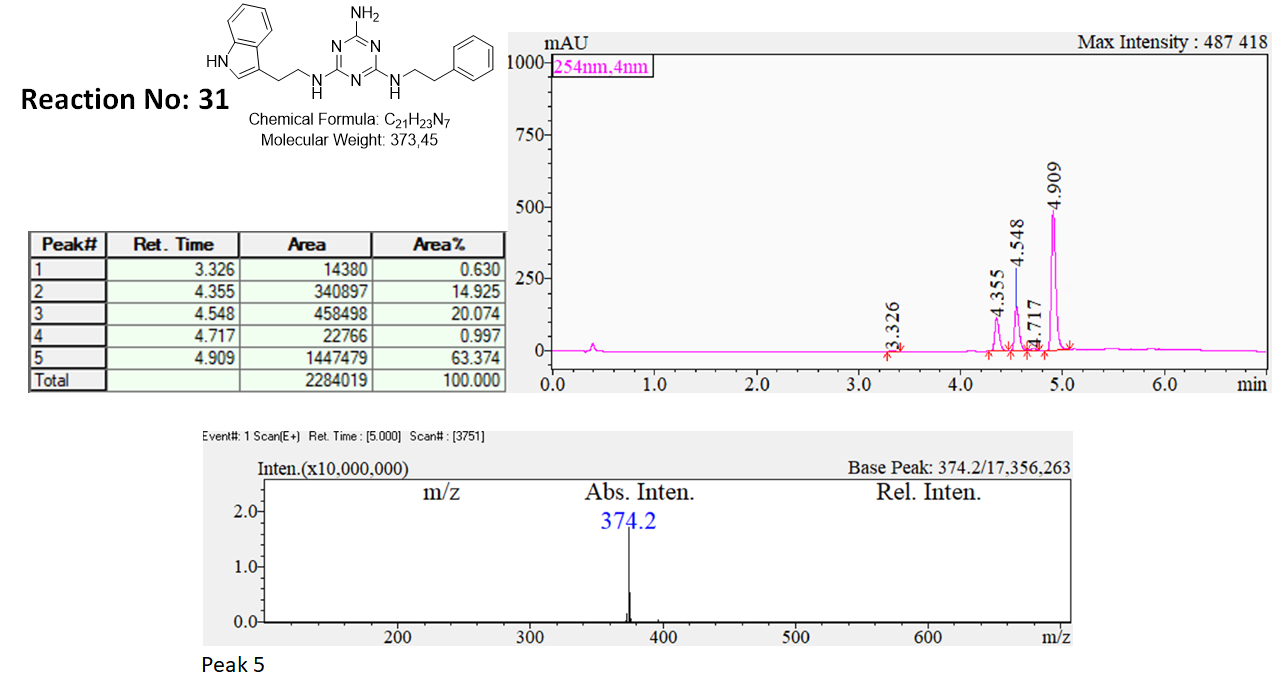


LC-MS according to method A


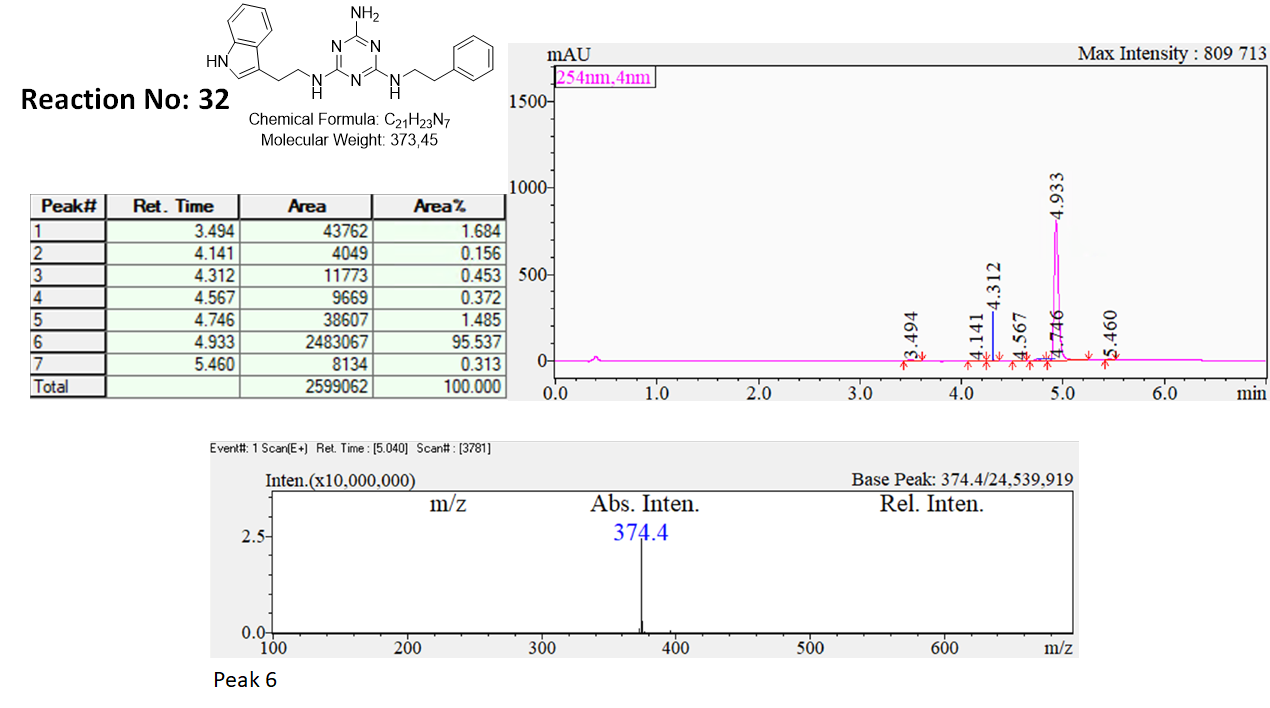


LC-MS according to method A


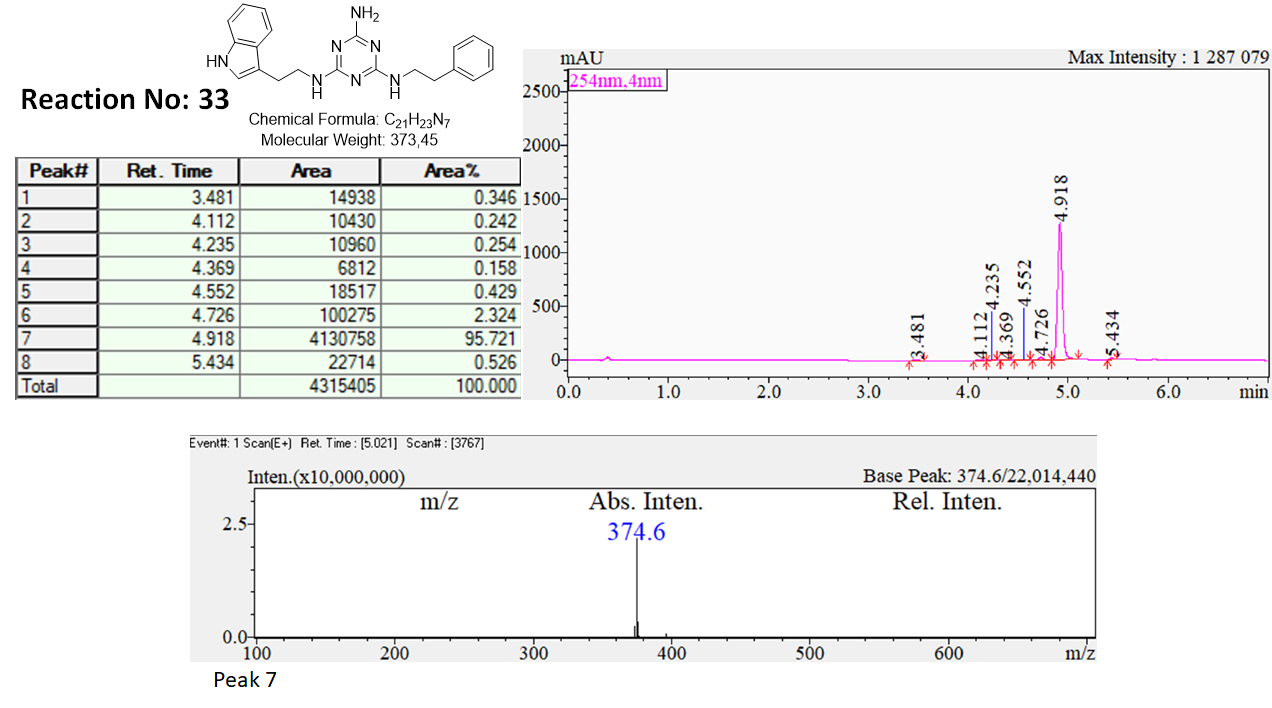


LC-MS according to method A


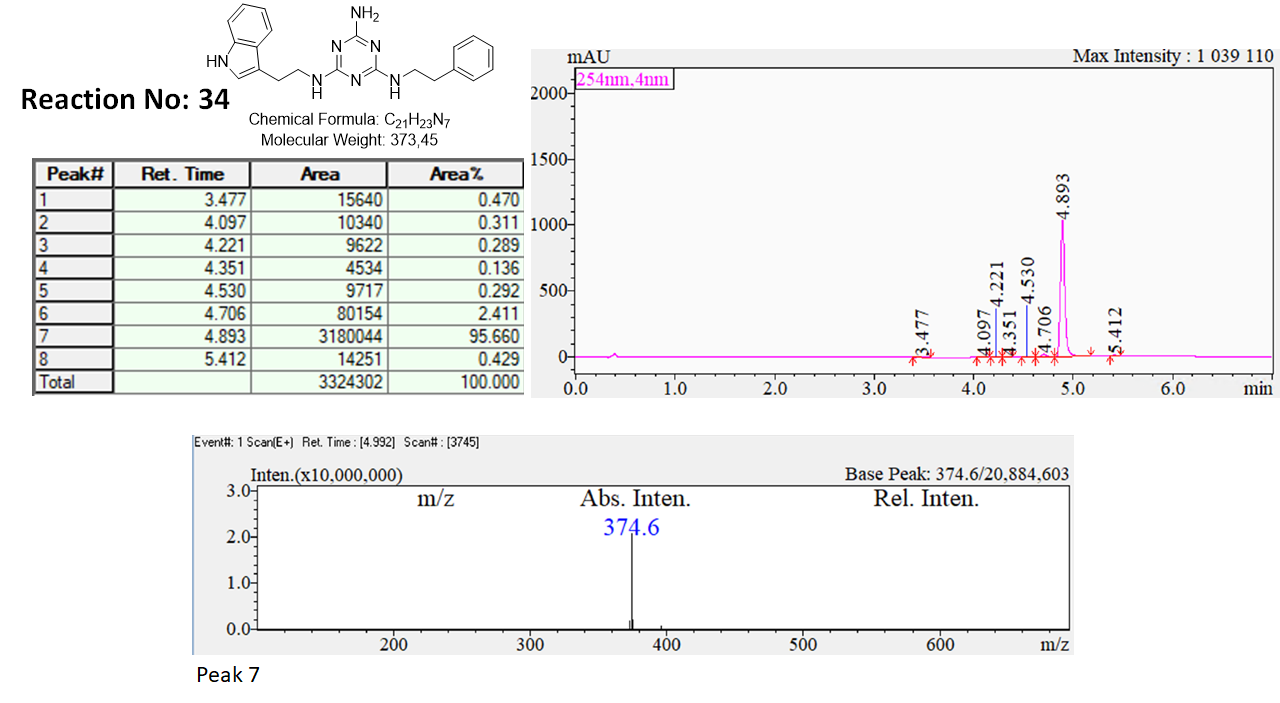


LC-MS according to method A


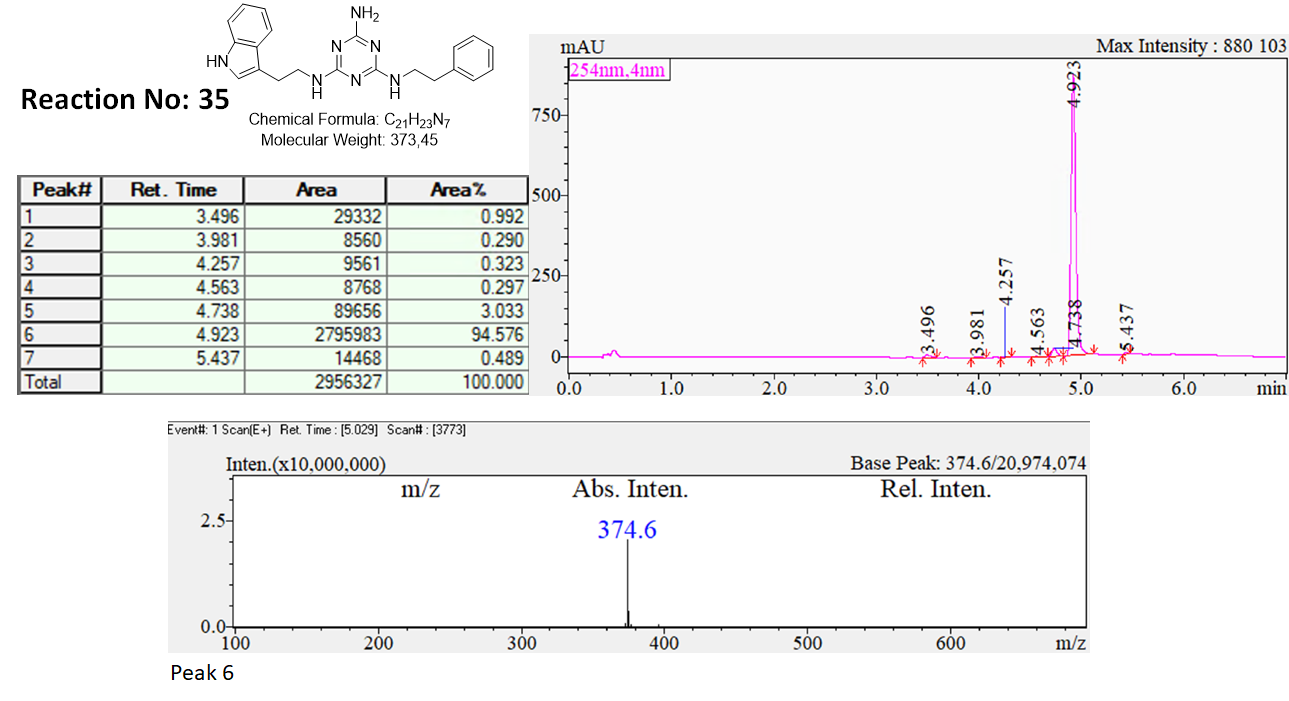


LC-MS according to method A


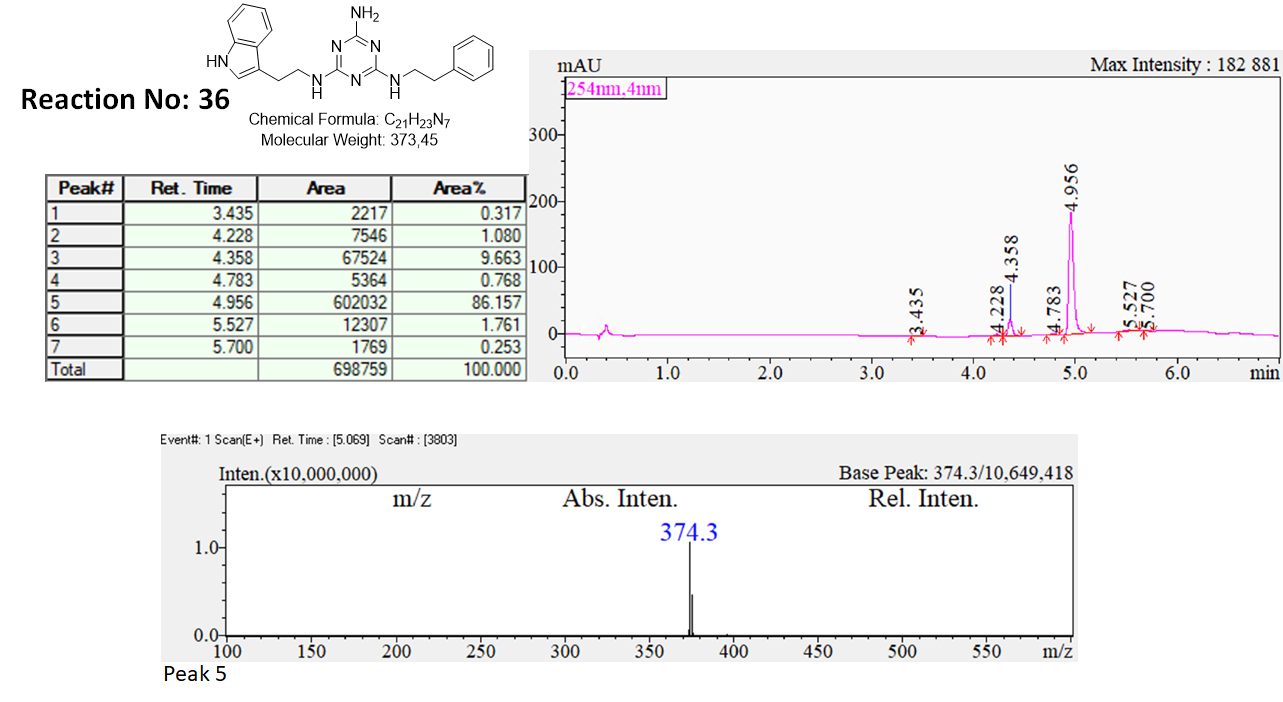


LC-MS according to method A


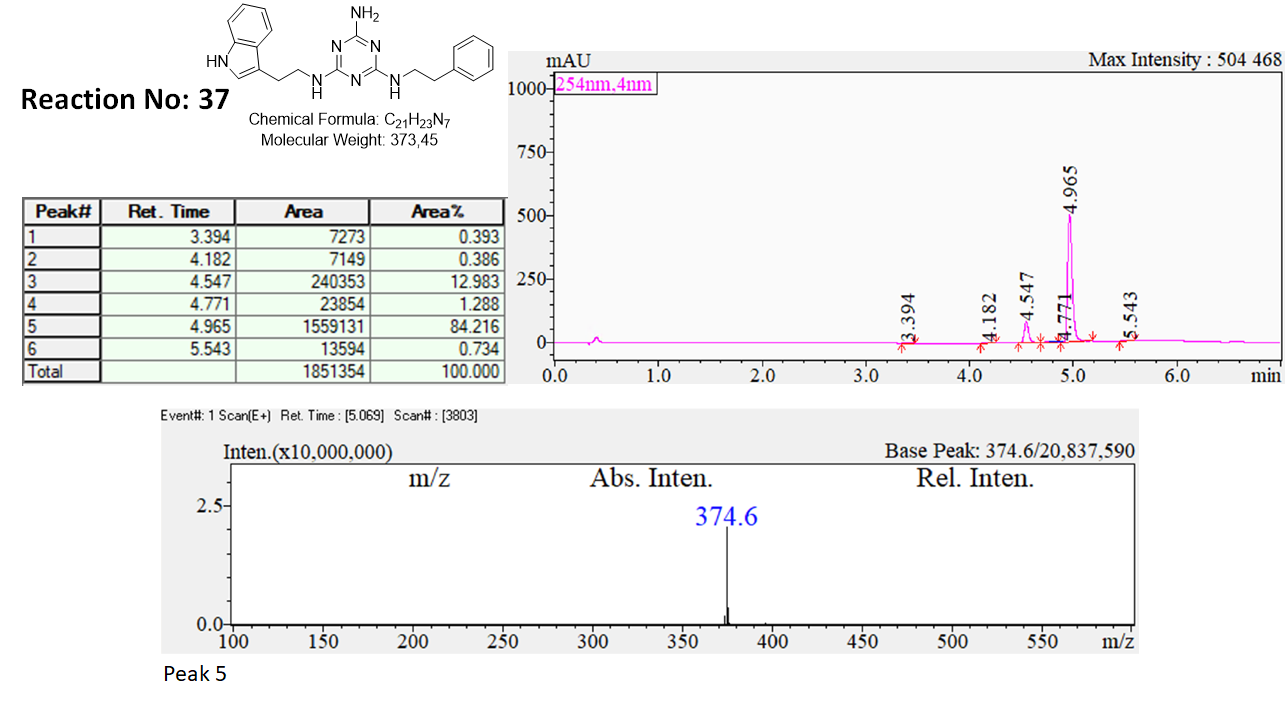


LC-MS according to method A


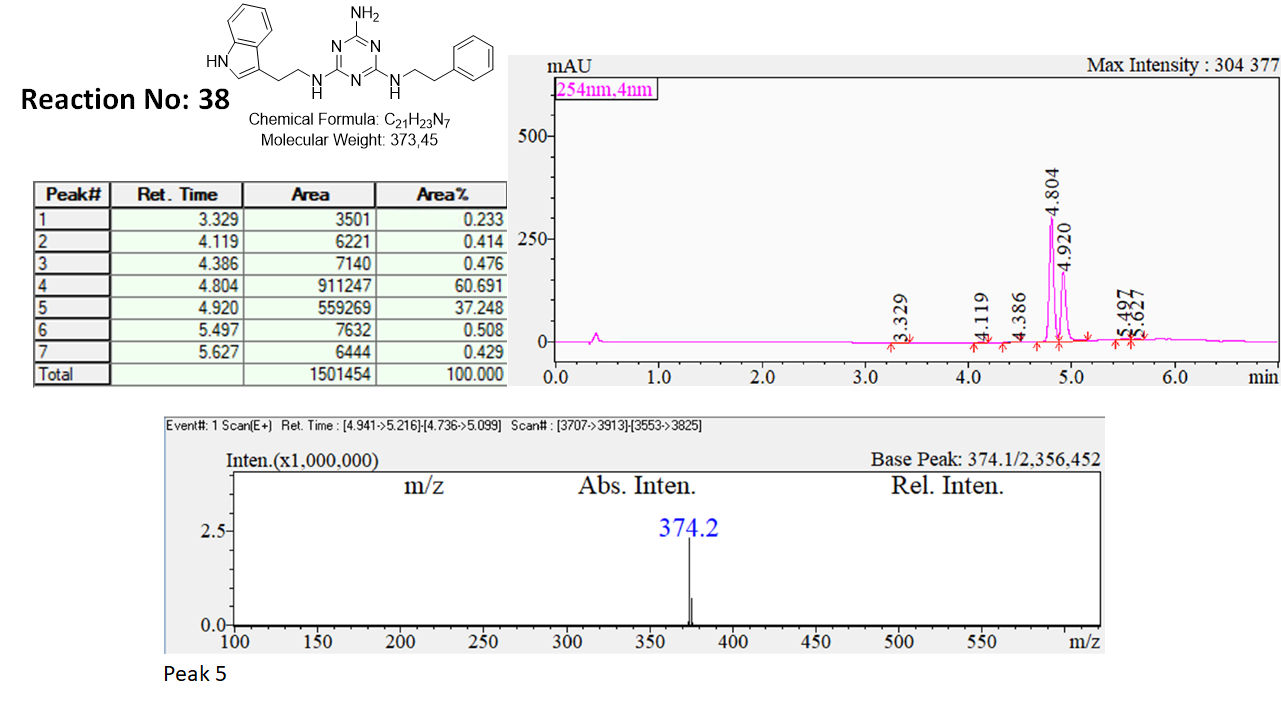


LC-MS according to method A


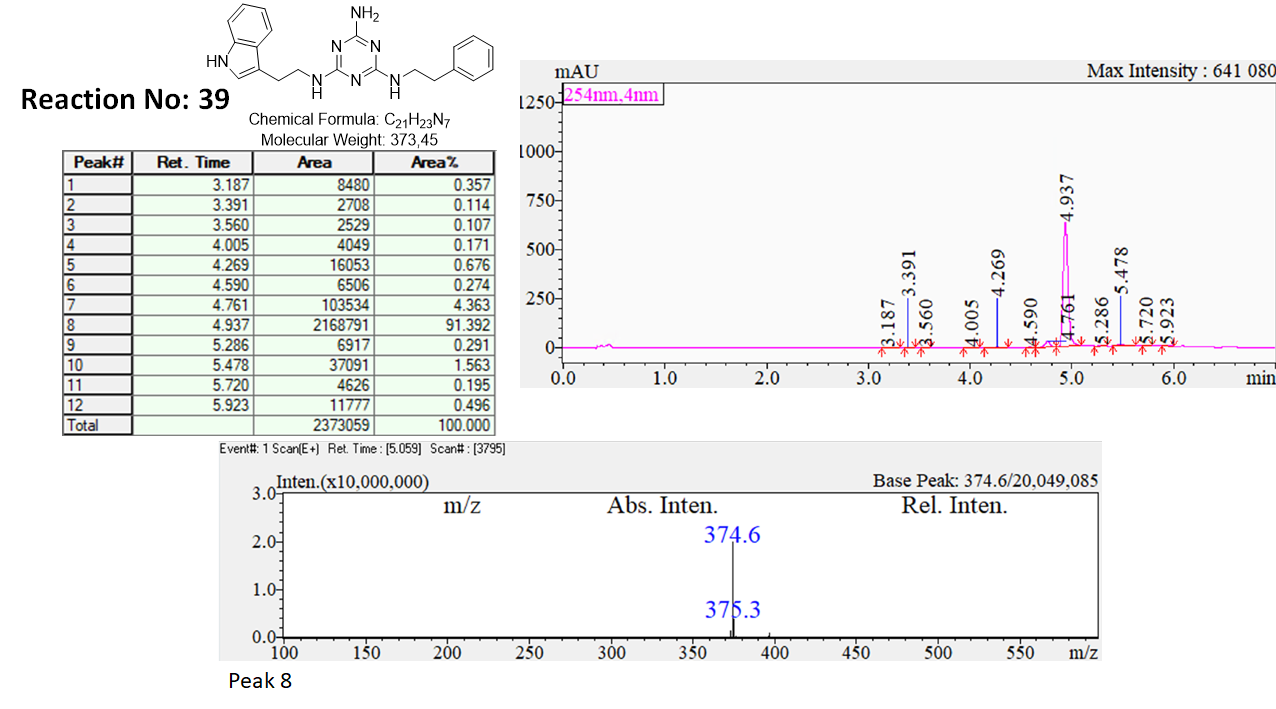


LC-MS according to method A


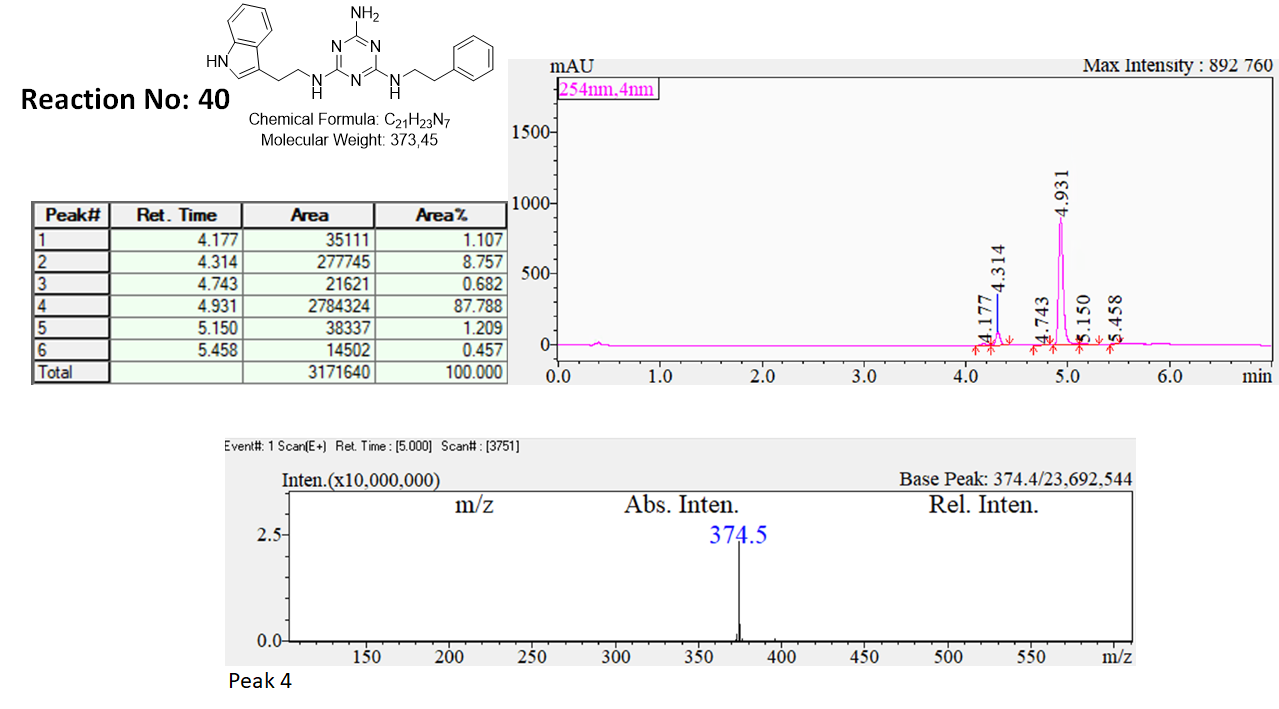


LC-MS according to method A


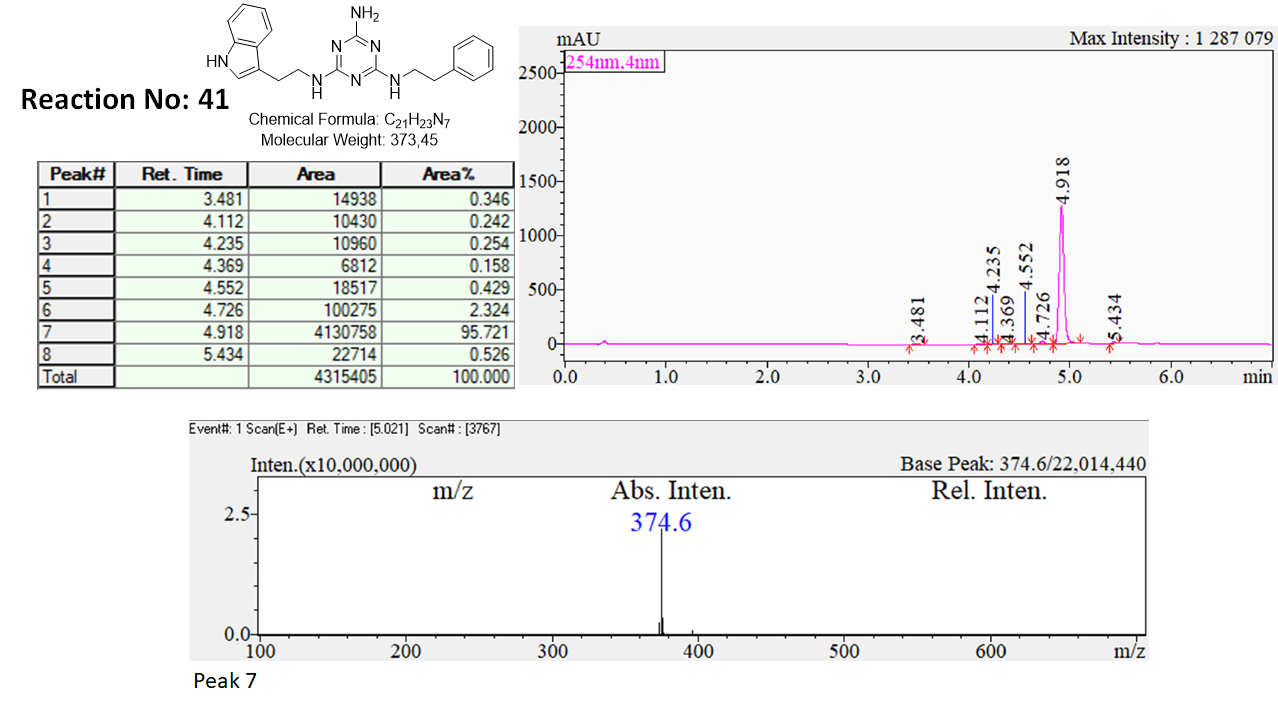


LC-MS according to method A


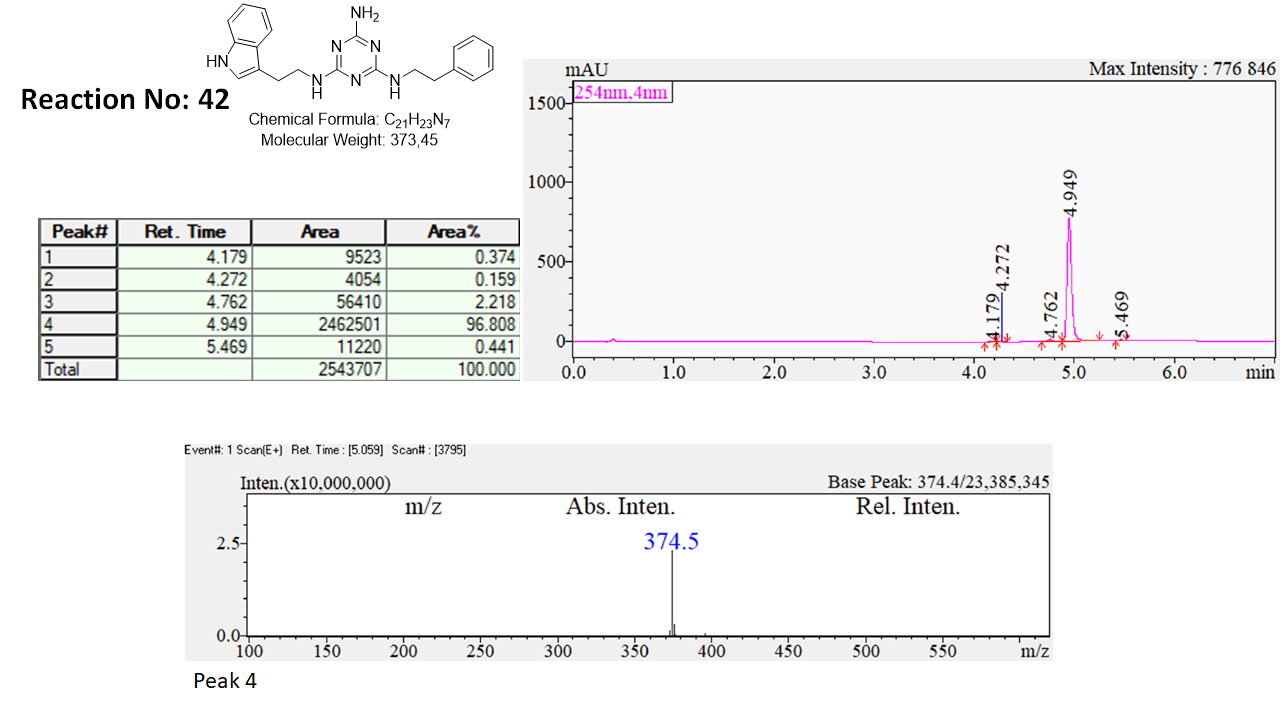


LC-MS according to method A


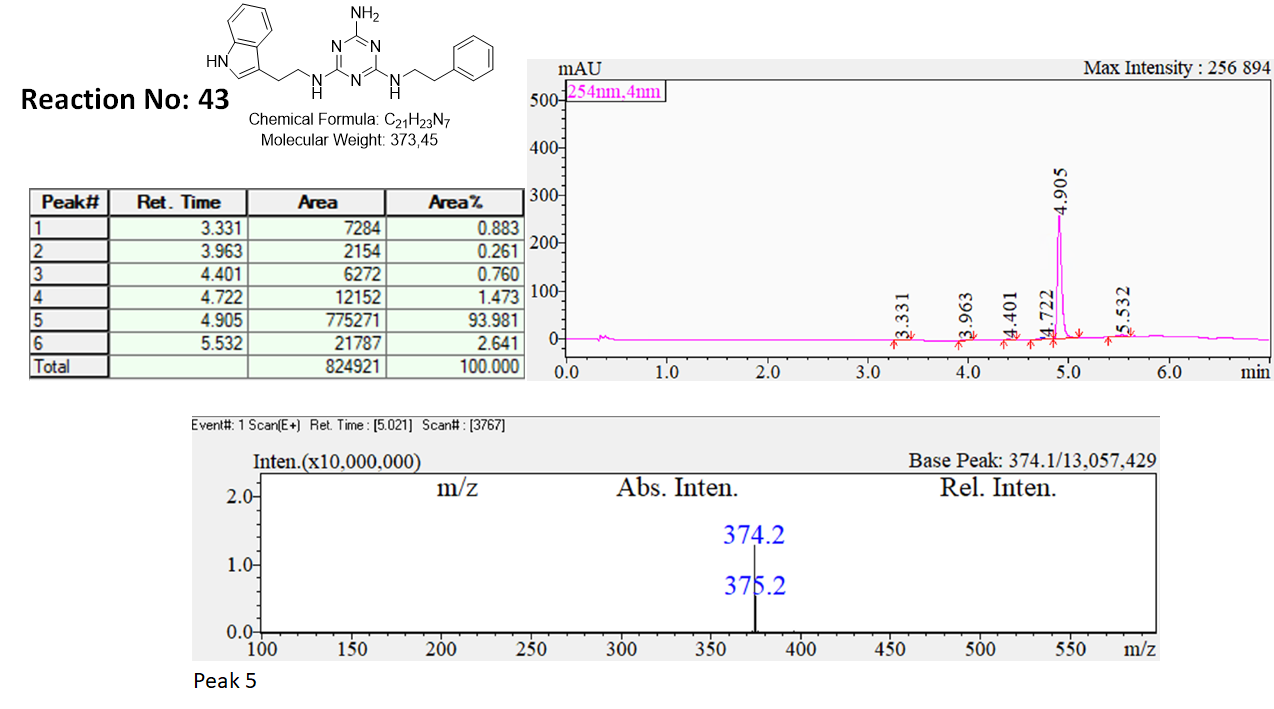


LC-MS according to method A


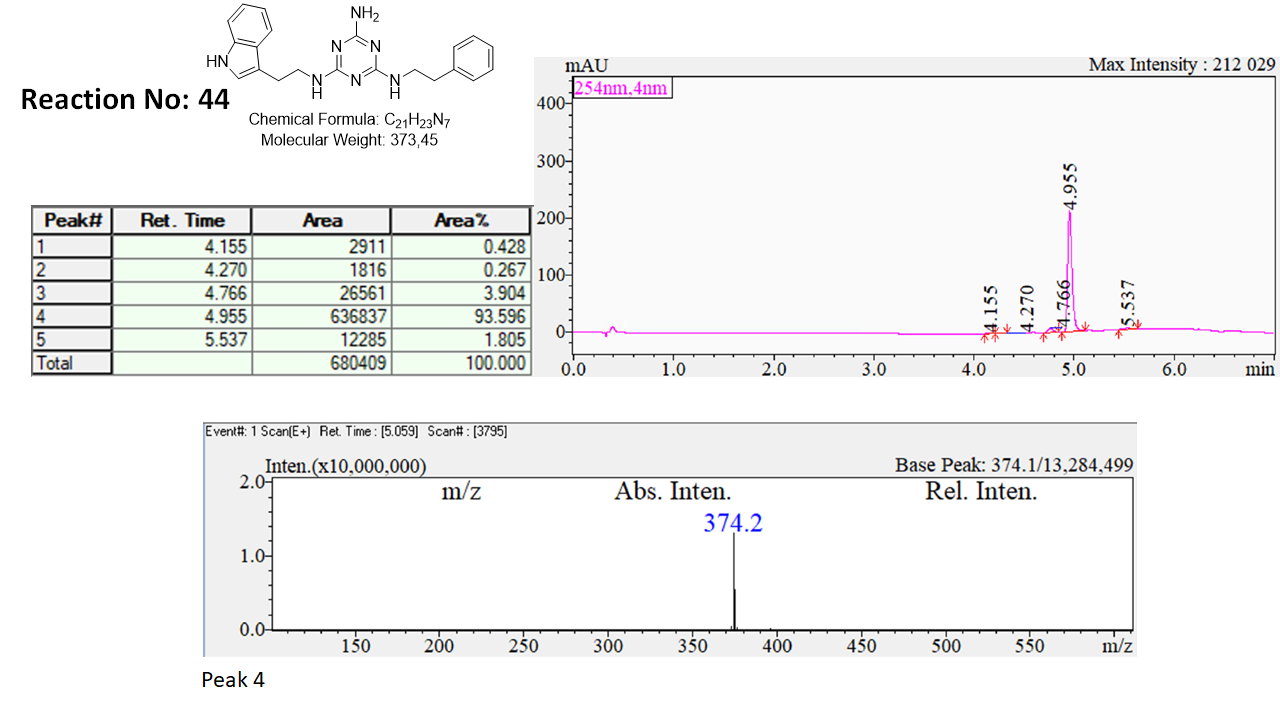


LC-MS according to method A


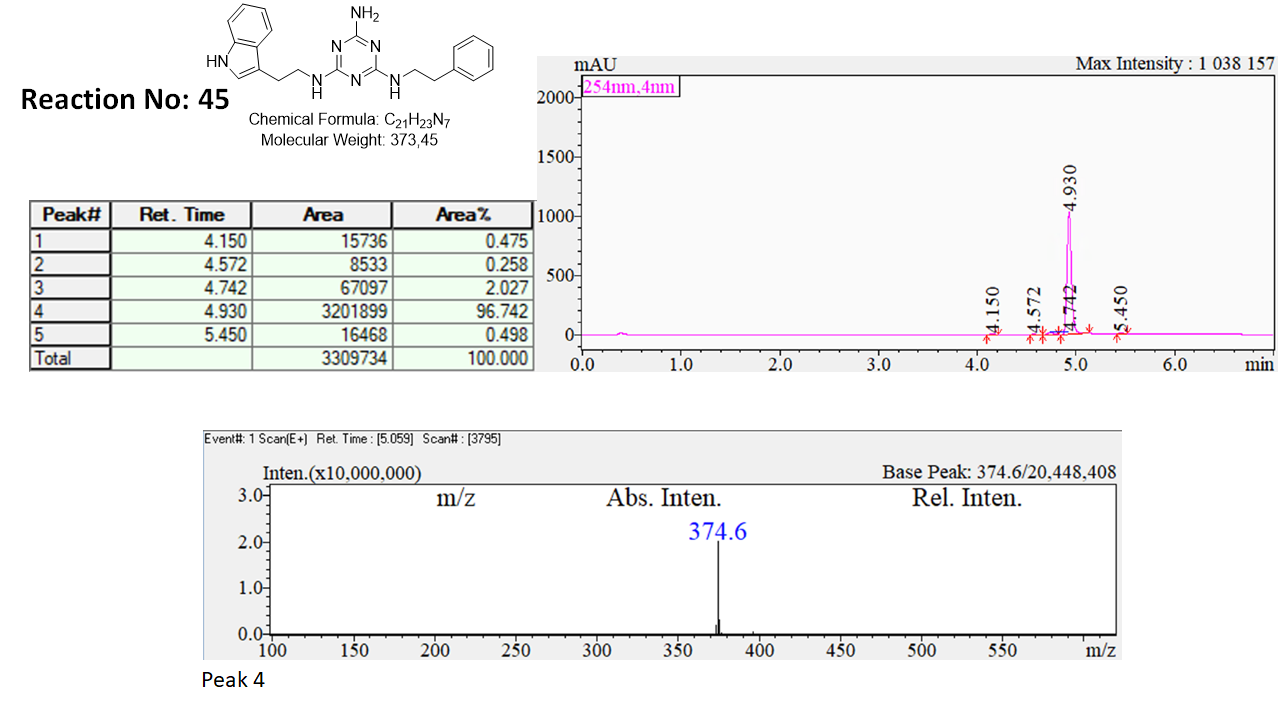


LC-MS according to method A


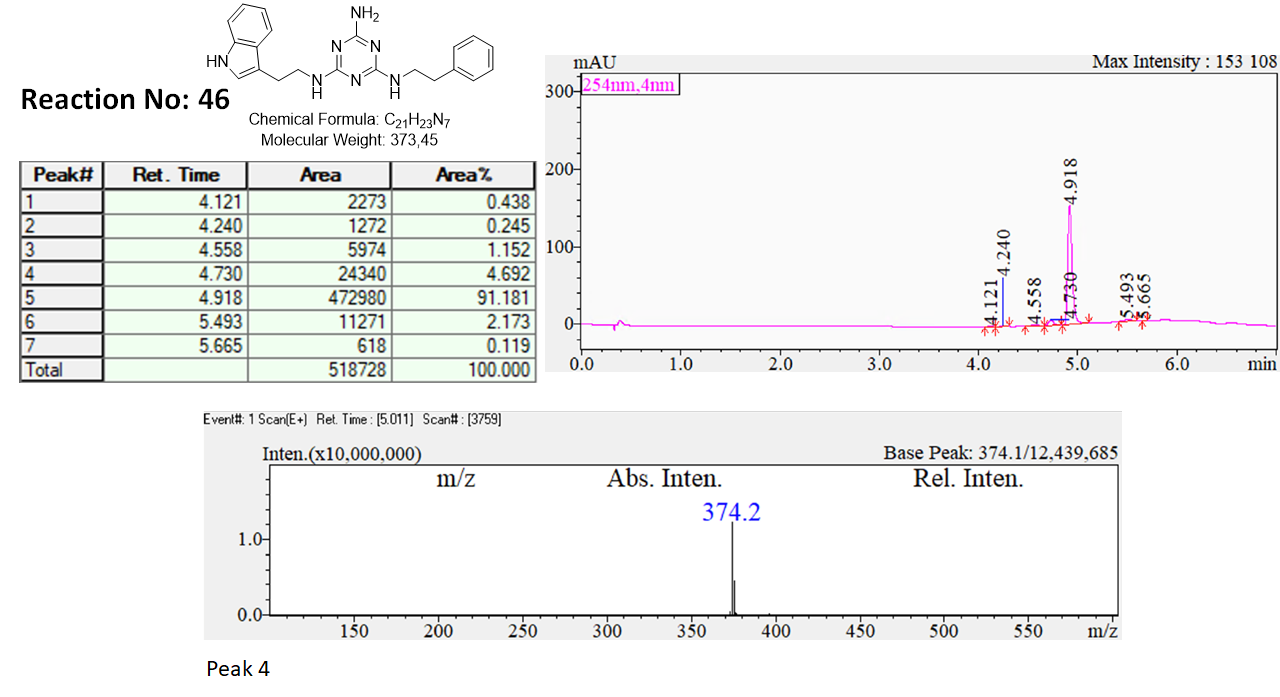


LC-MS according to method A


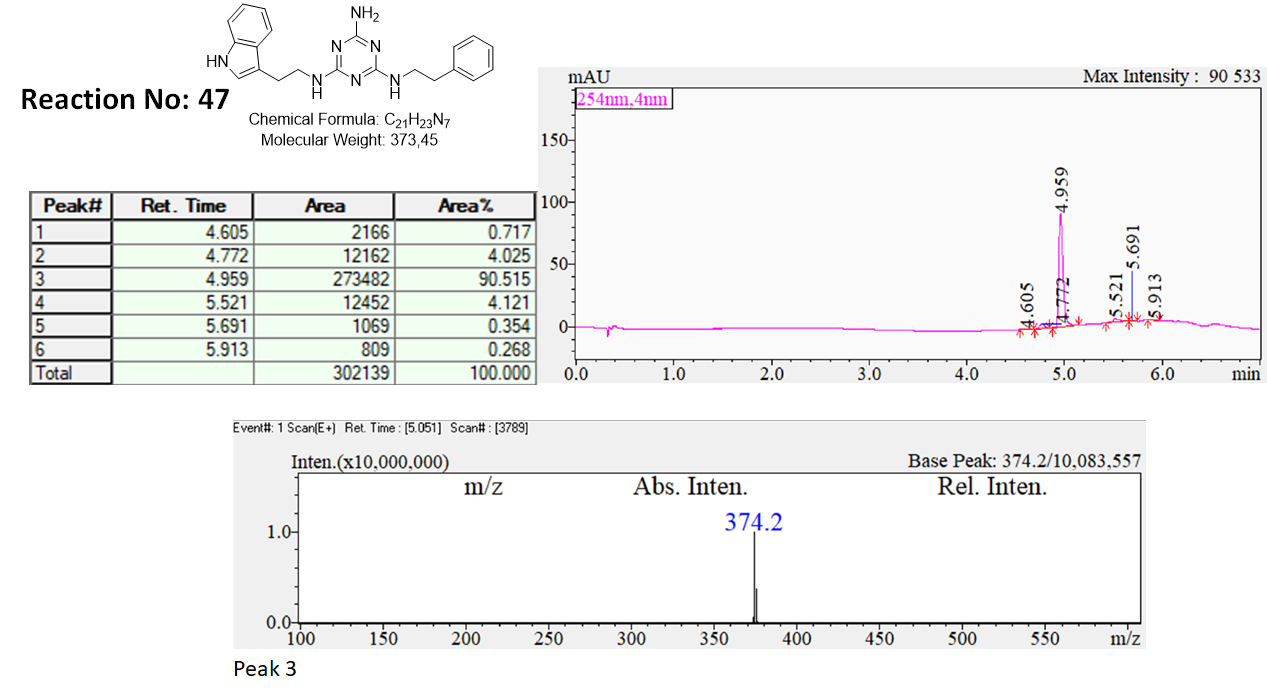


LC-MS according to method A


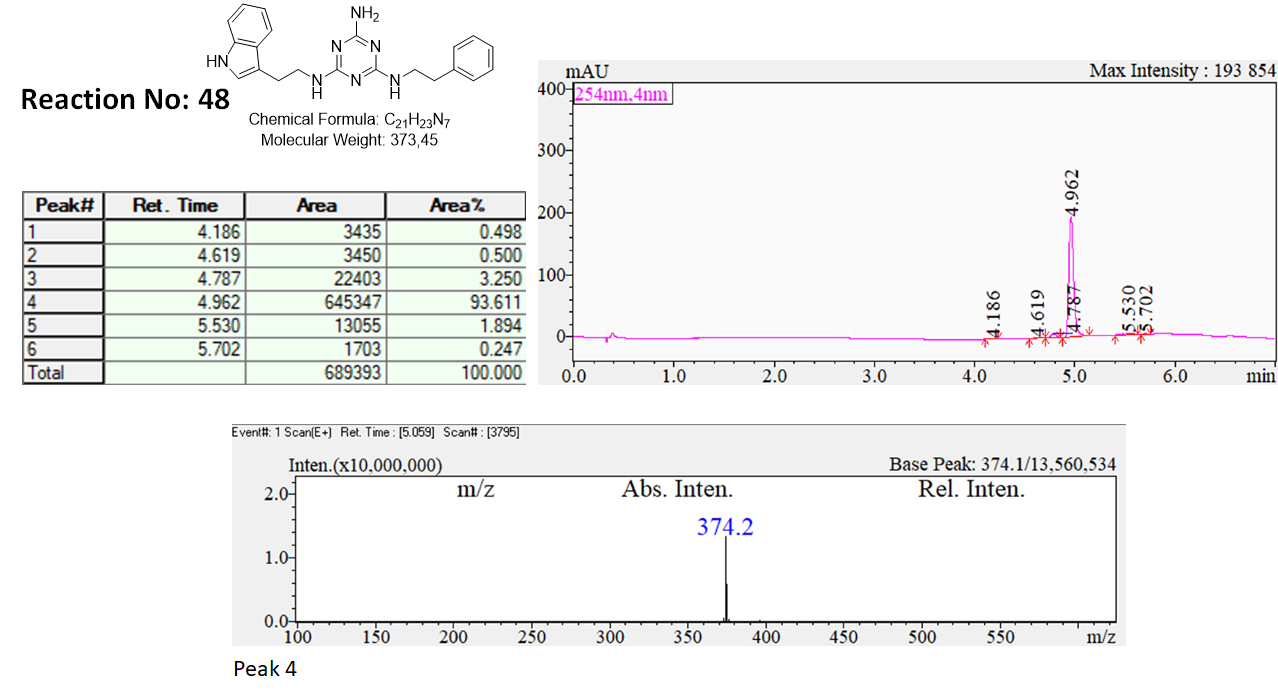


LC-MS according to method A


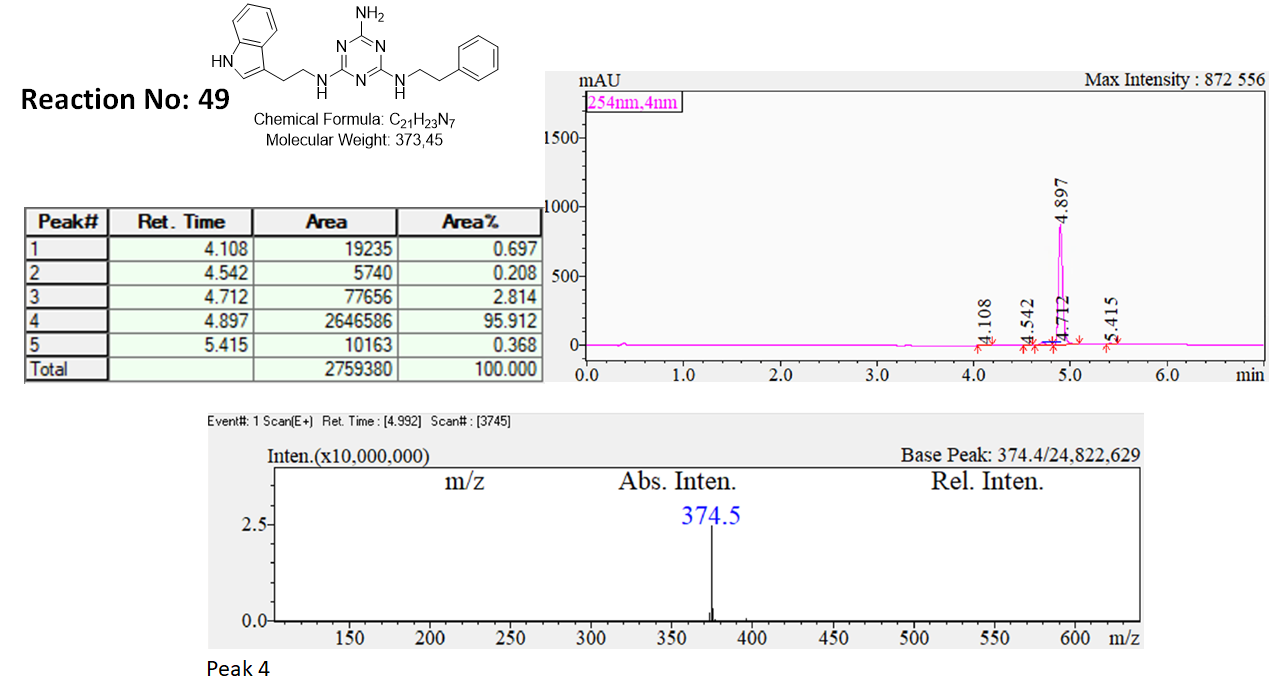


LC-MS according to method A


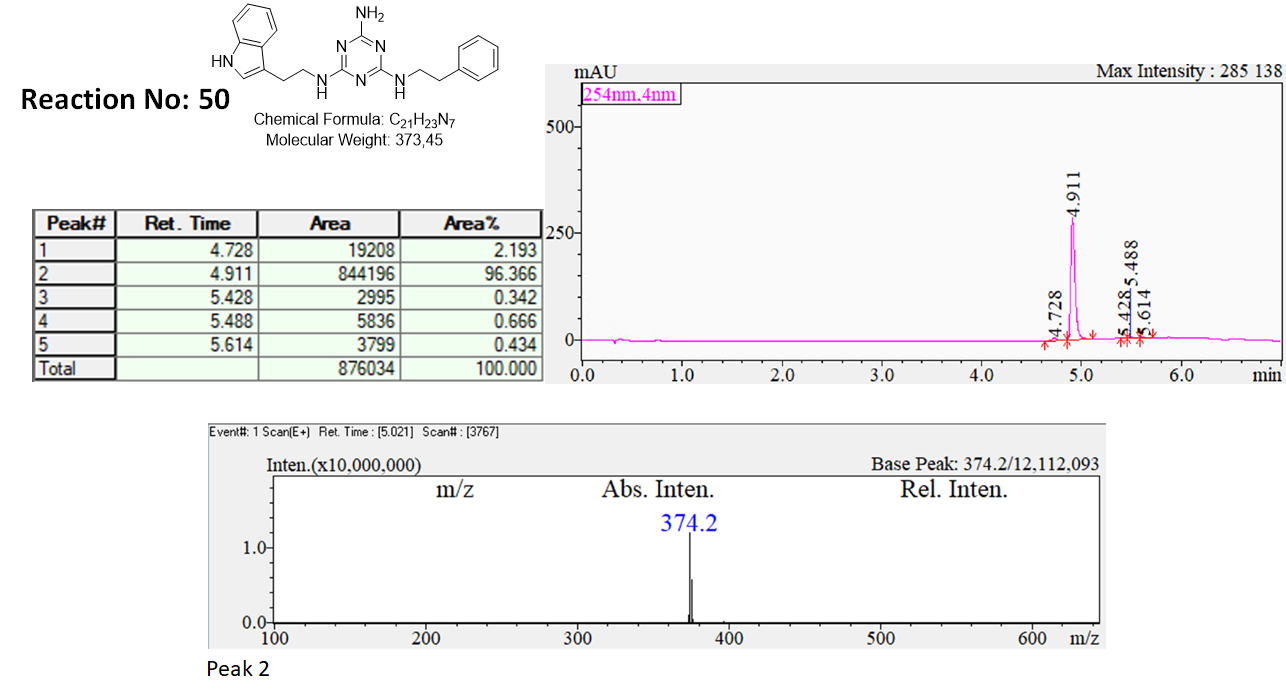


LC-MS according to method A


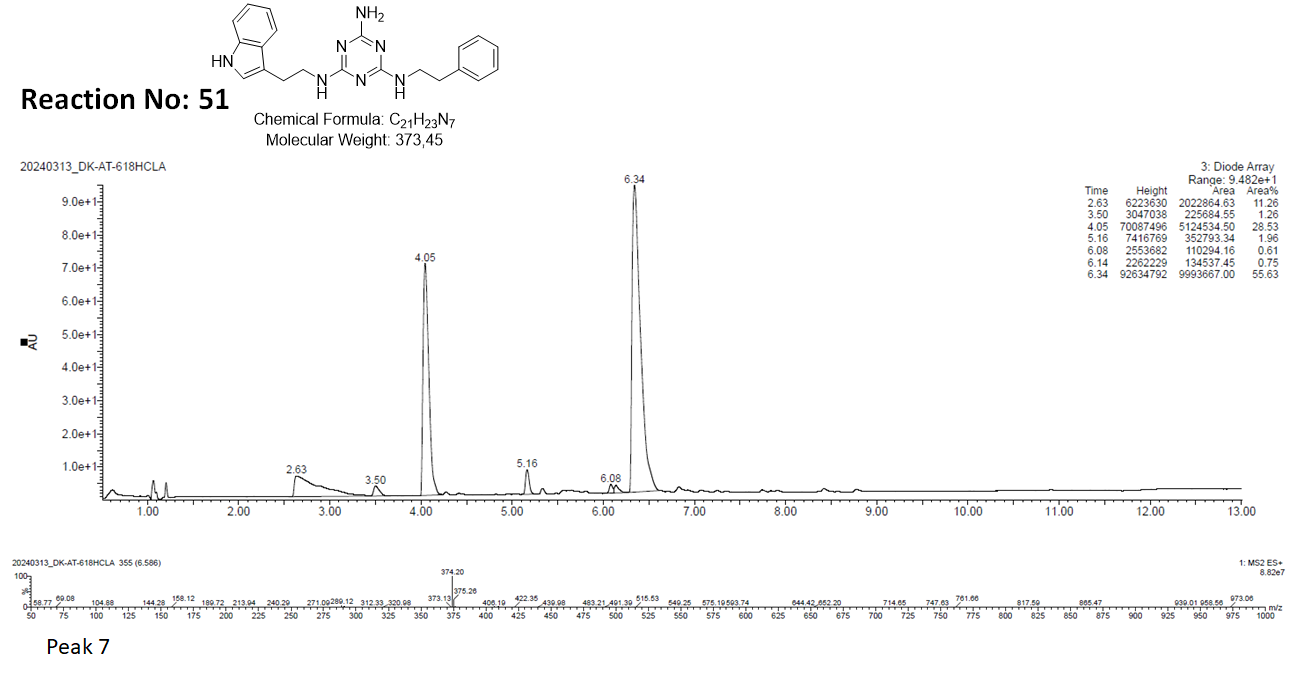


LC-MS according to method B


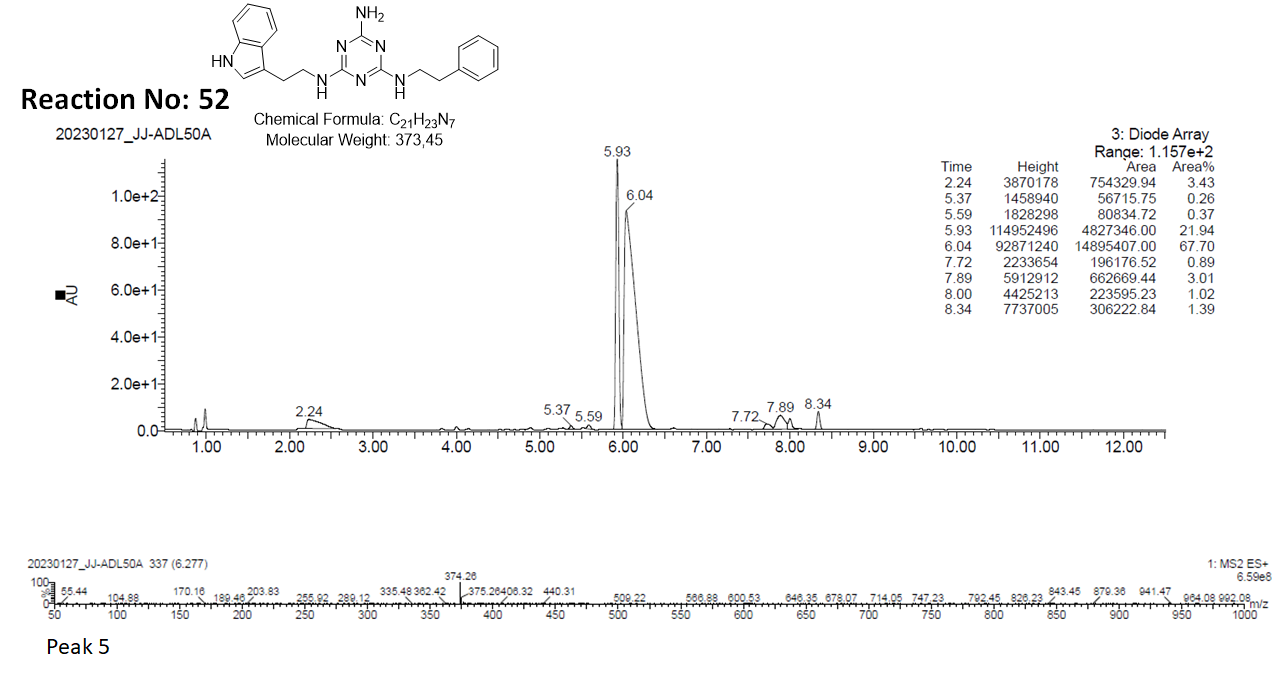


LC-MS according to method B


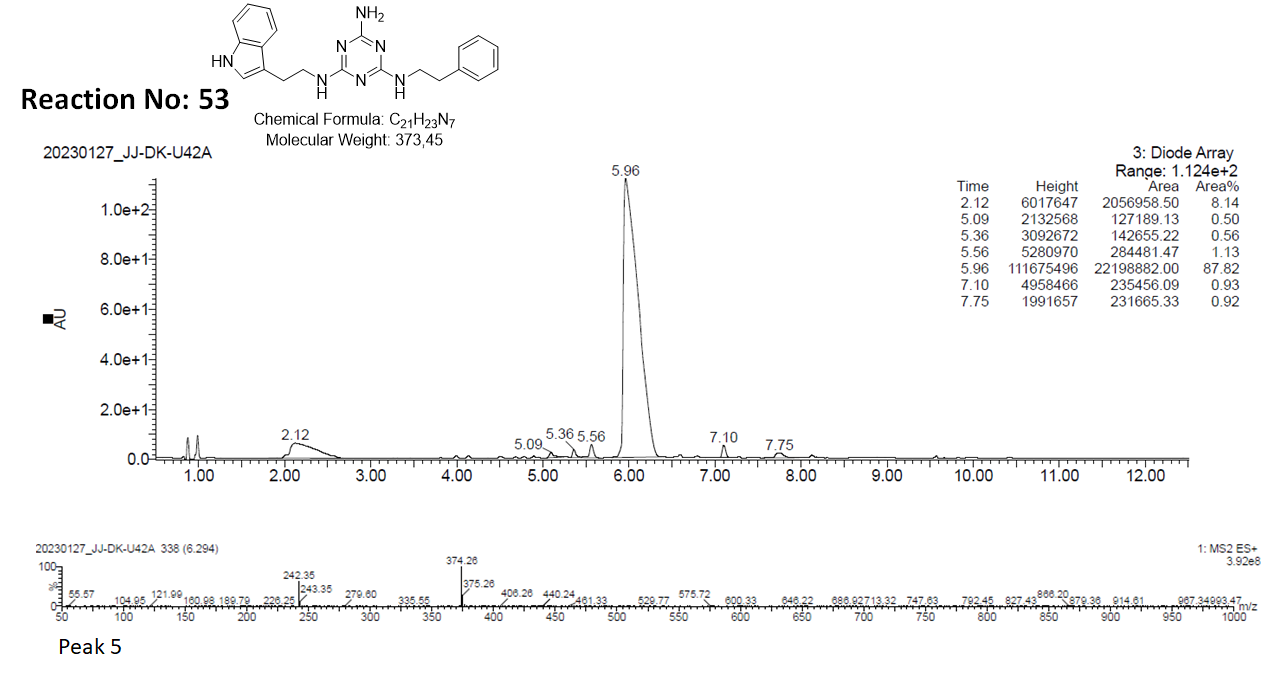


LC-MS according to method B


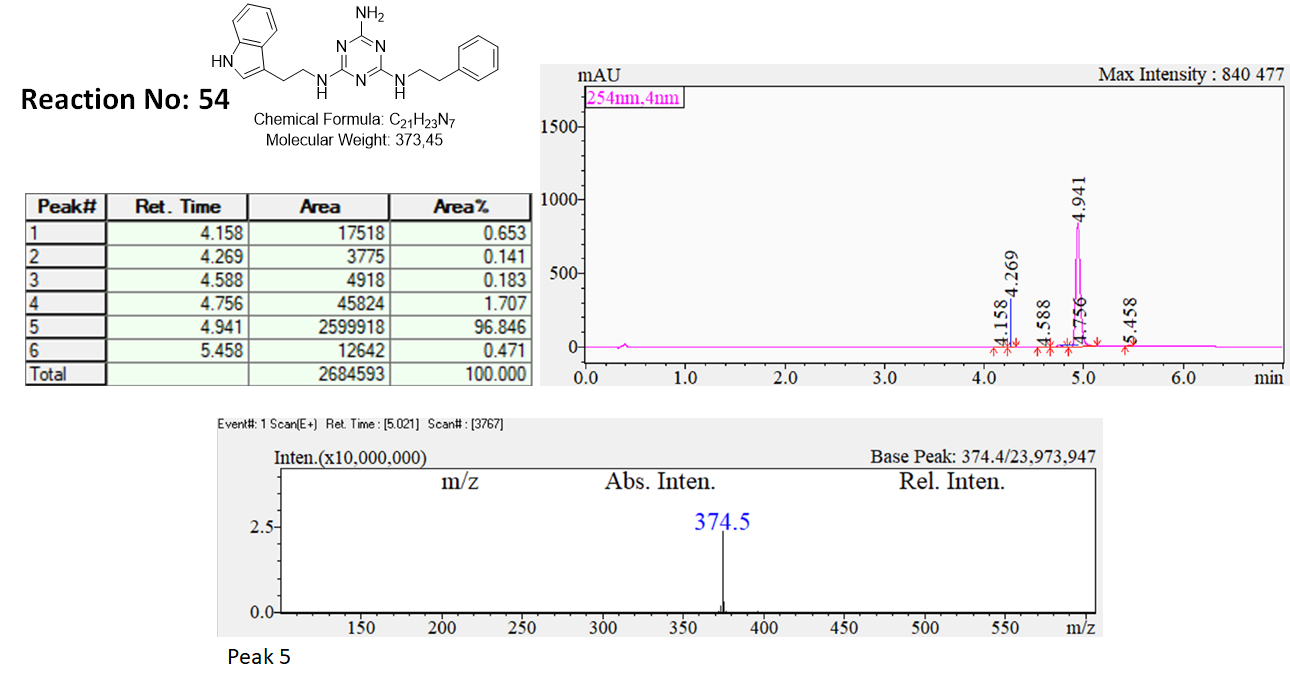


LC-MS according to method A

LC-MS according to method A

**UPLC-MS spectra for crude 3a, 3b, 3d, 4a-4c, 5a, 5b compounds**

LC-MS according to method B

LC-MS according to method B

LC-MS according to method B


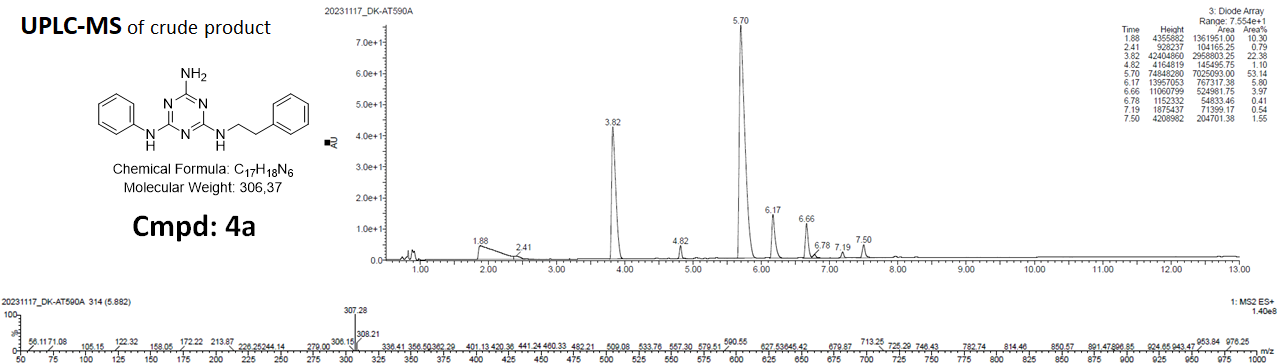


LC-MS according to method B

LC-MS according to method B

LC-MS according to method B

LC-MS according to method B

LC-MS according to method B

**Spectra for final compounds 3, 3a-3d to 5a-5d (UPLC-MS, HRMS, ^1^H NMR, ^13^C NMR)**


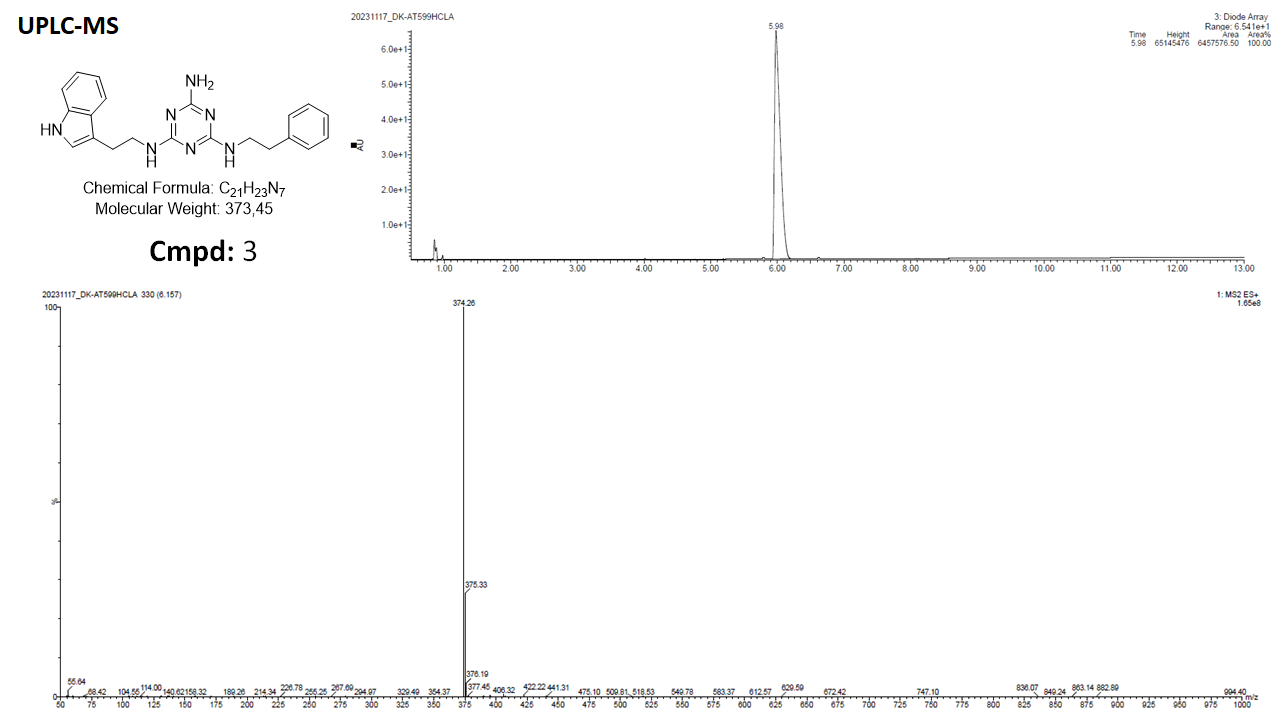


LC-MS according to method B

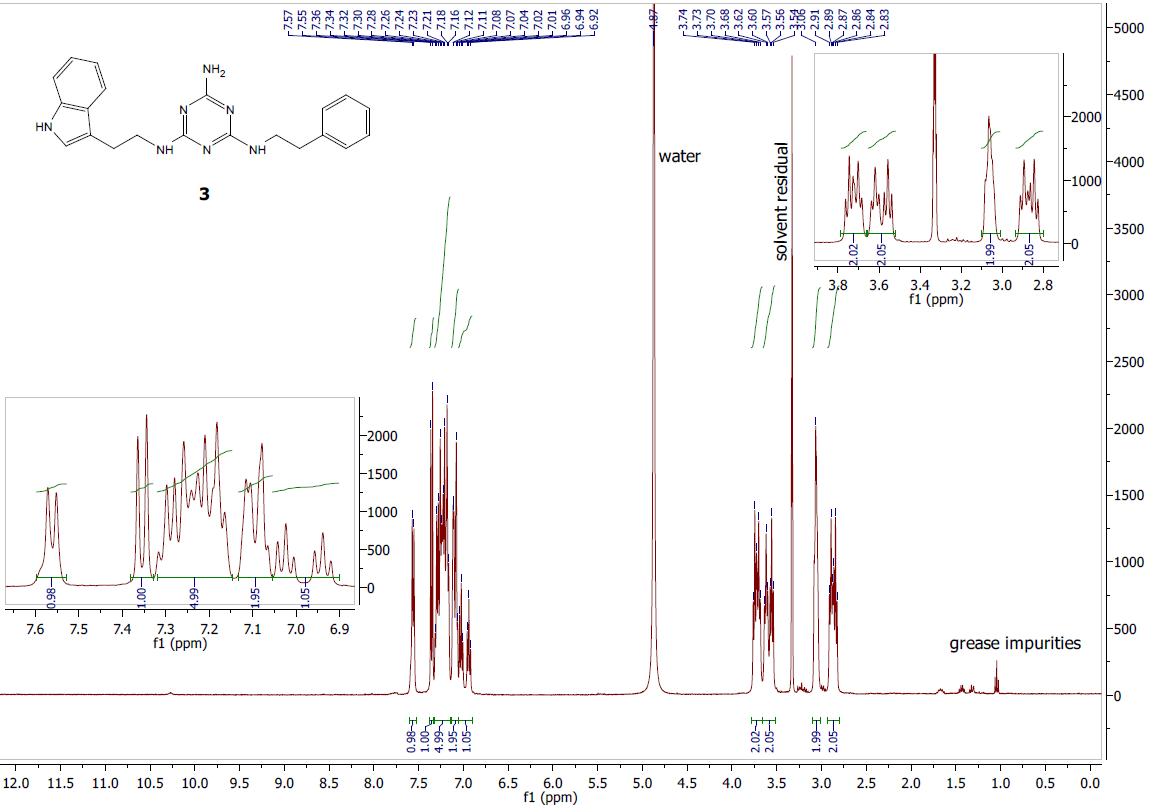


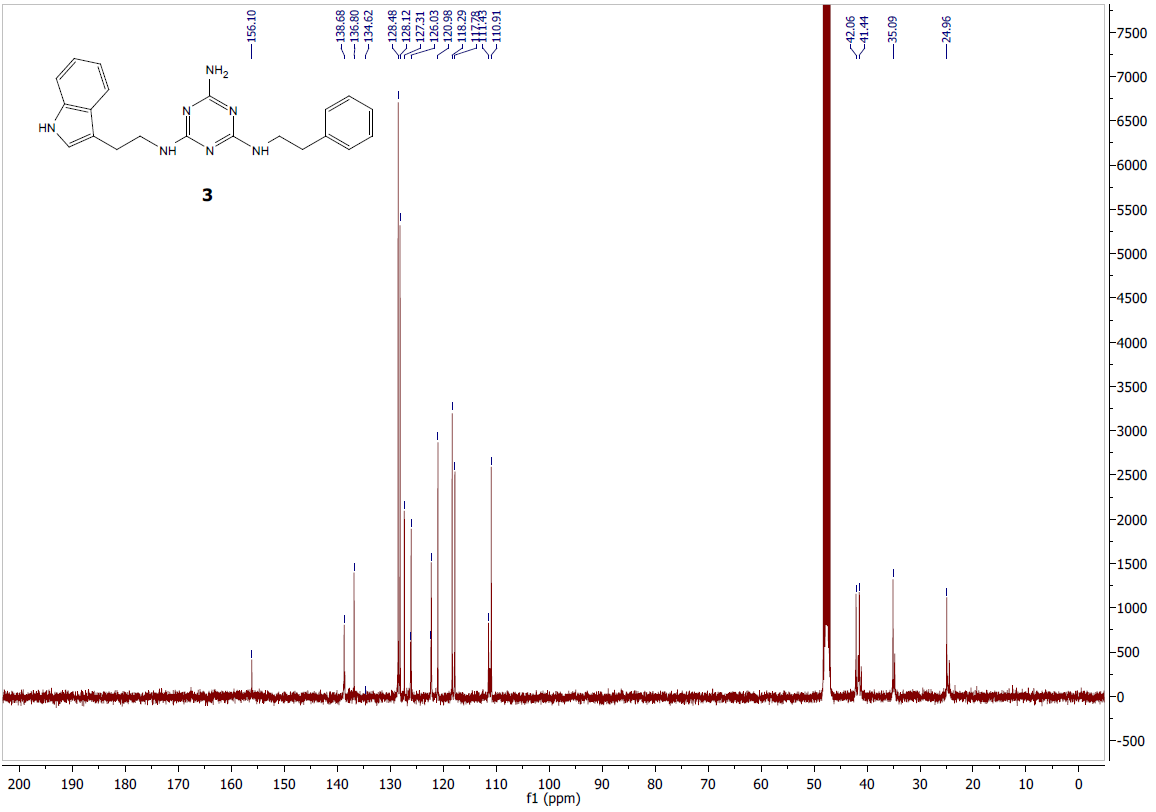


LC-MS according to method B


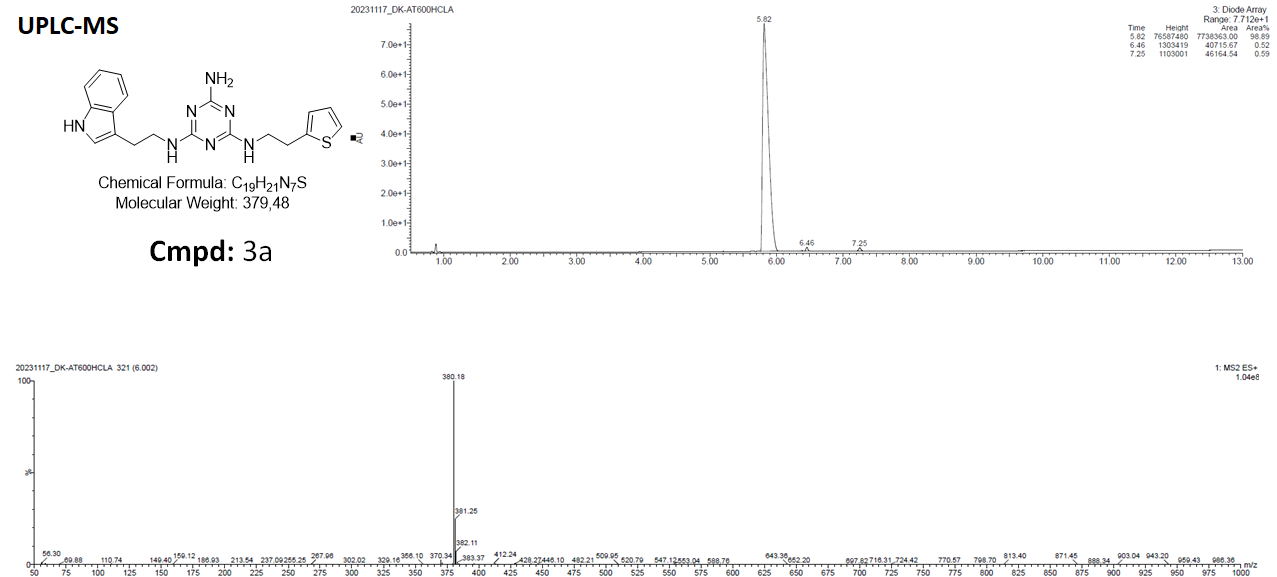


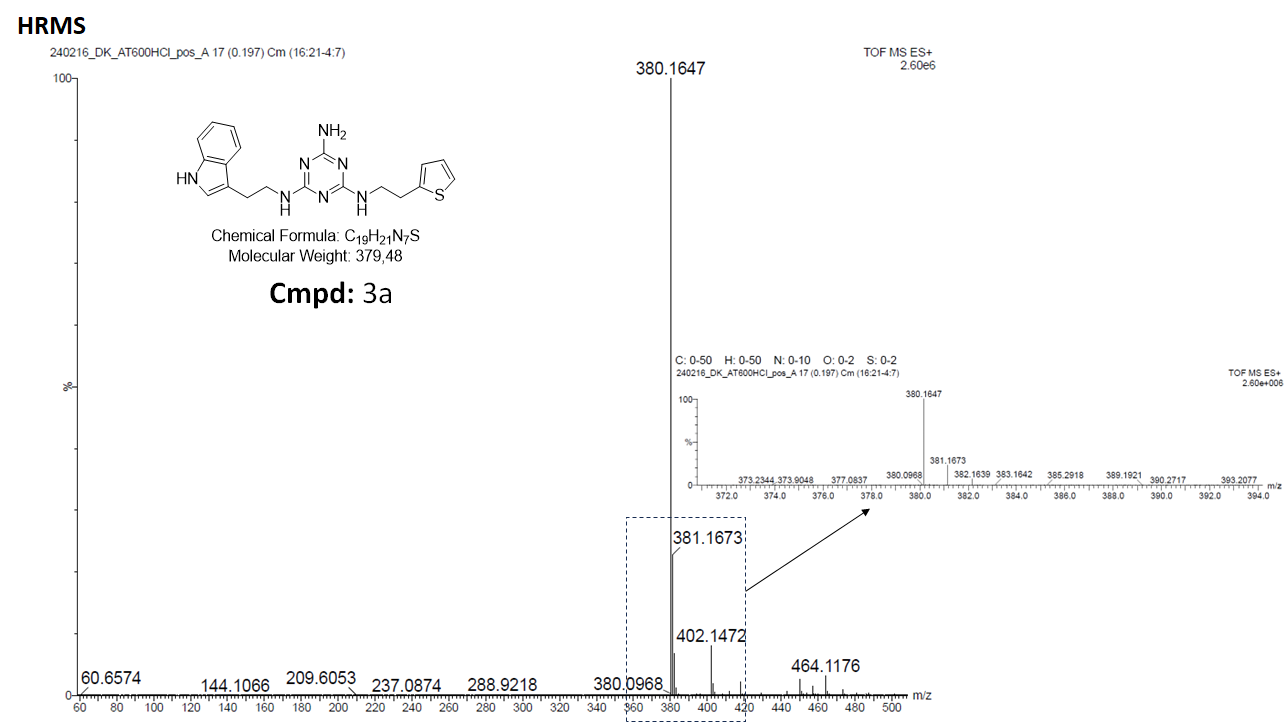


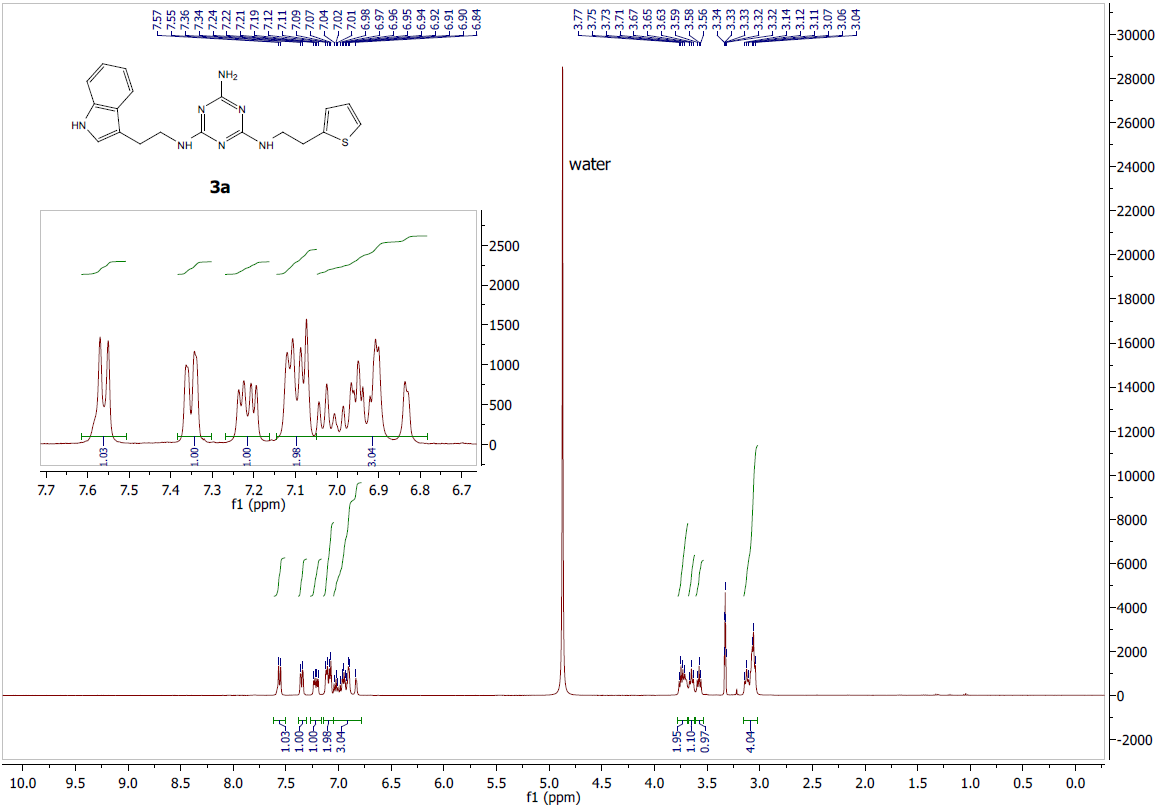


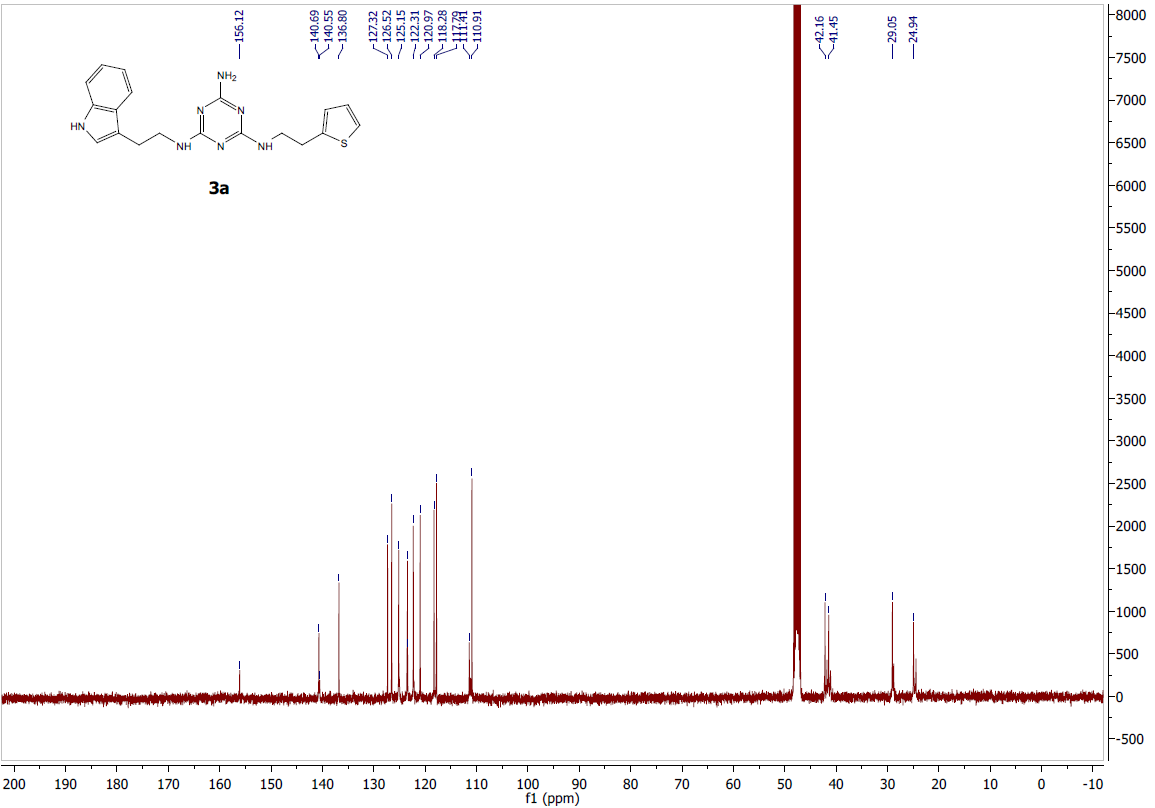


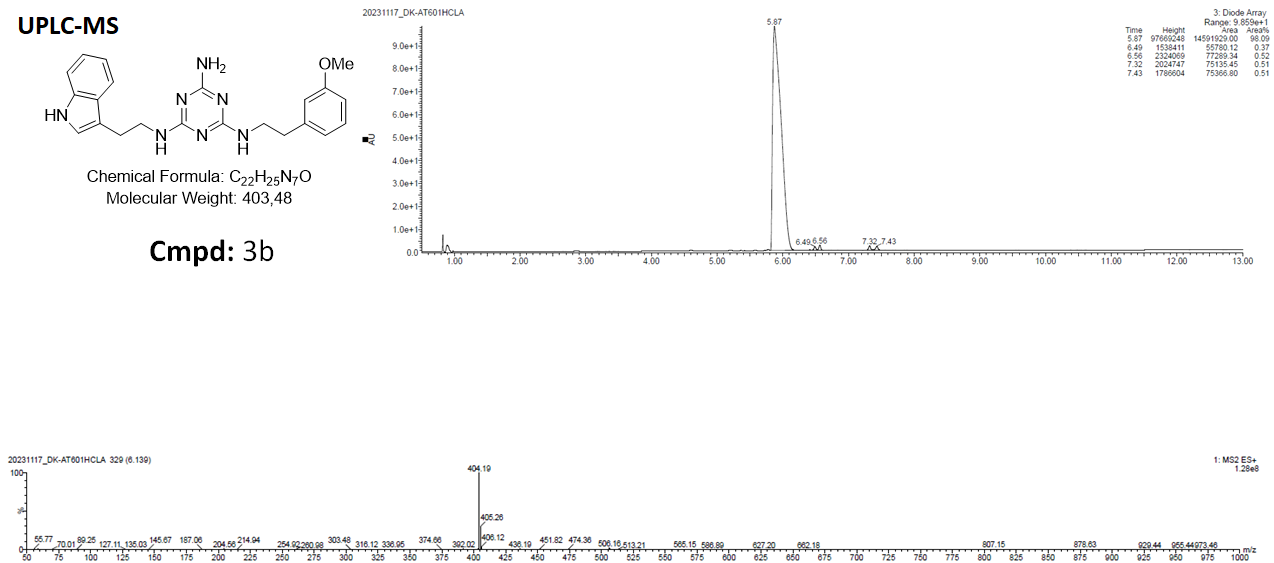


LC-MS according to method B


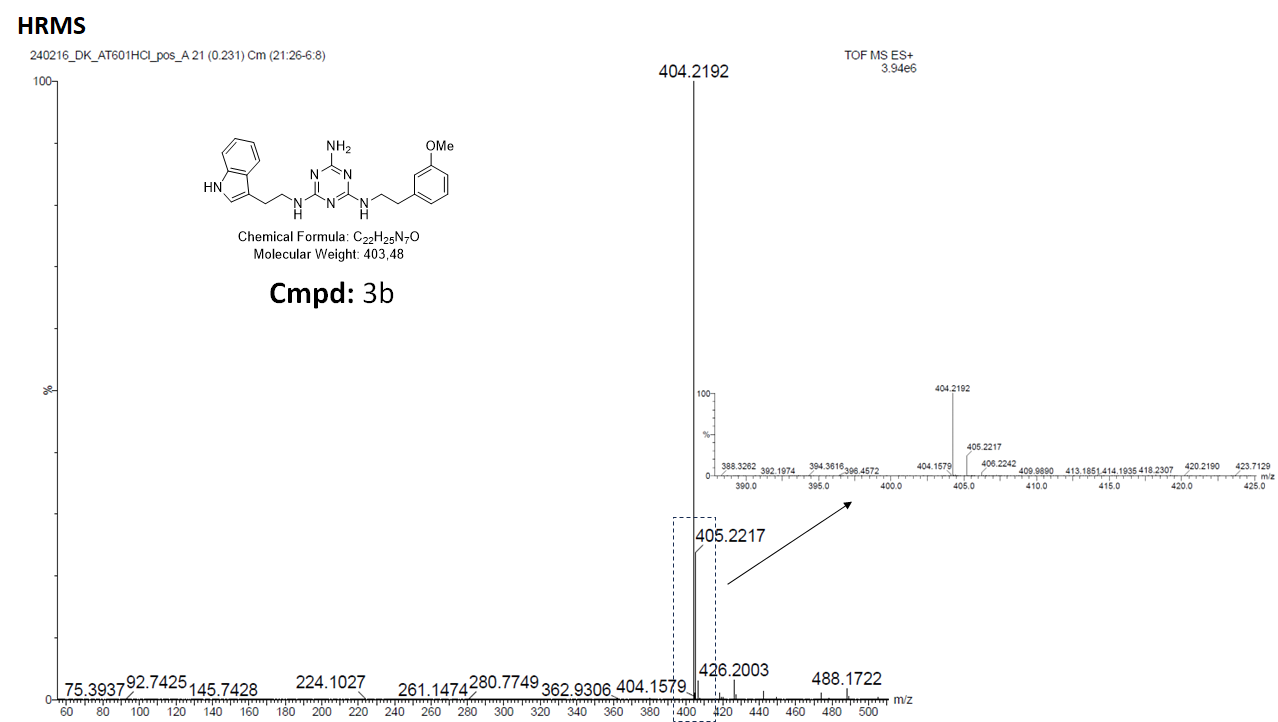


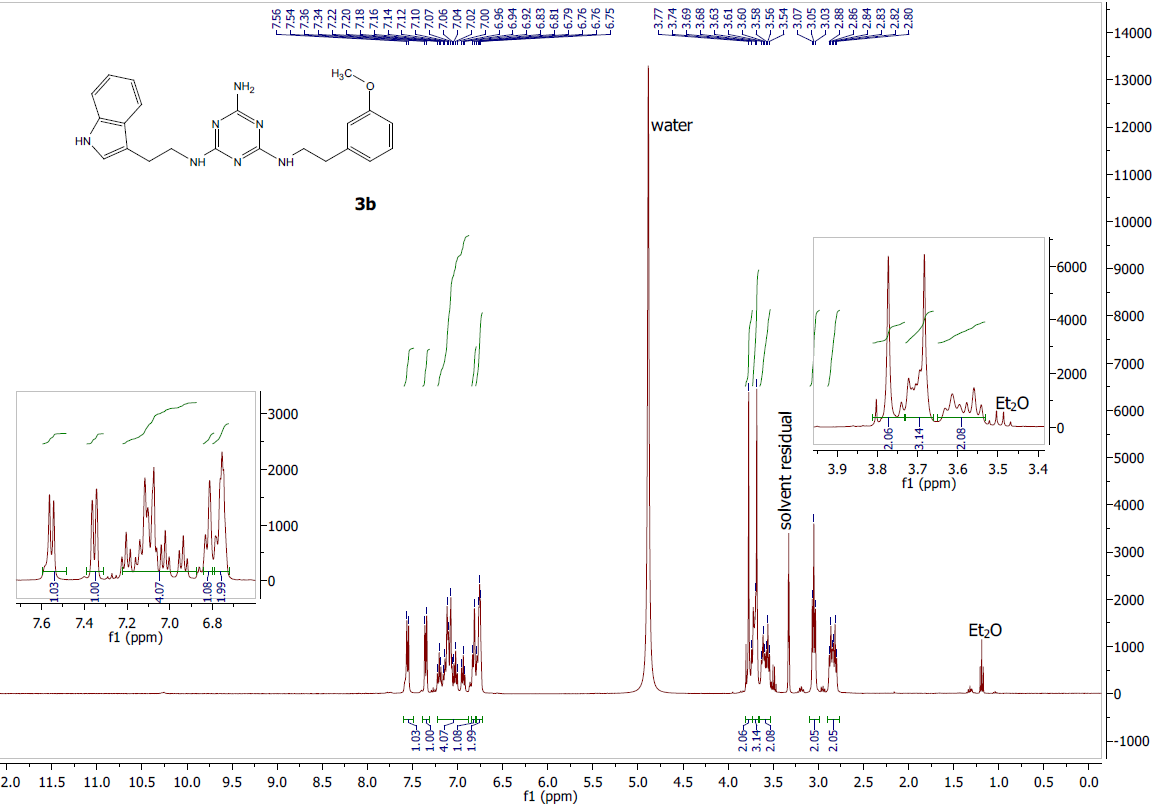


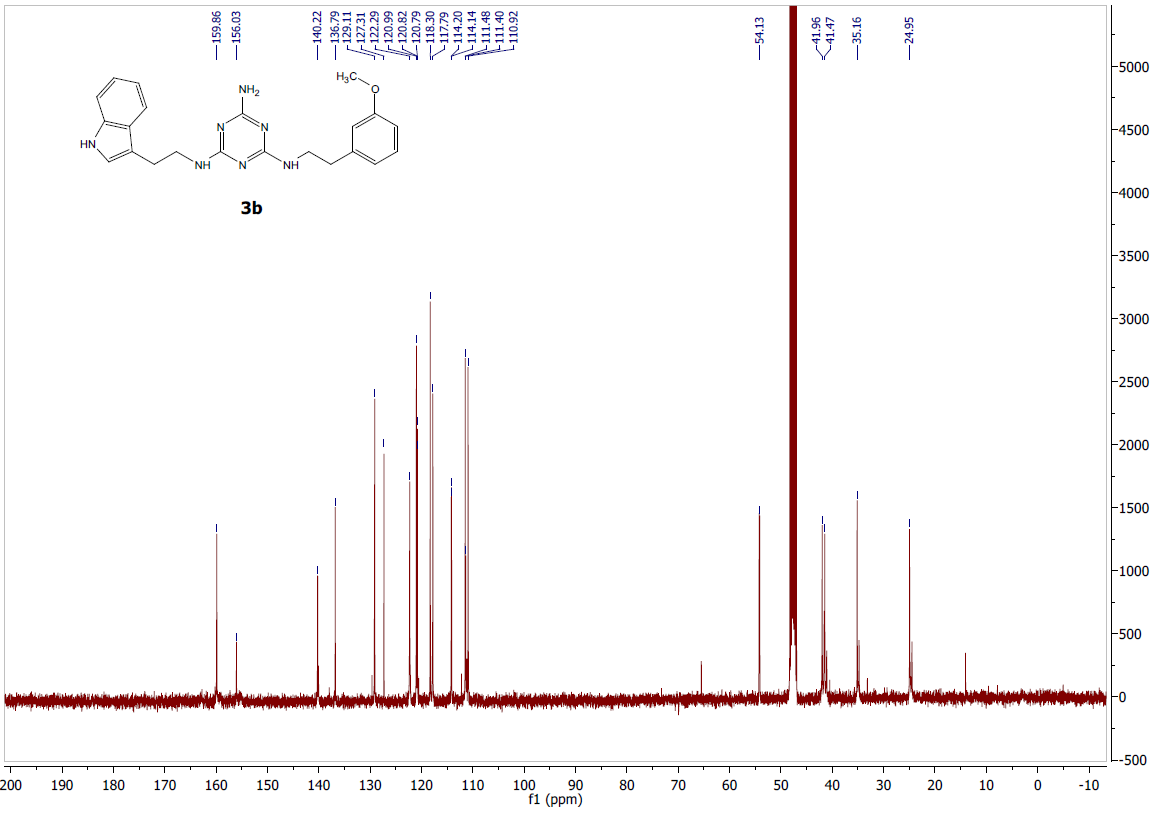


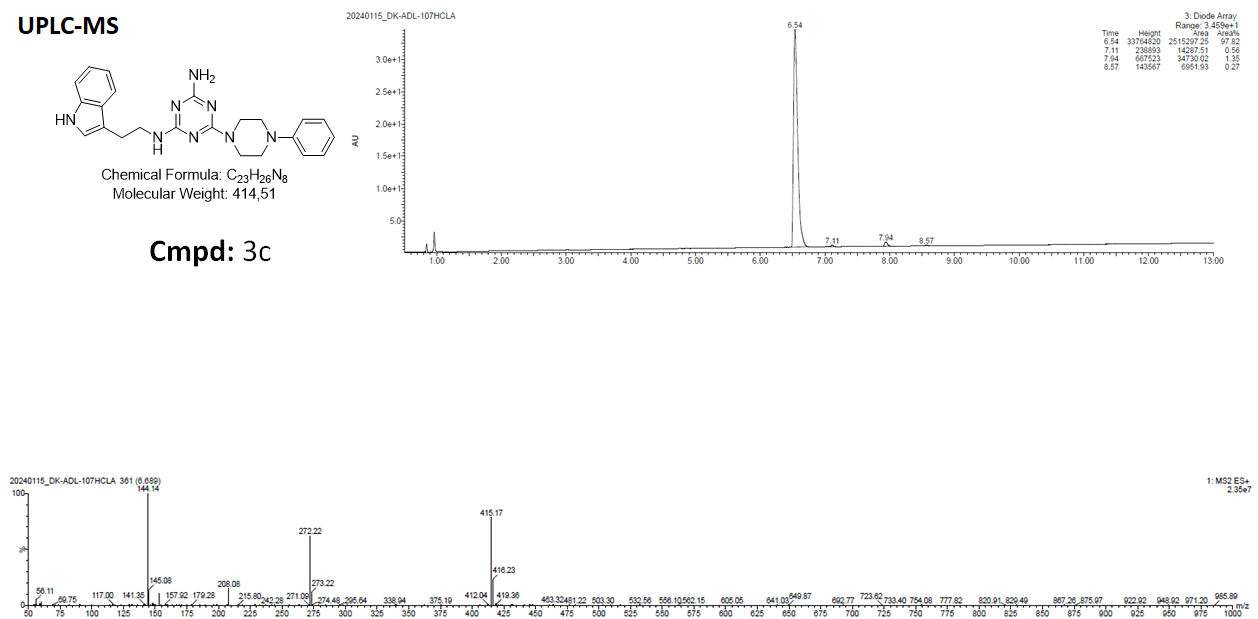


LC-MS according to method B


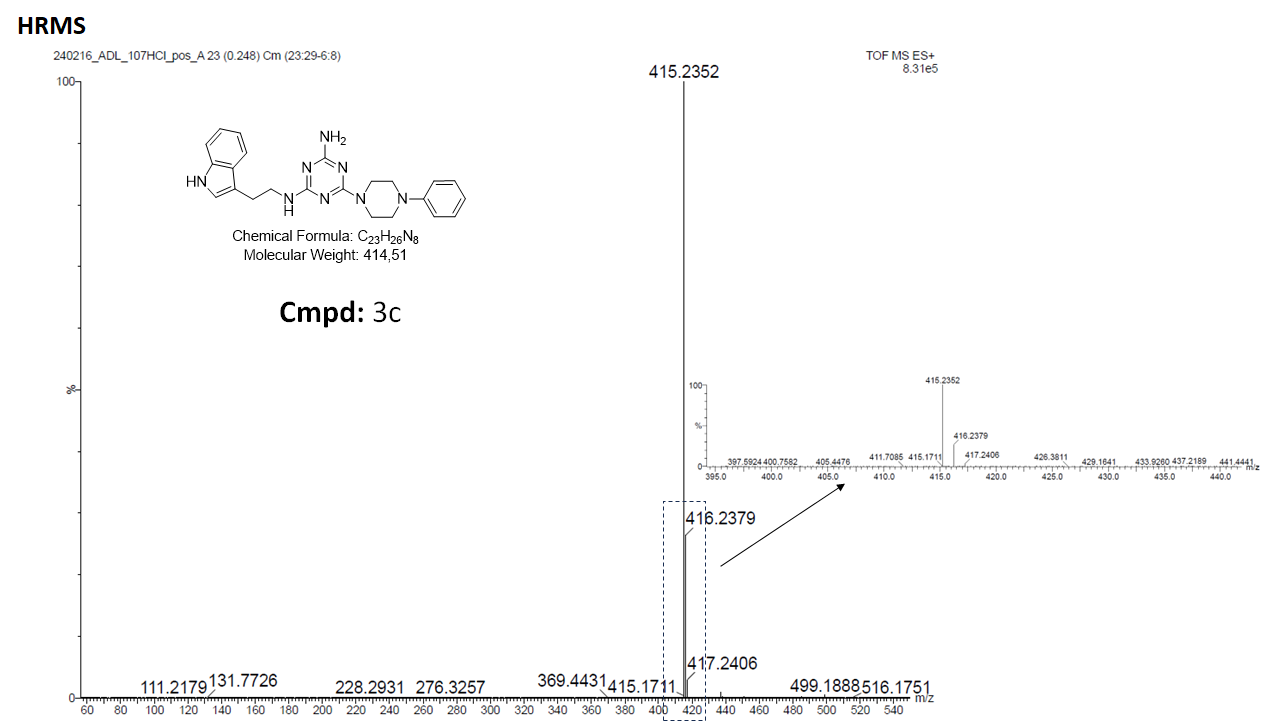


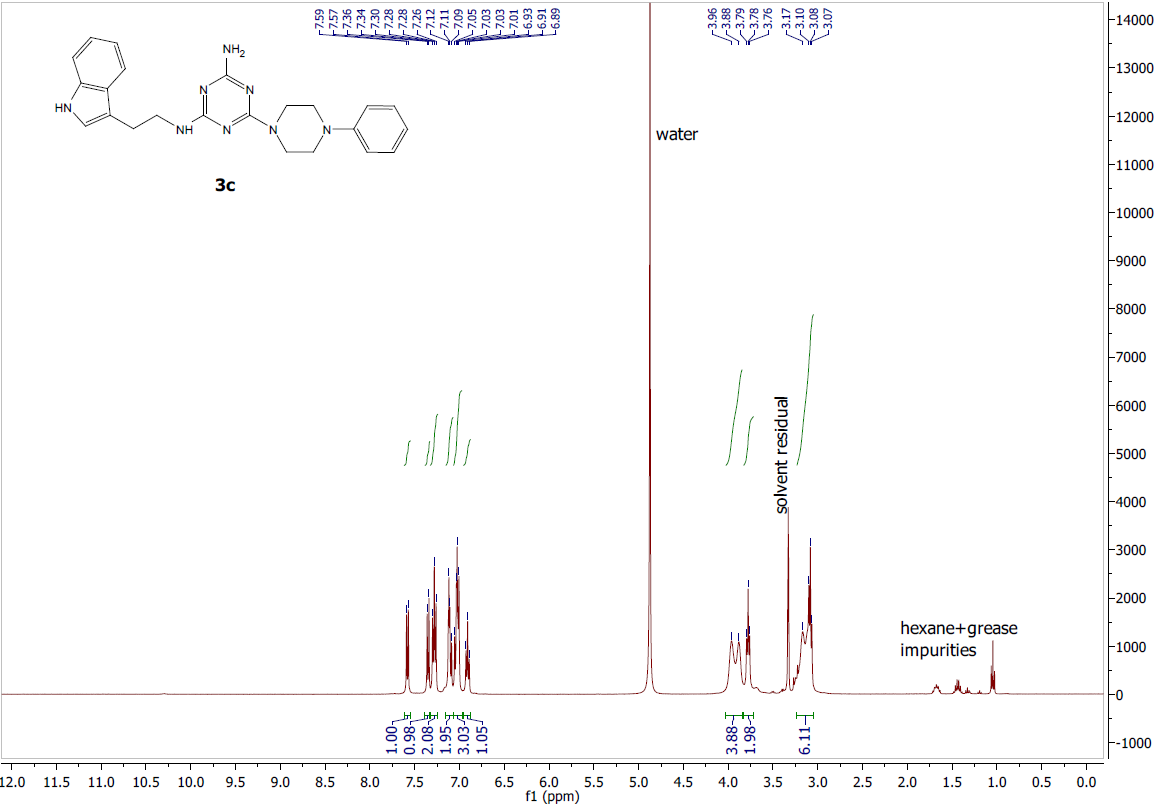


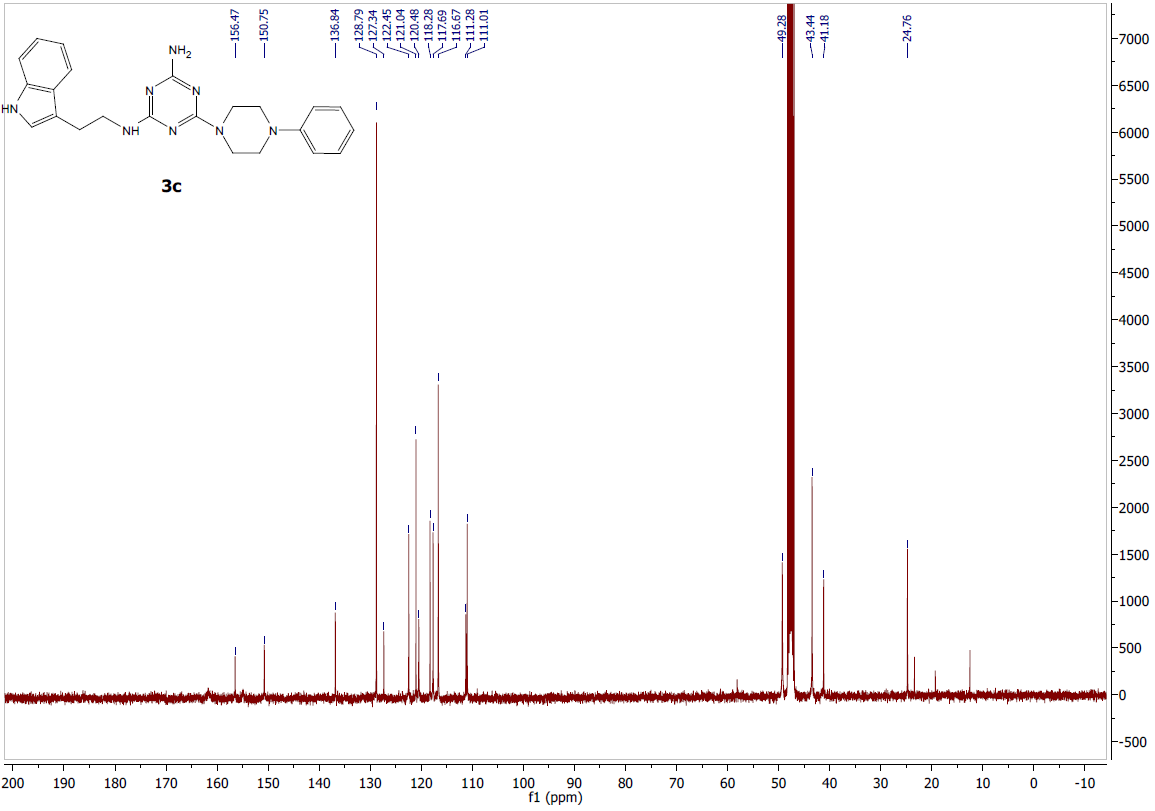


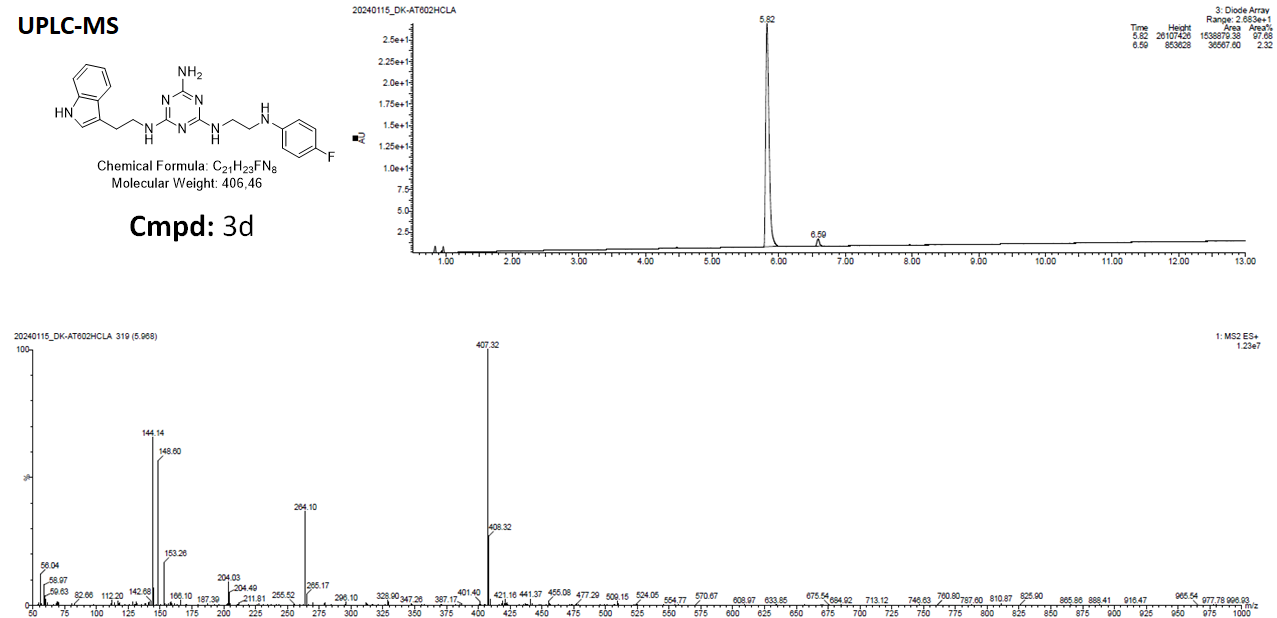


LC-MS according to method B


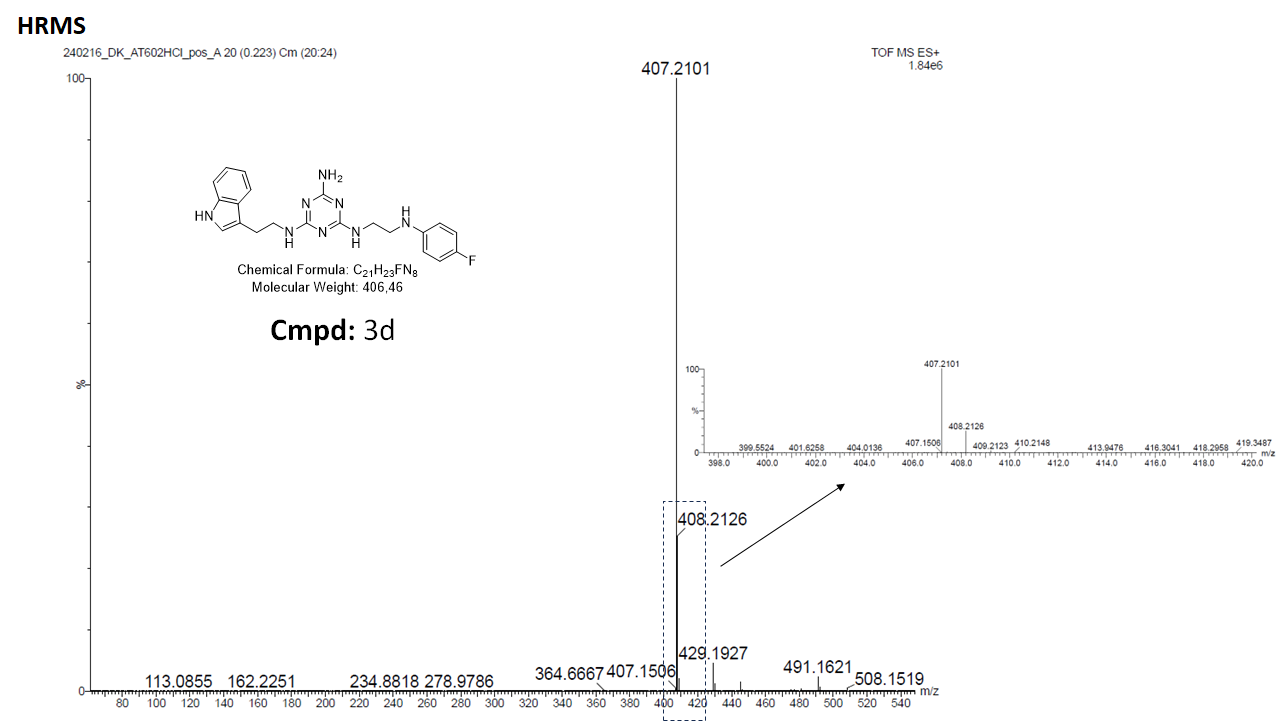


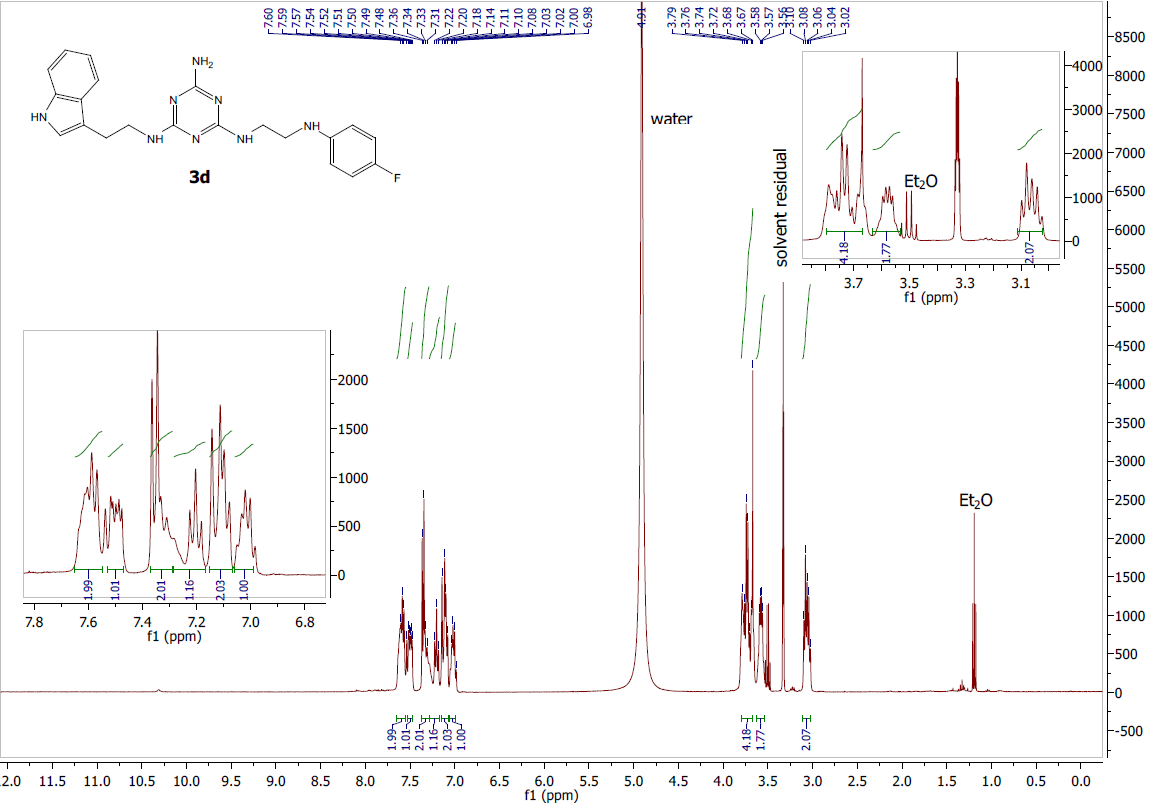


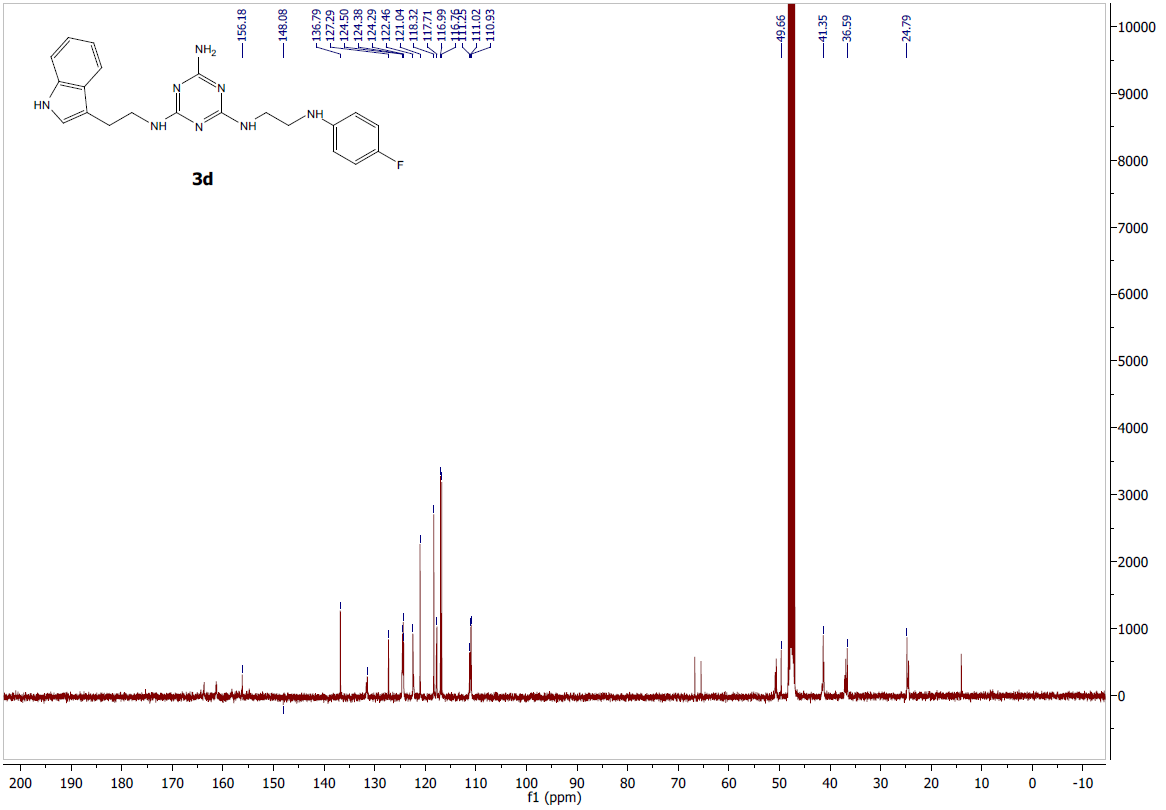

LC-MS according to method B


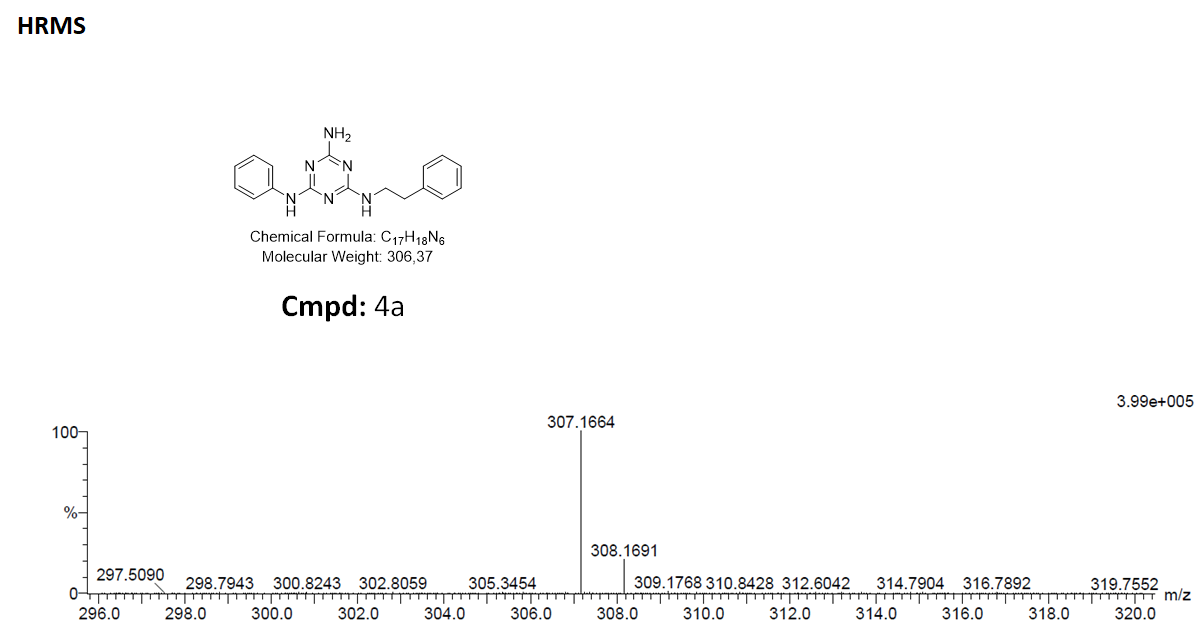


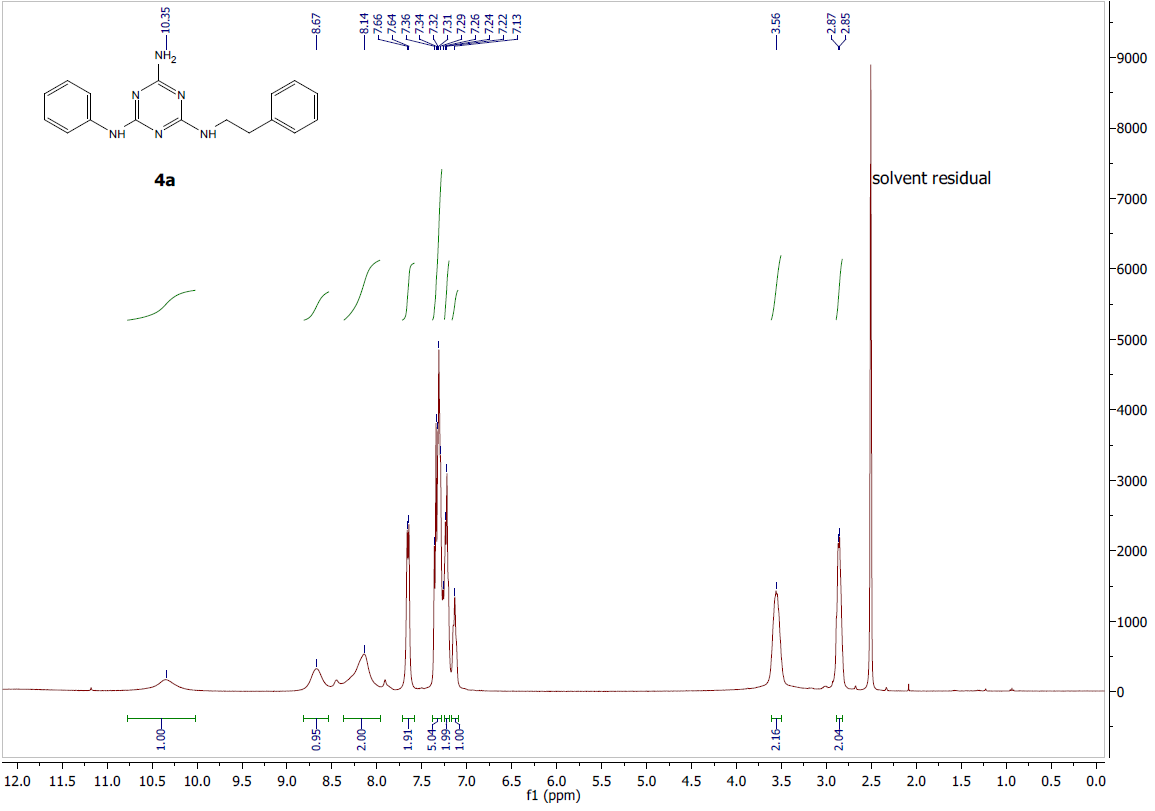


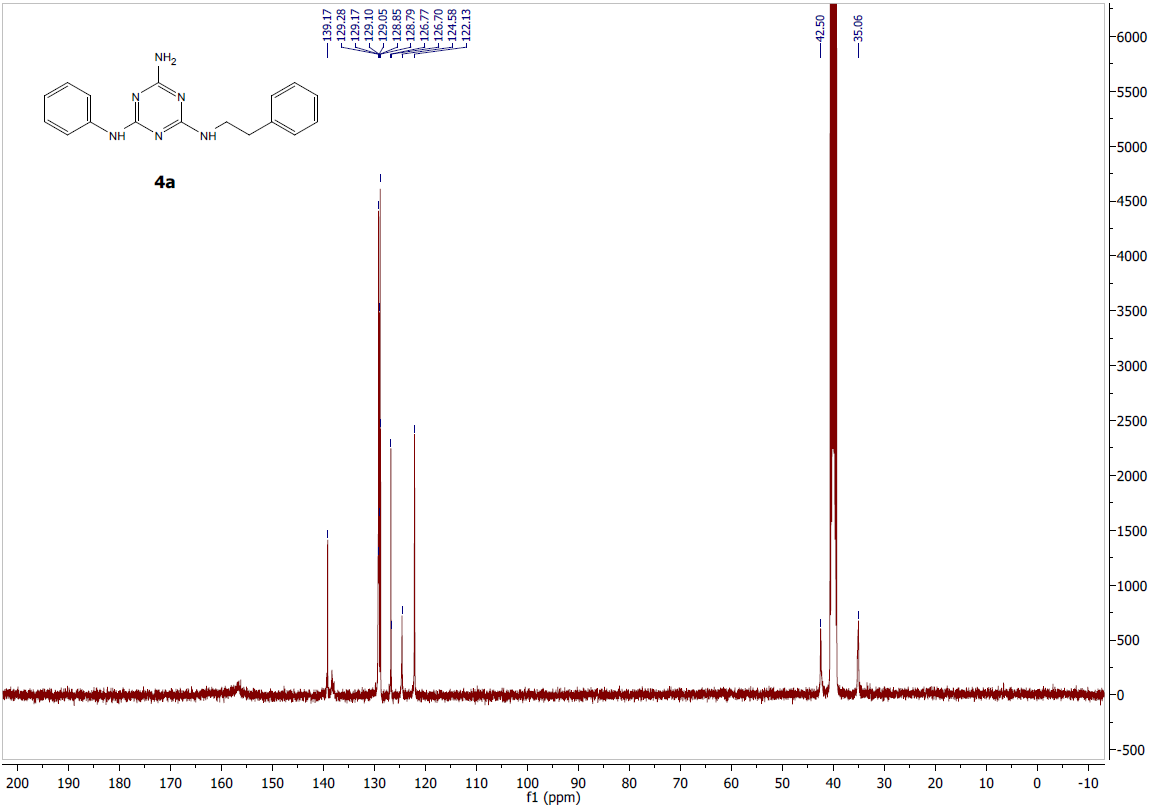


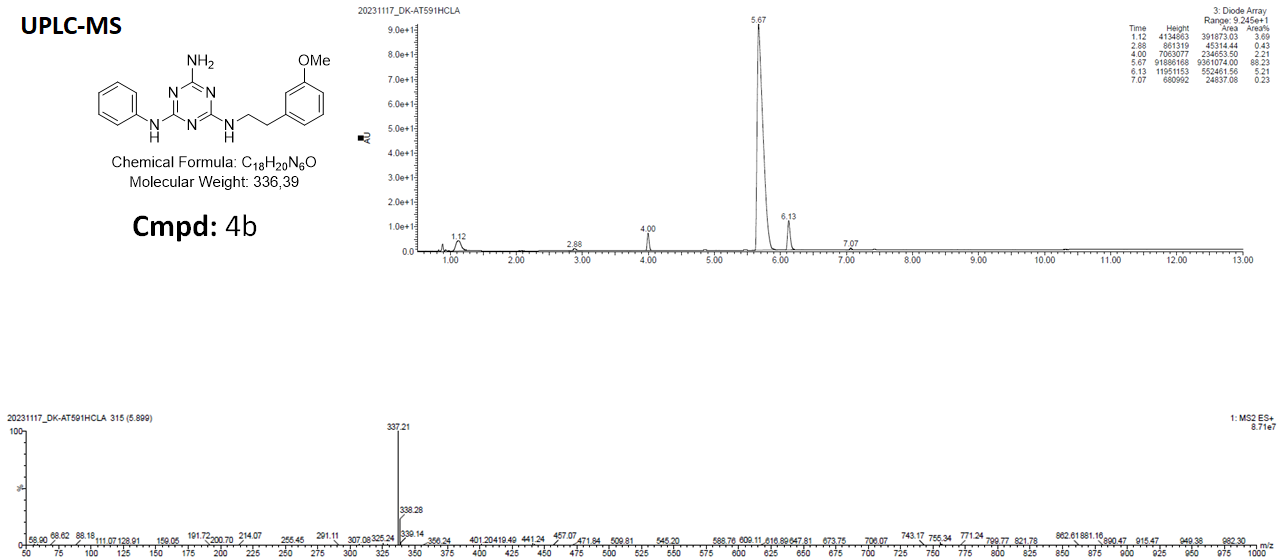


LC-MS according to method B


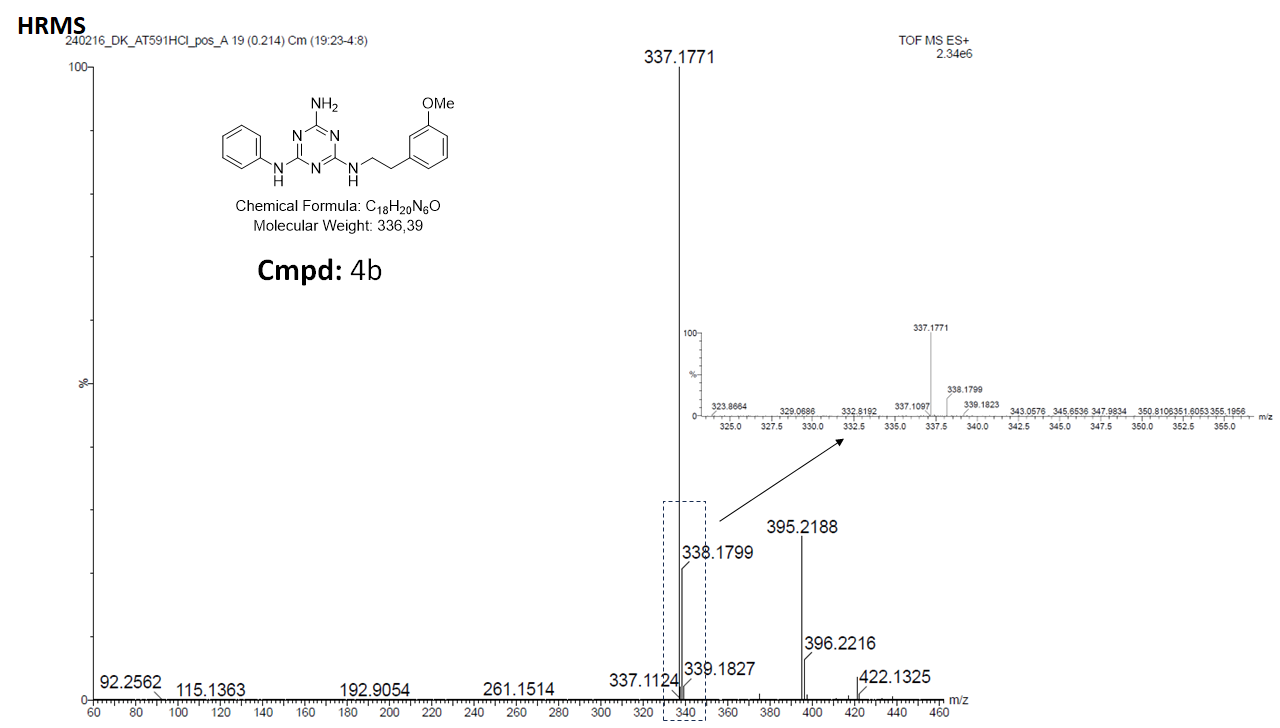


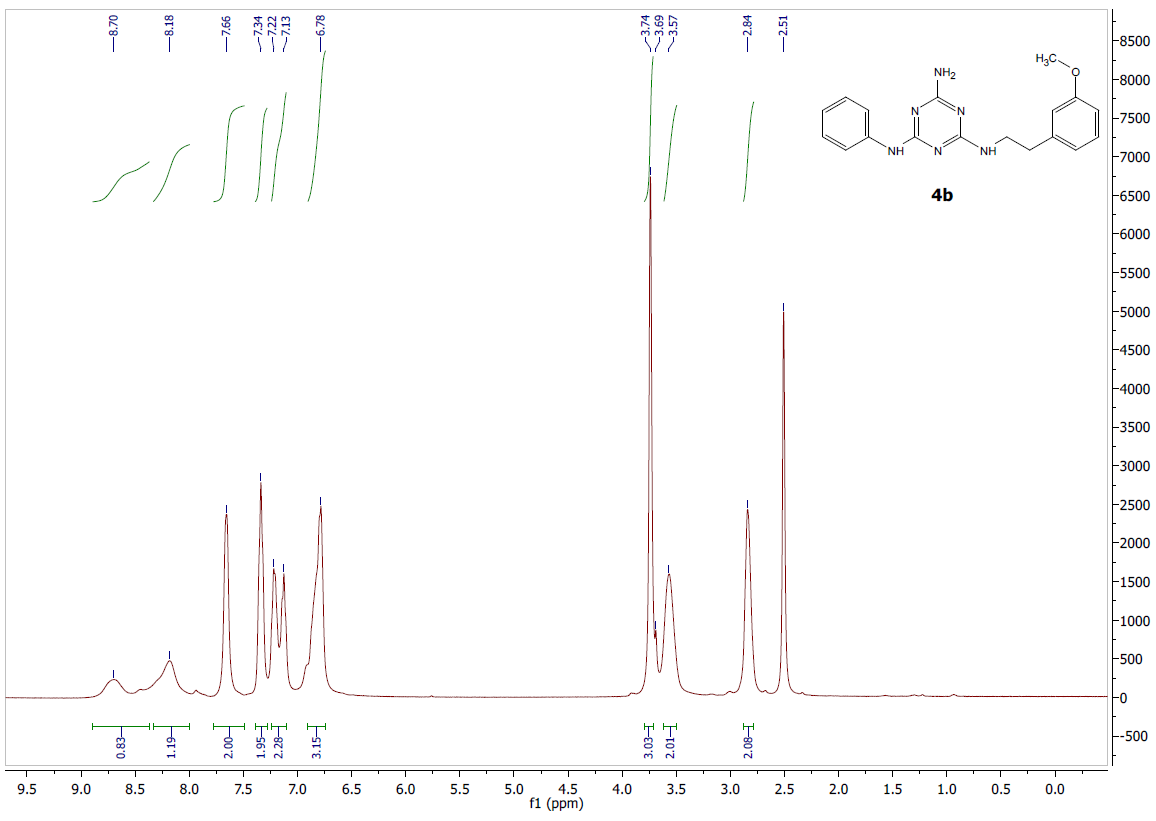


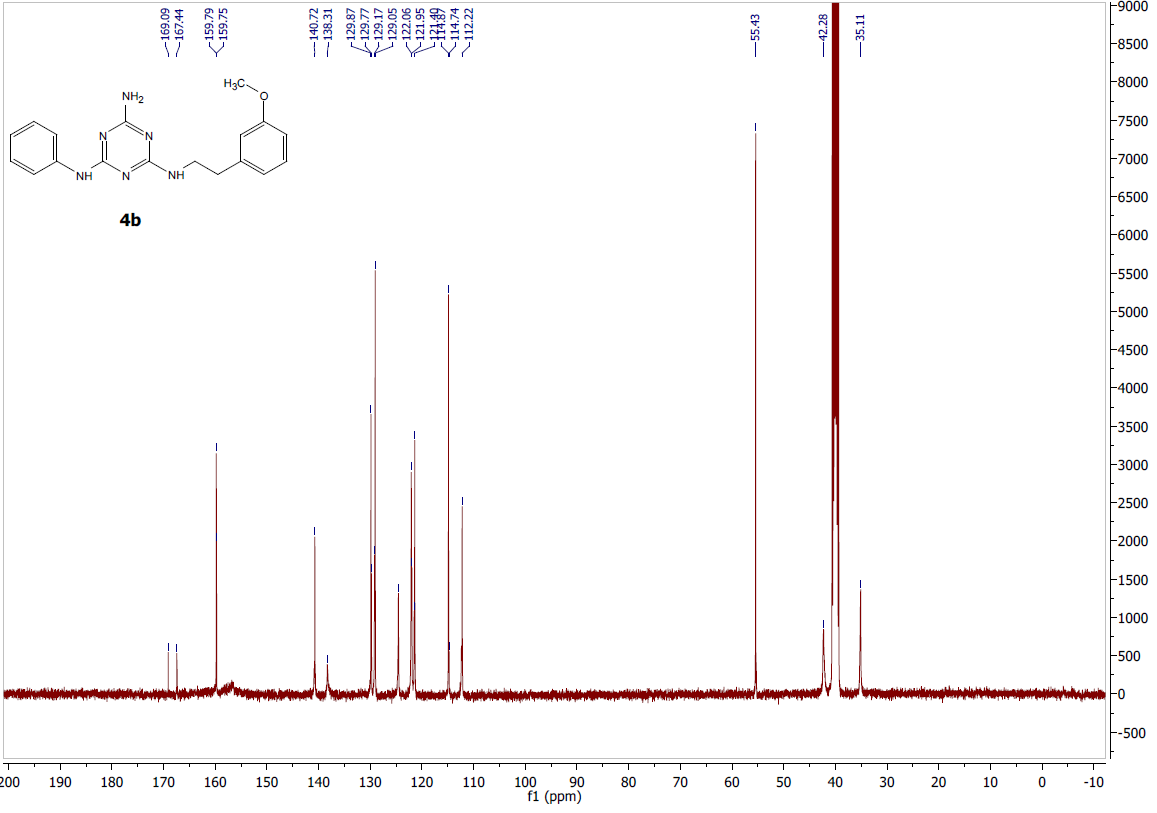


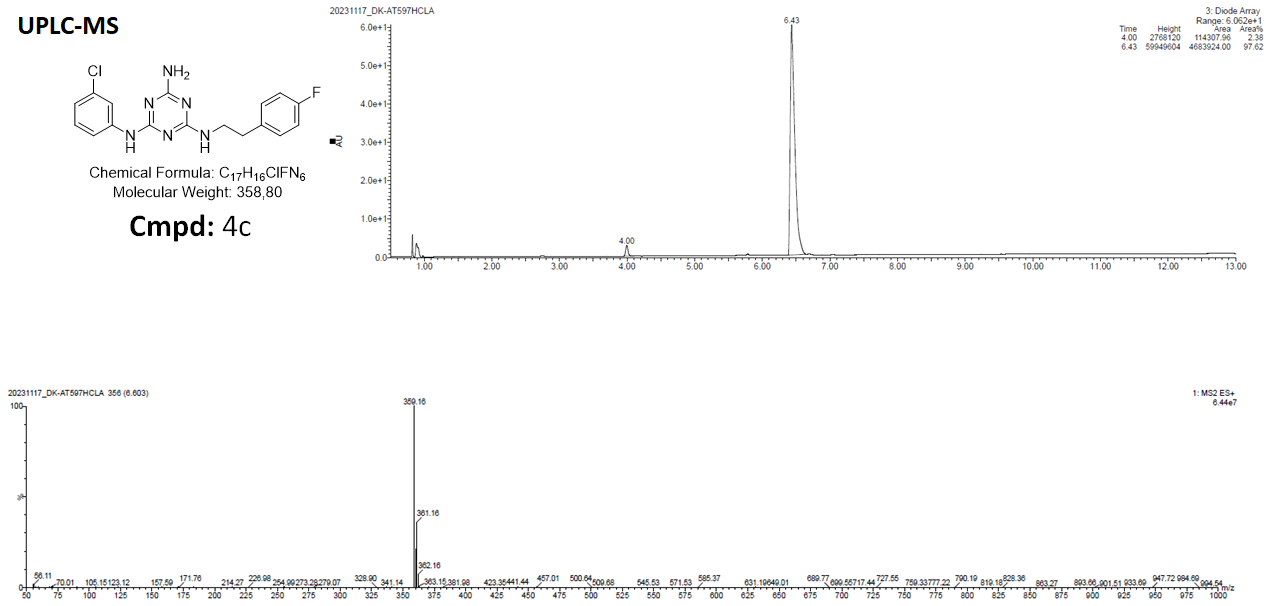


LC-MS according to method B


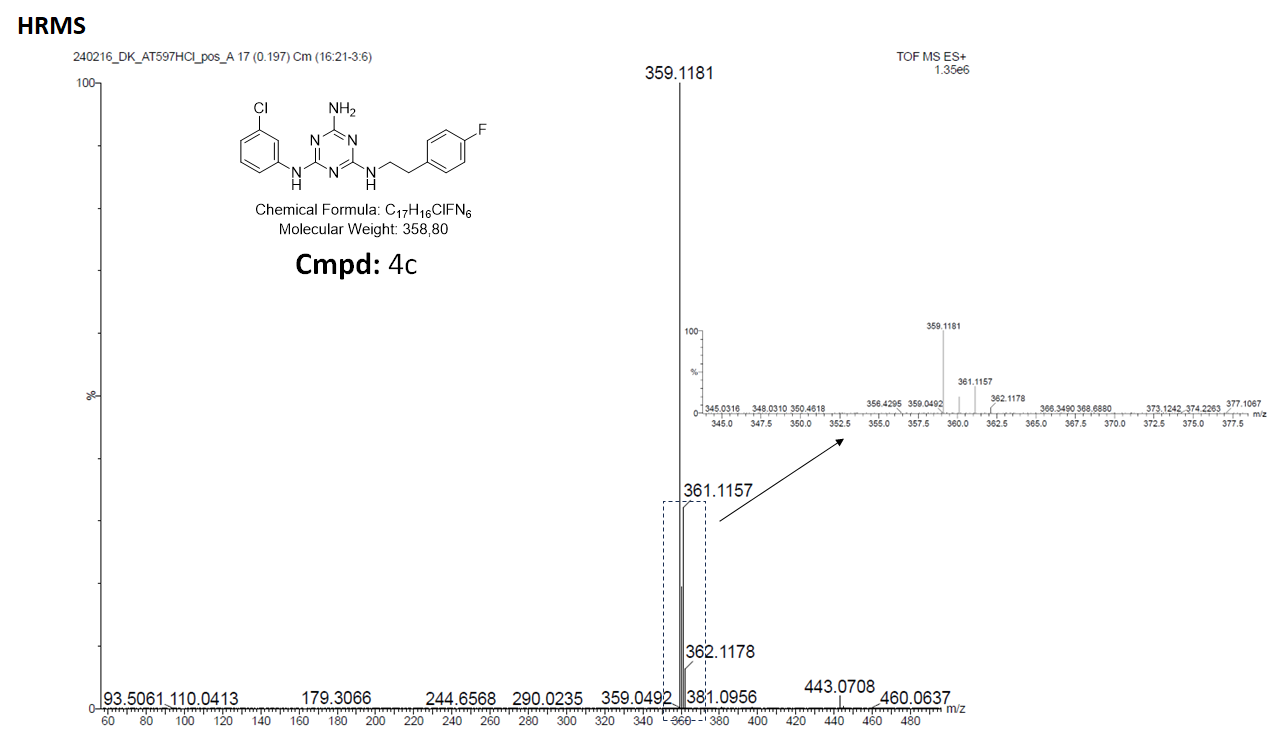


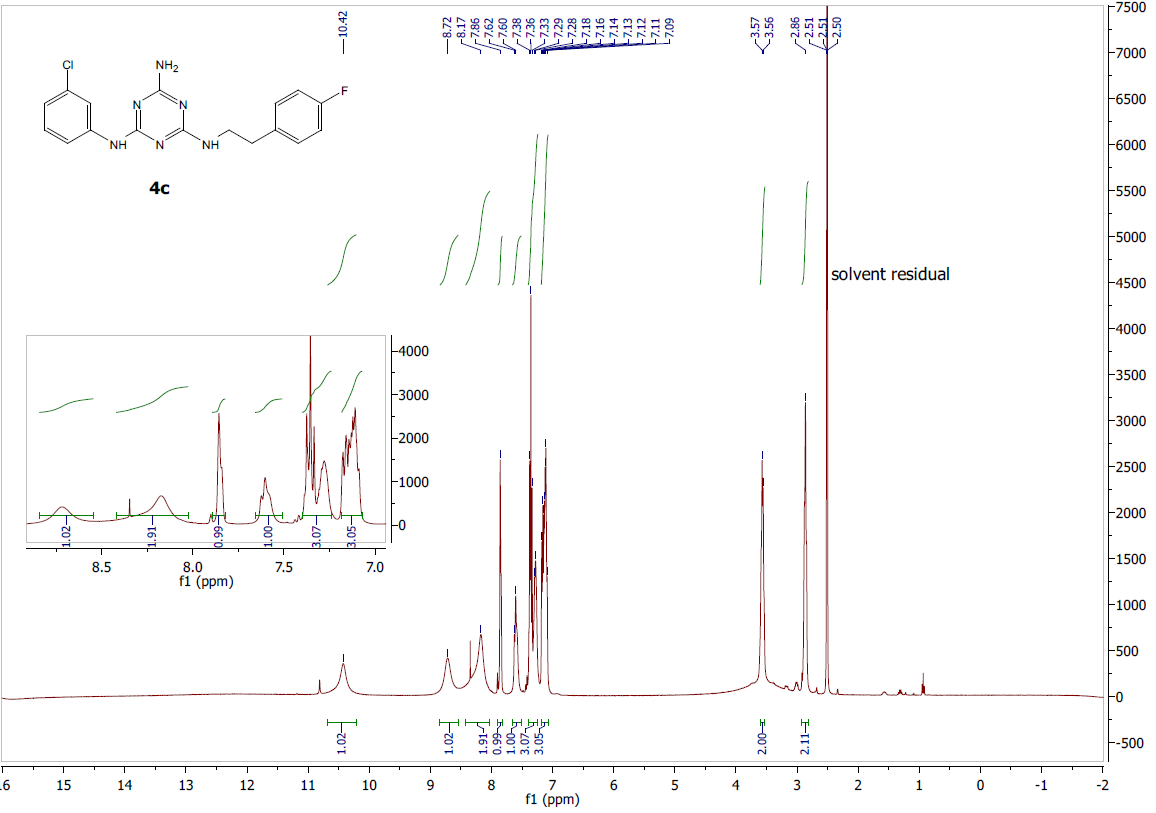


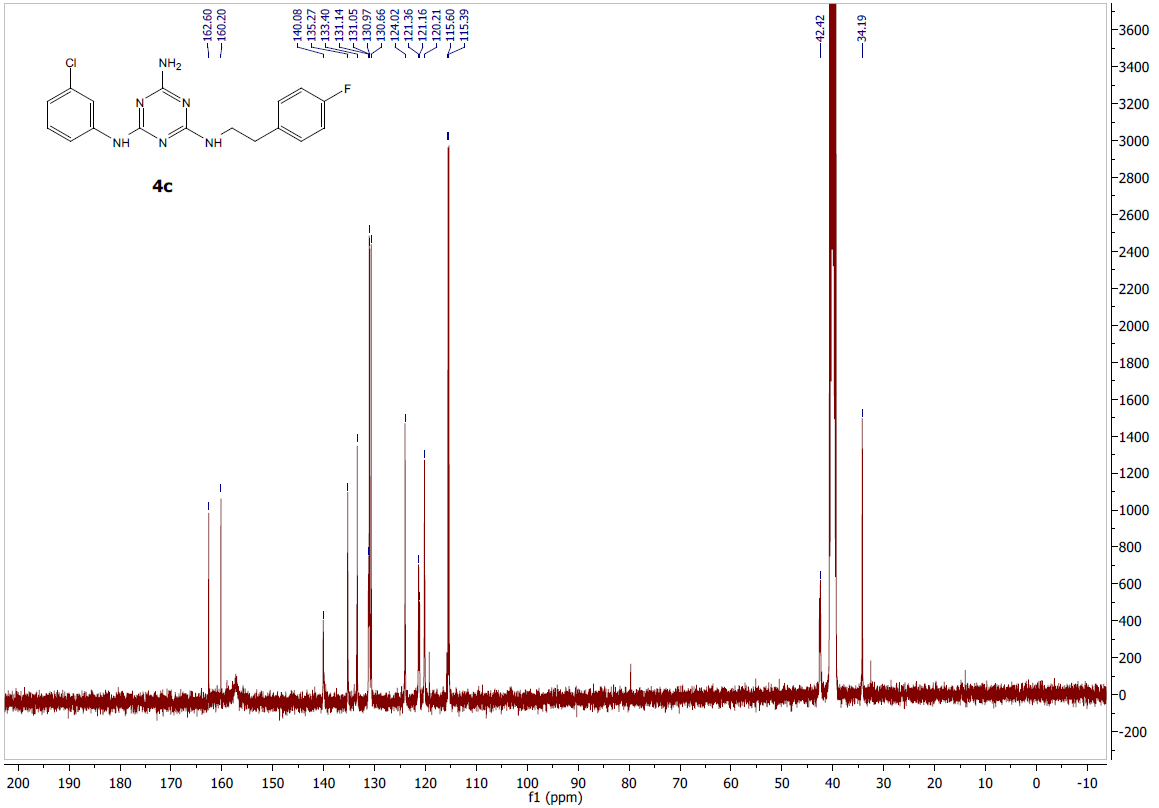


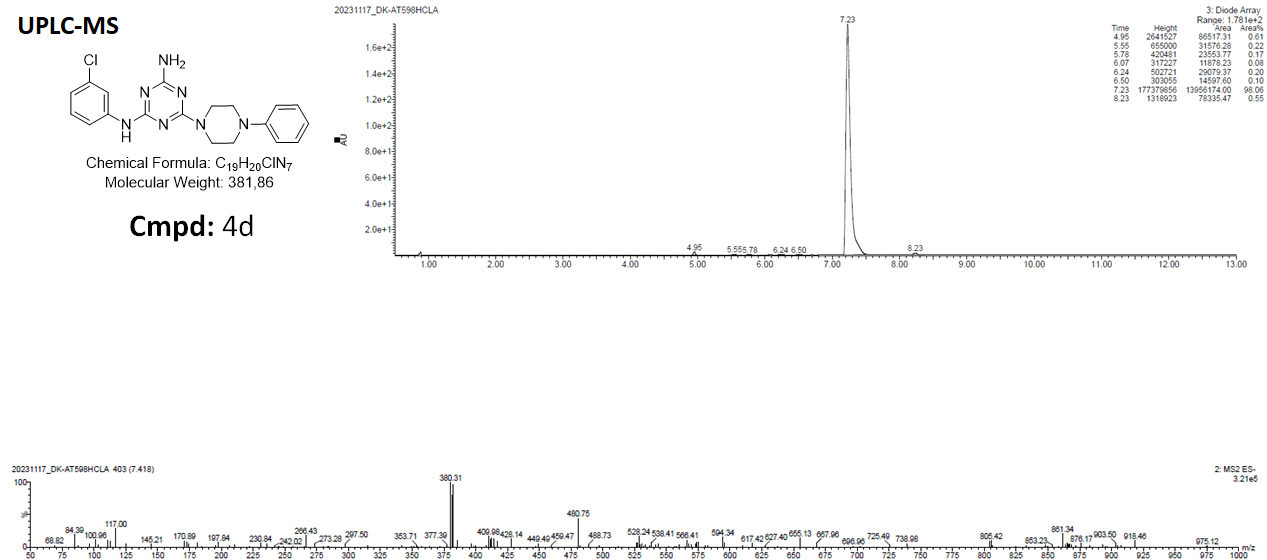


LC-MS according to method B


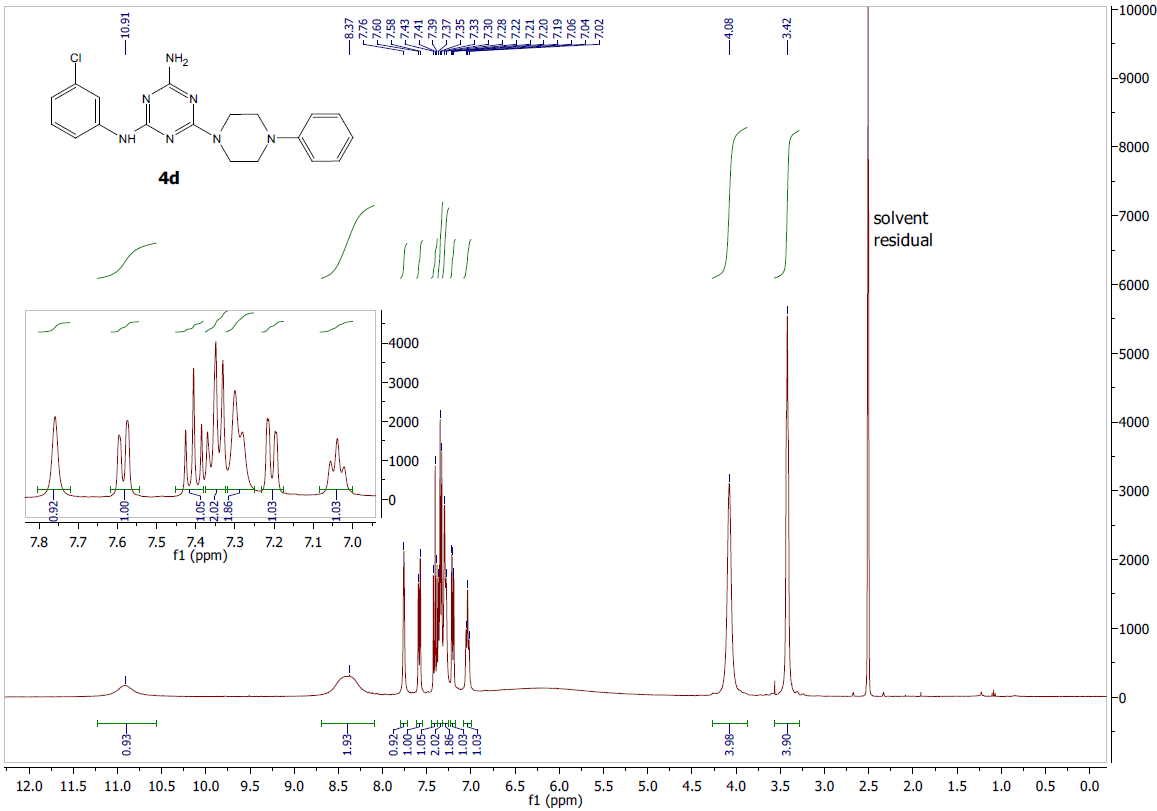


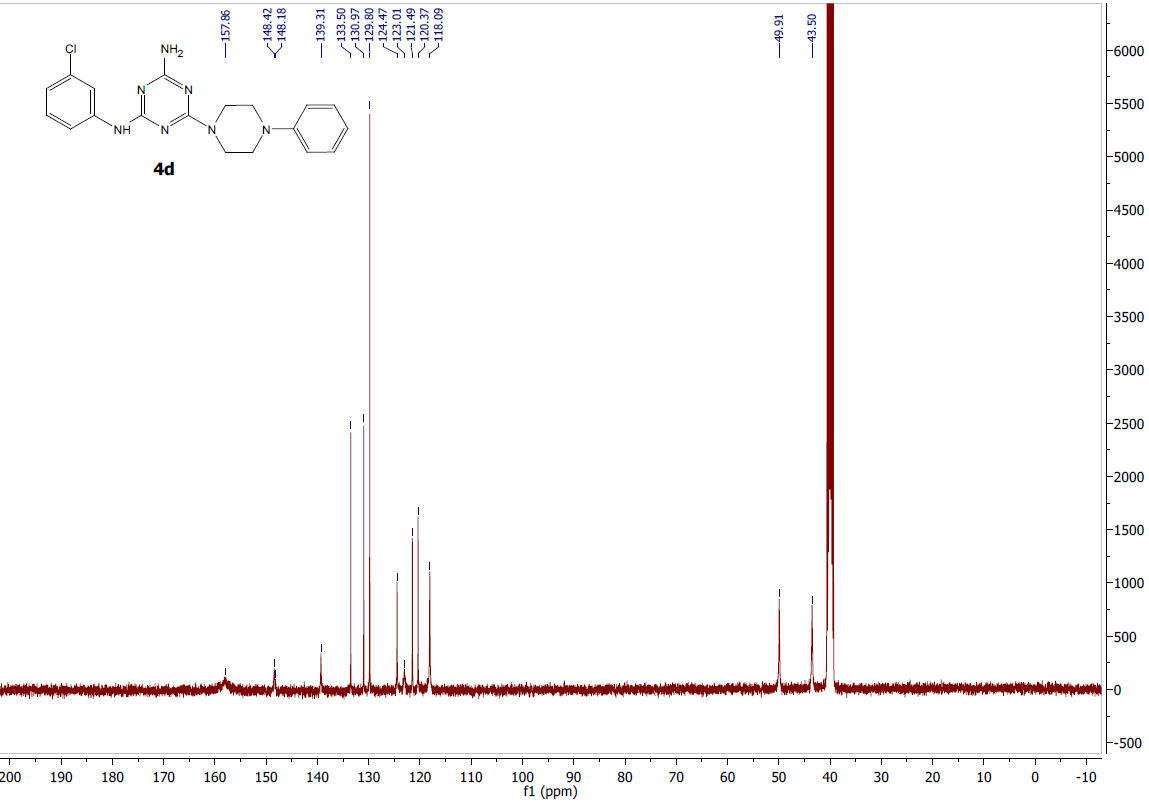


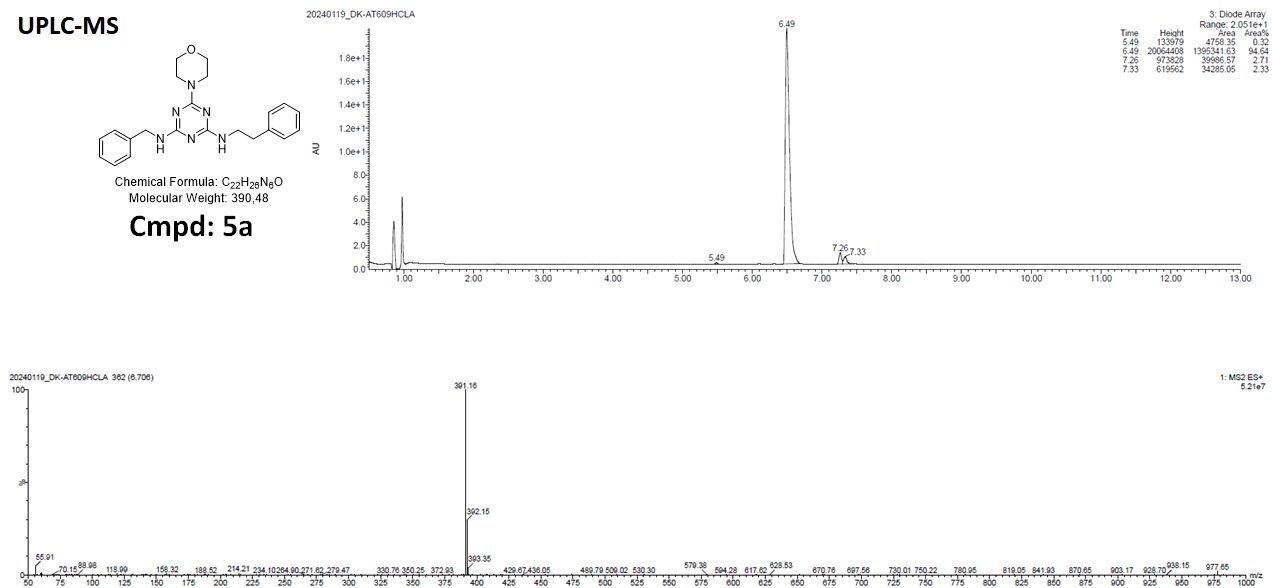


LC-MS according to method B


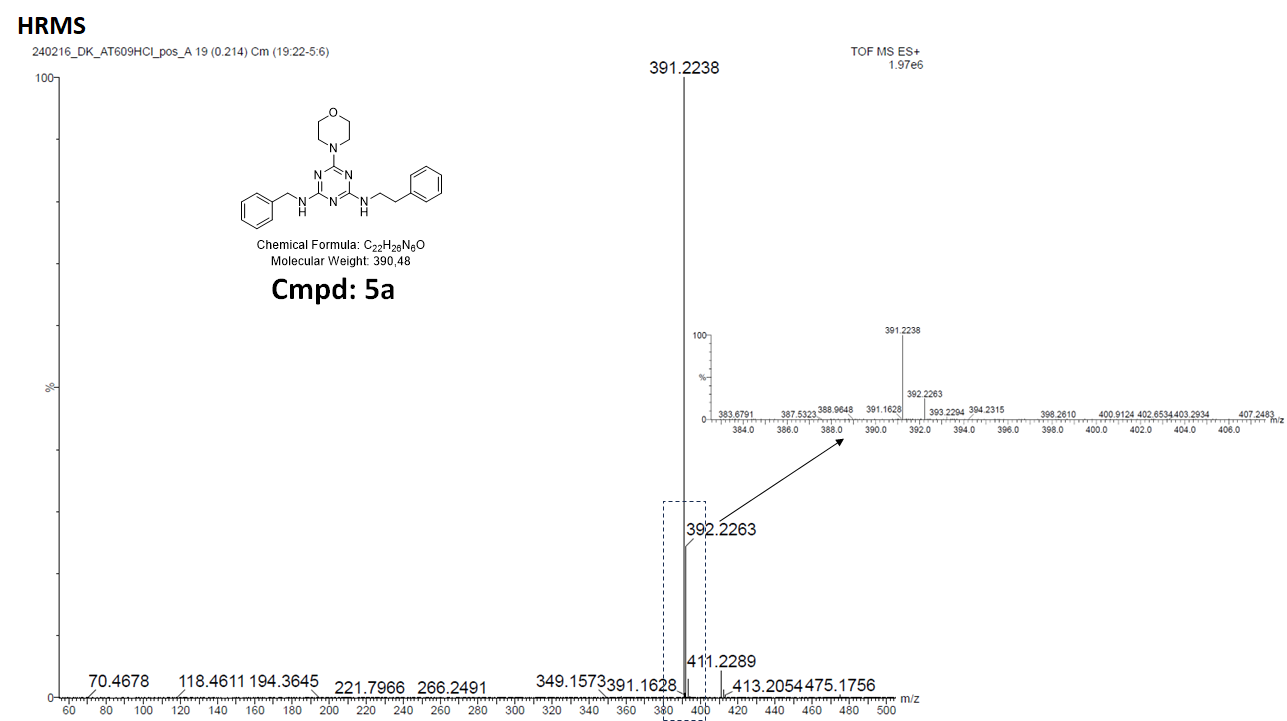


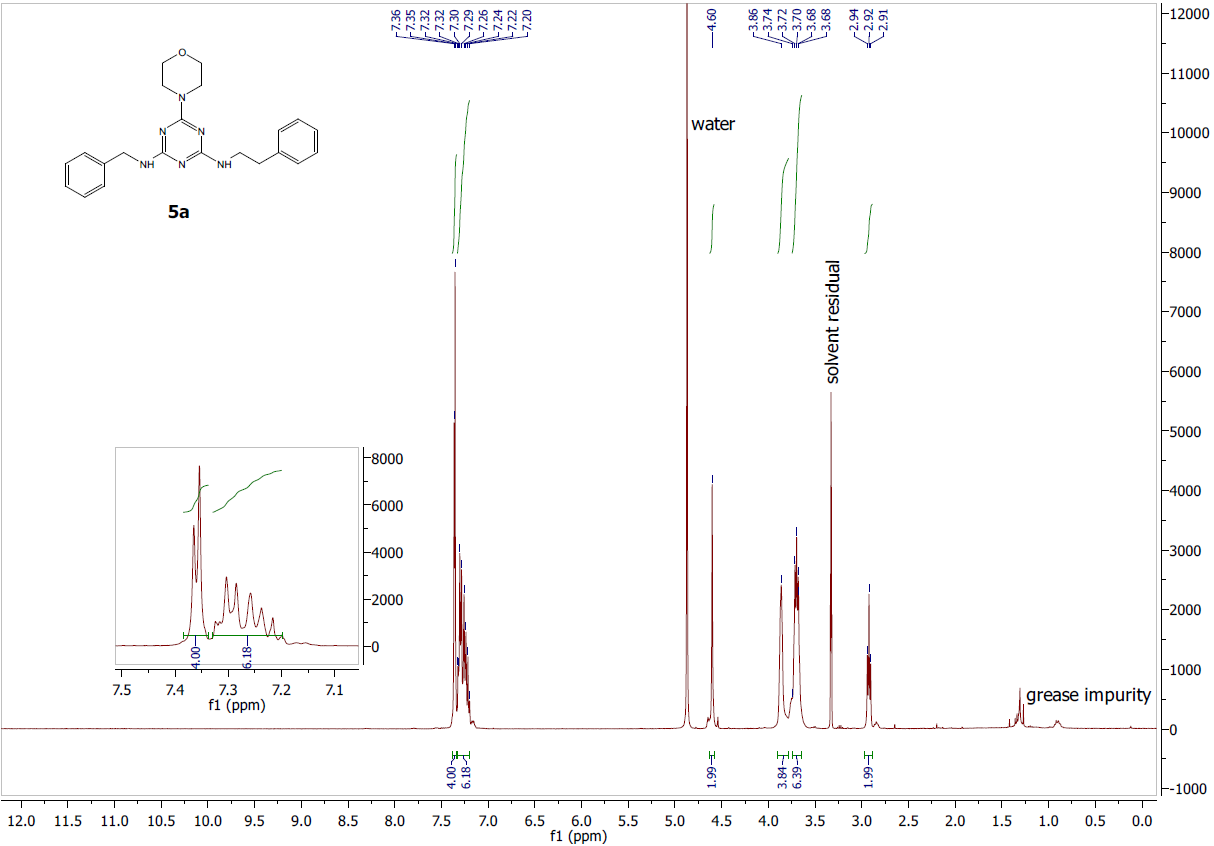


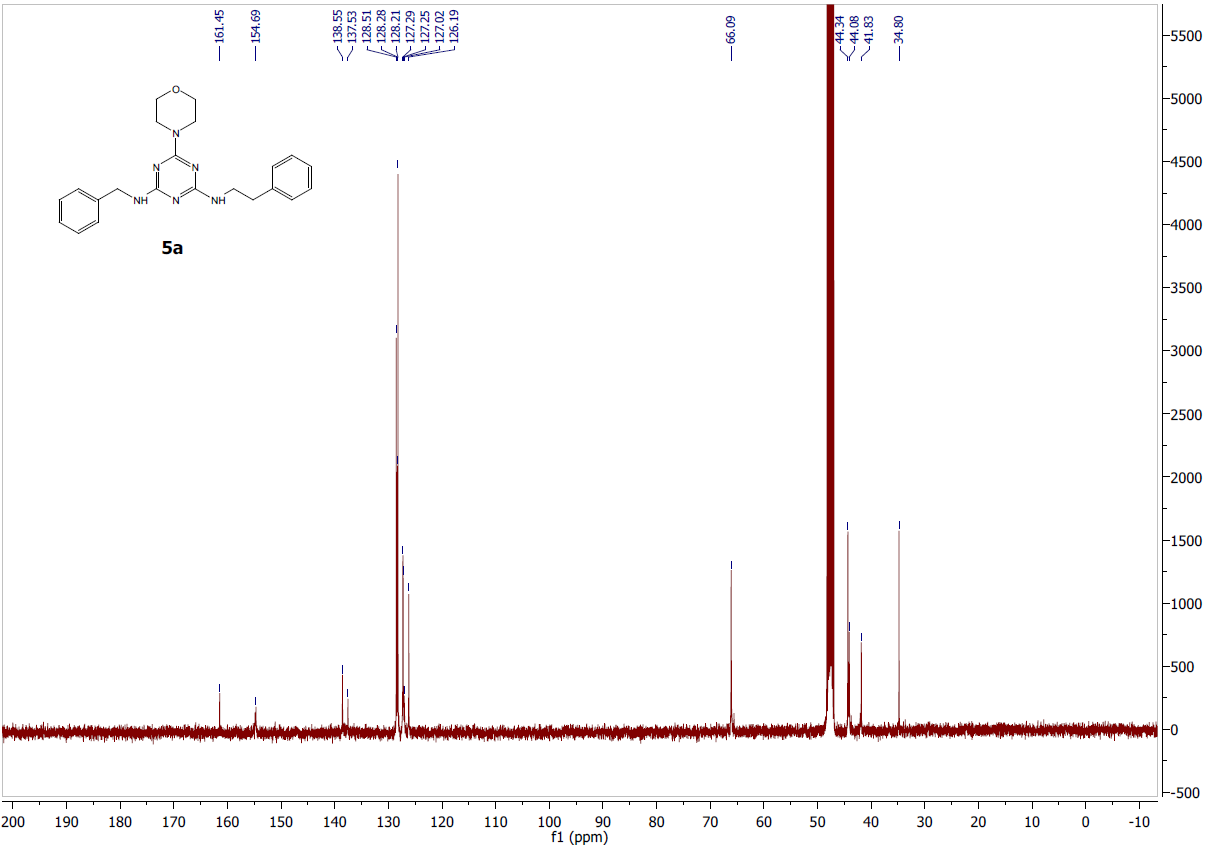


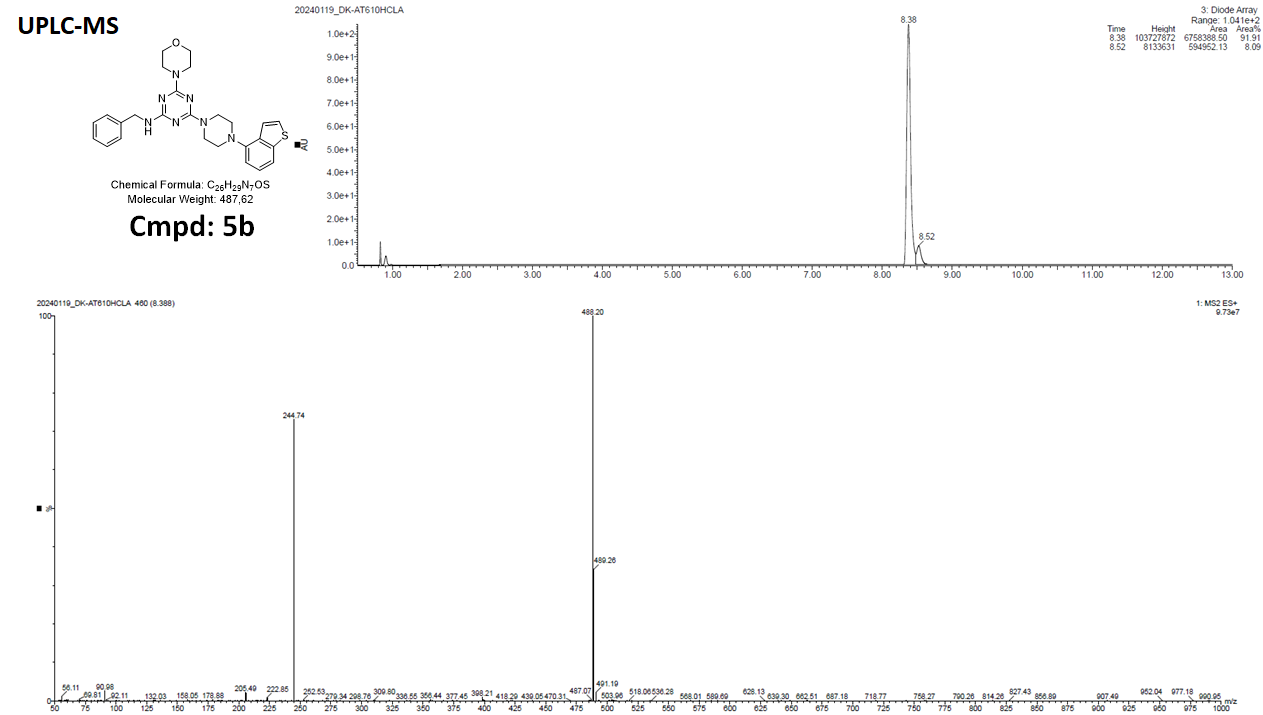


LC-MS according to method B


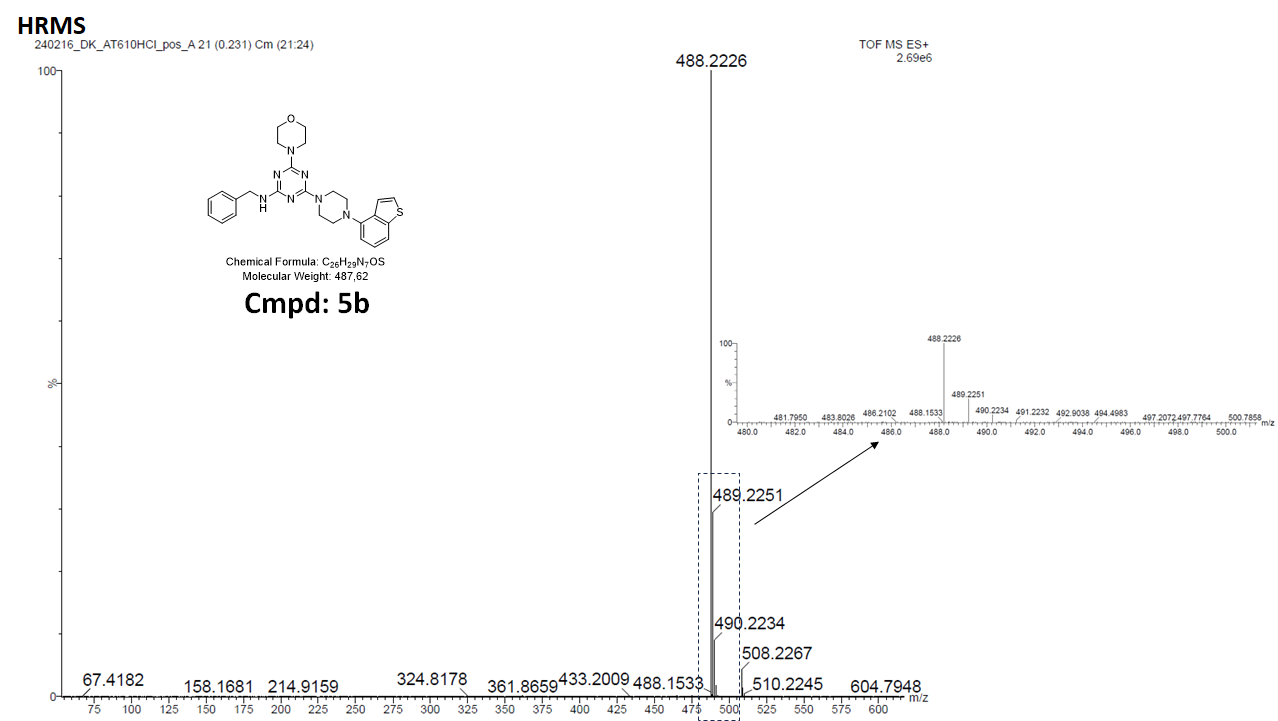


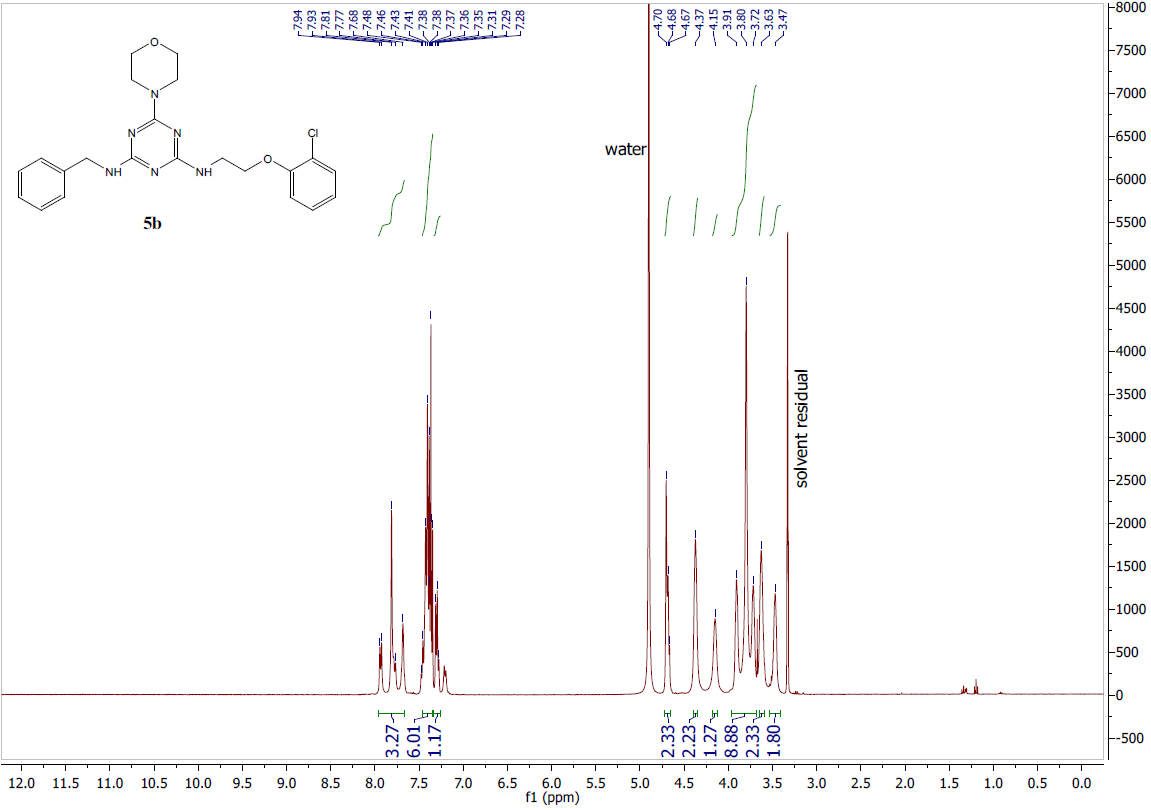


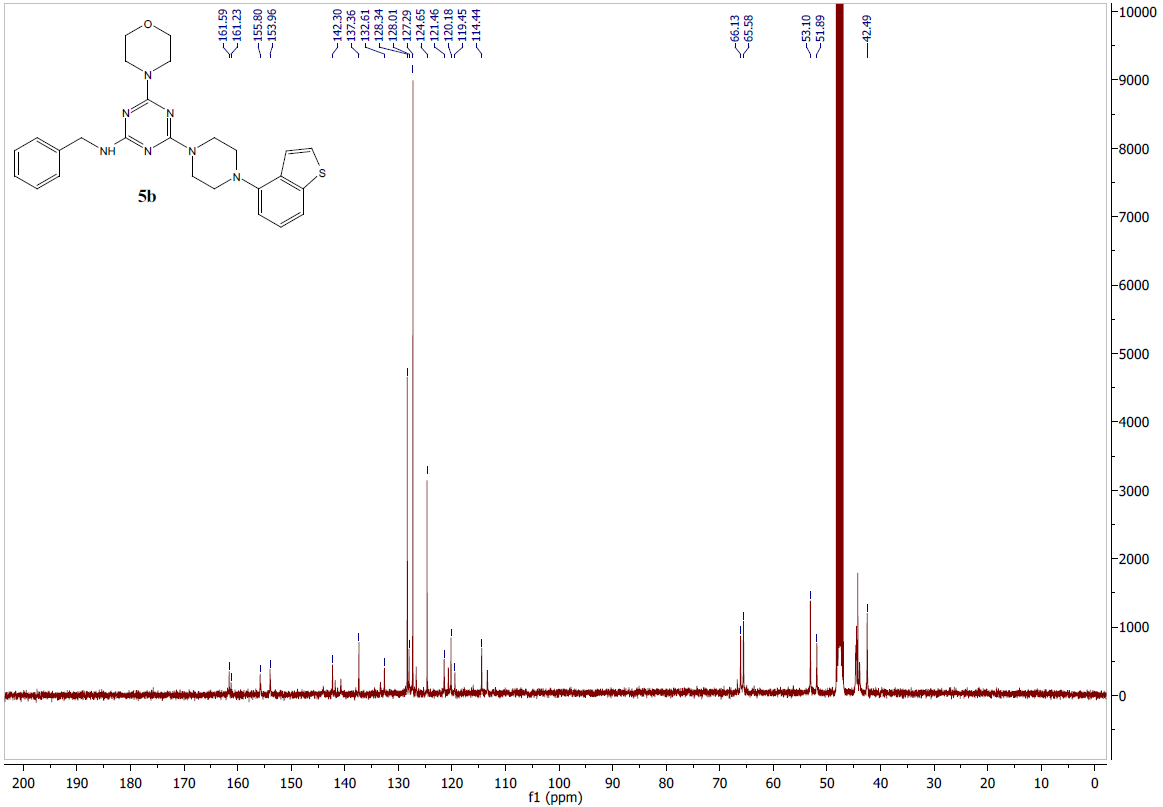


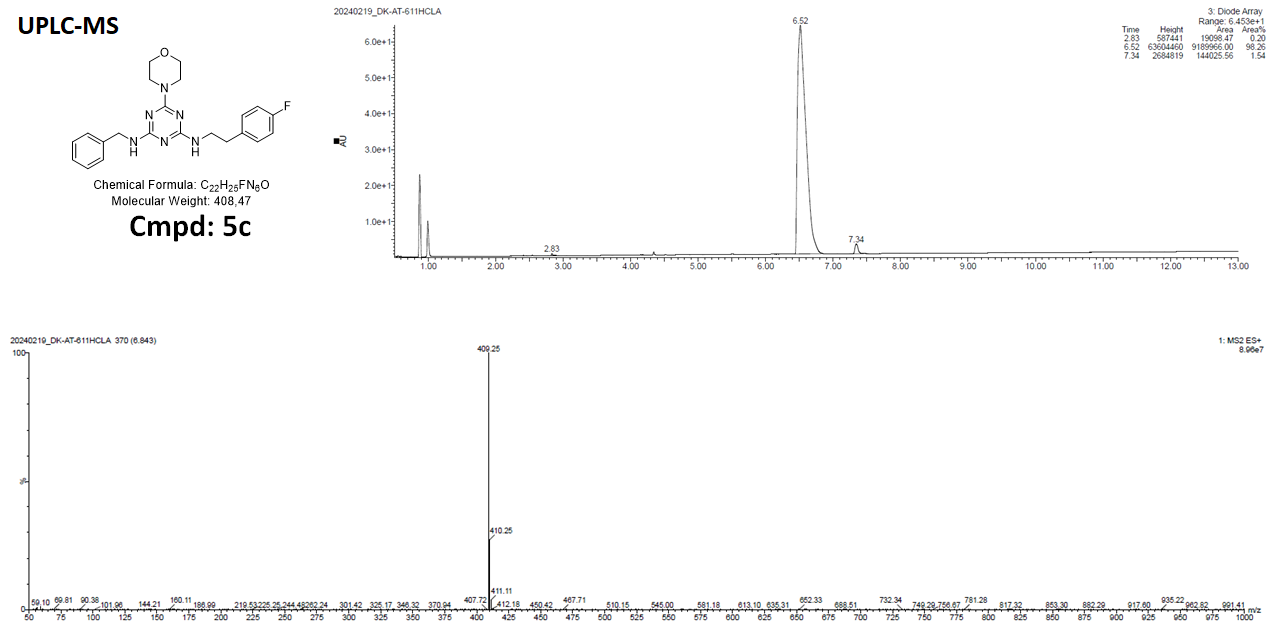


LC-MS according to method B


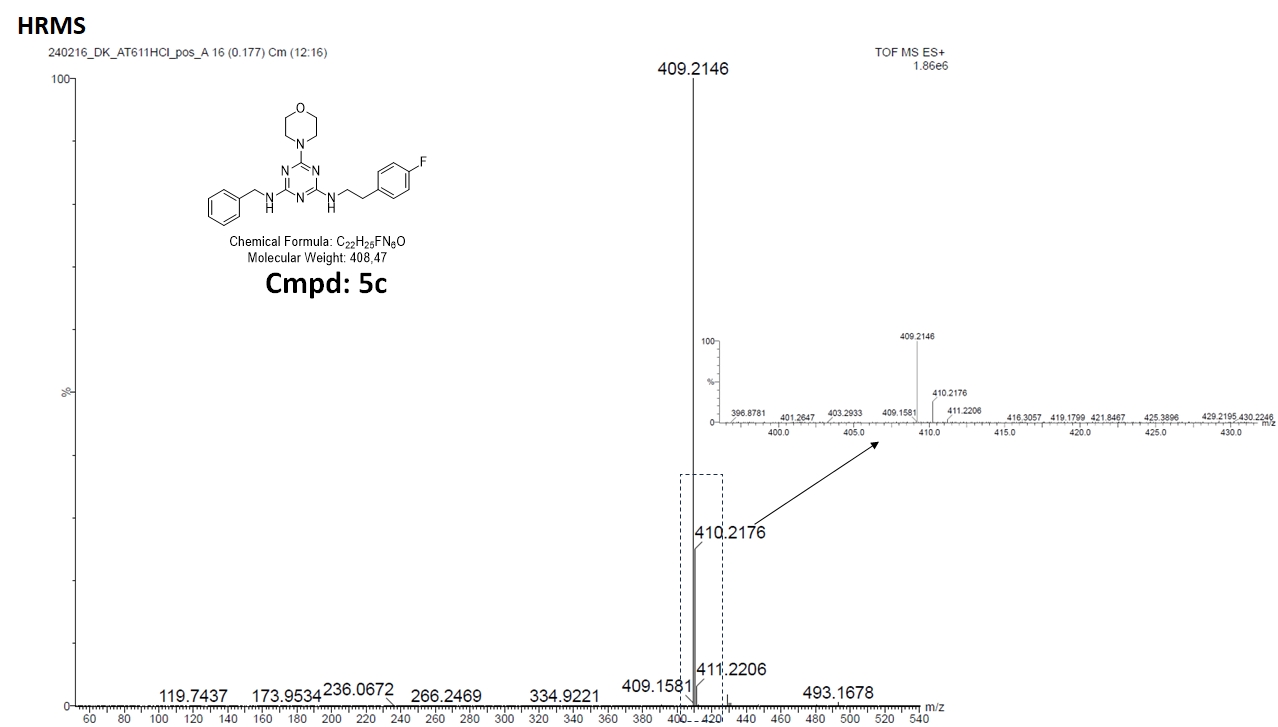


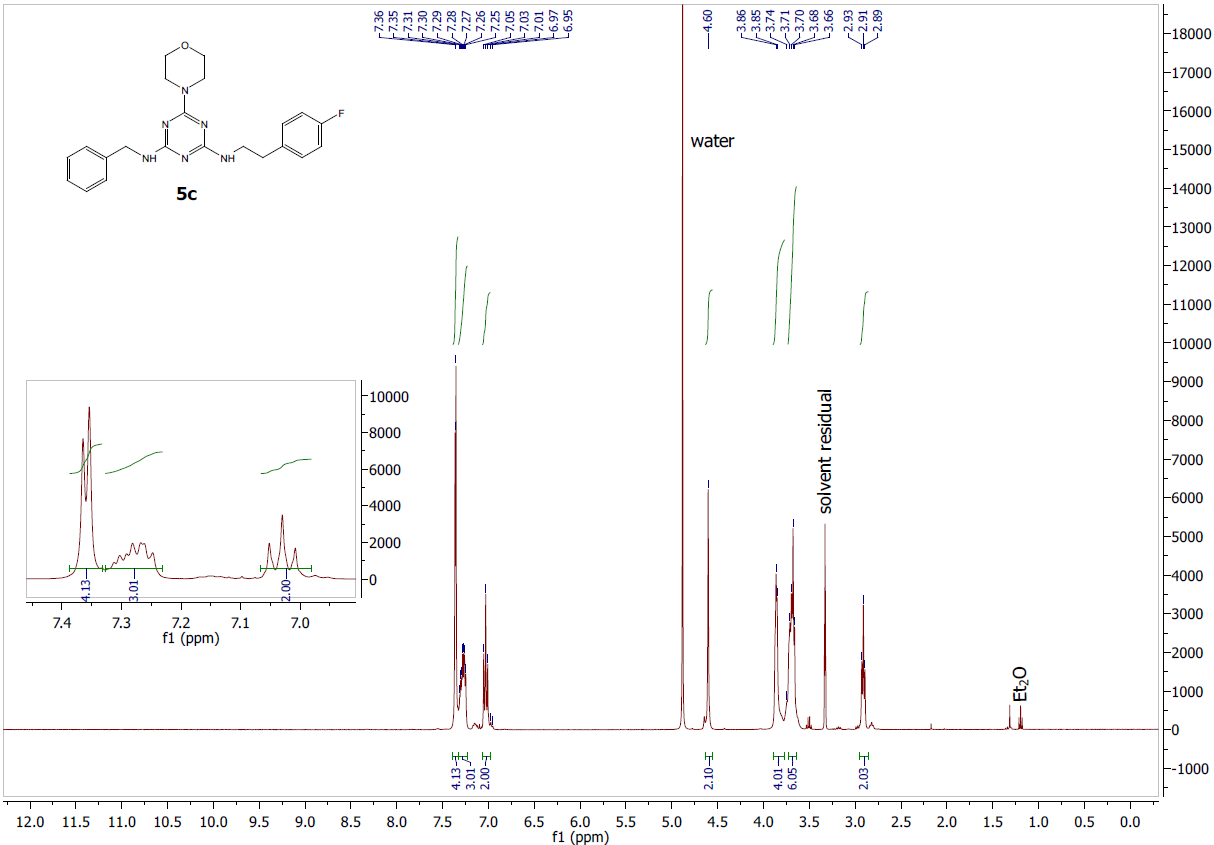


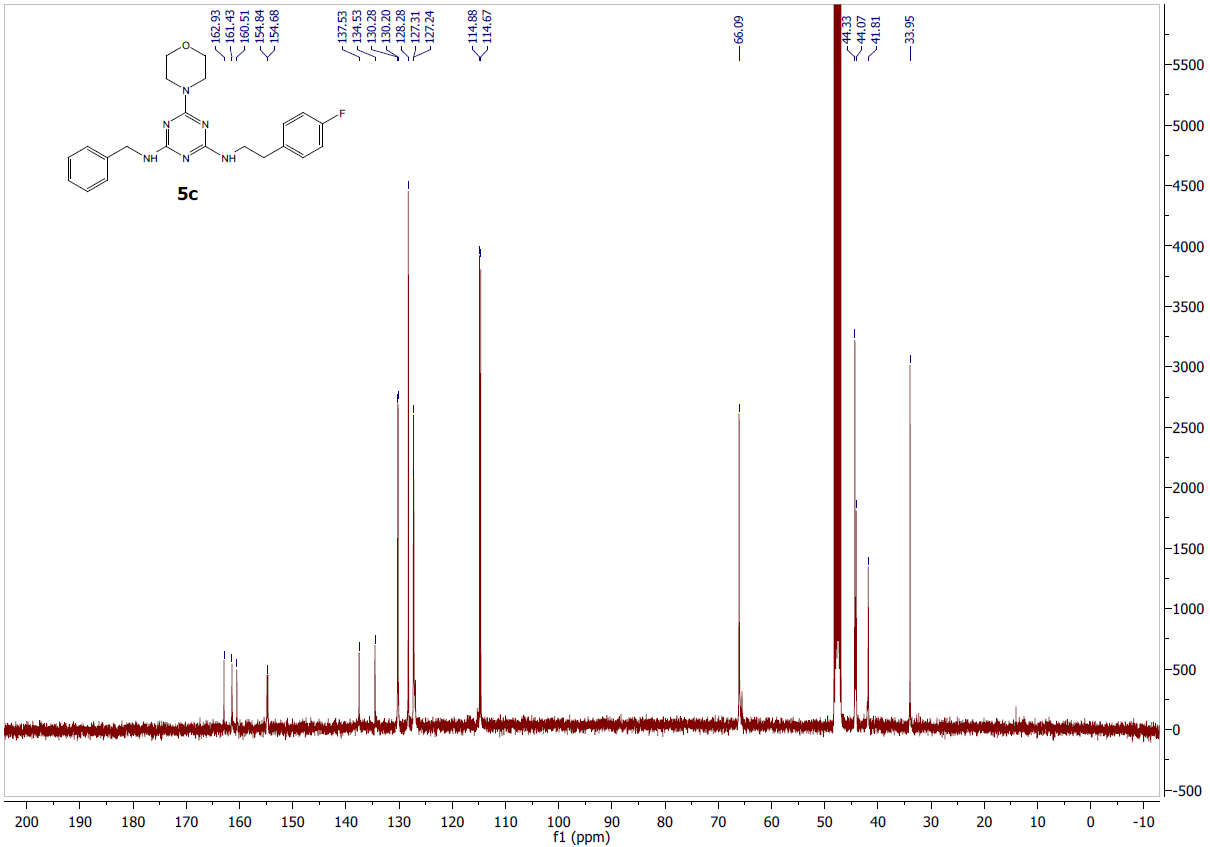


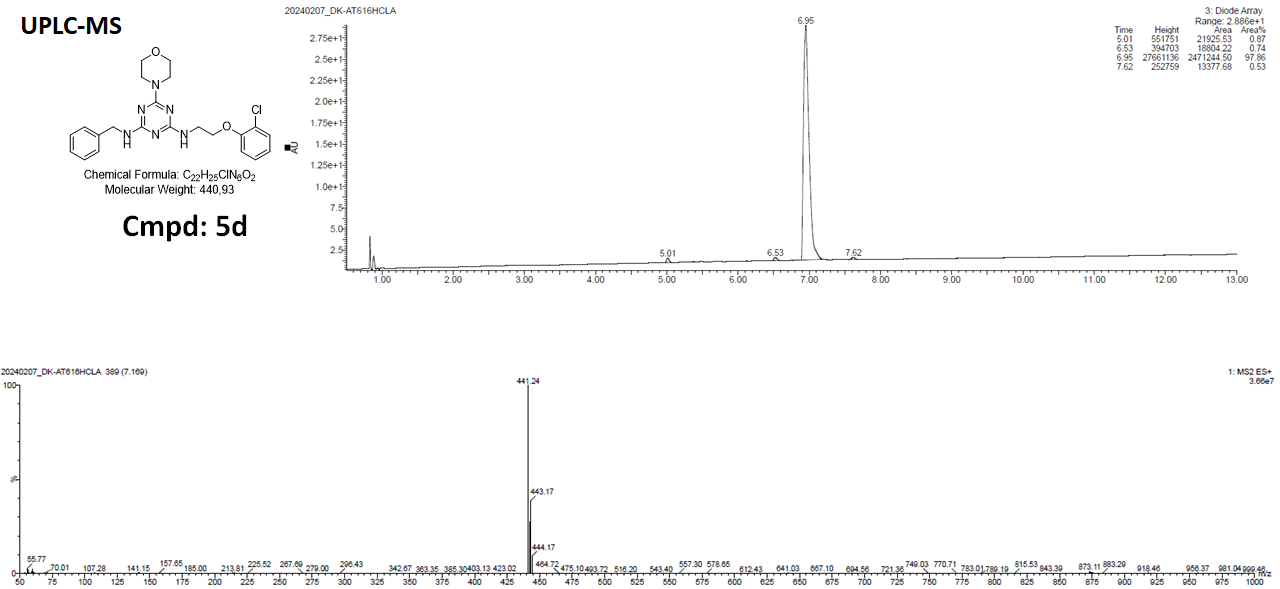


LC-MS according to method B


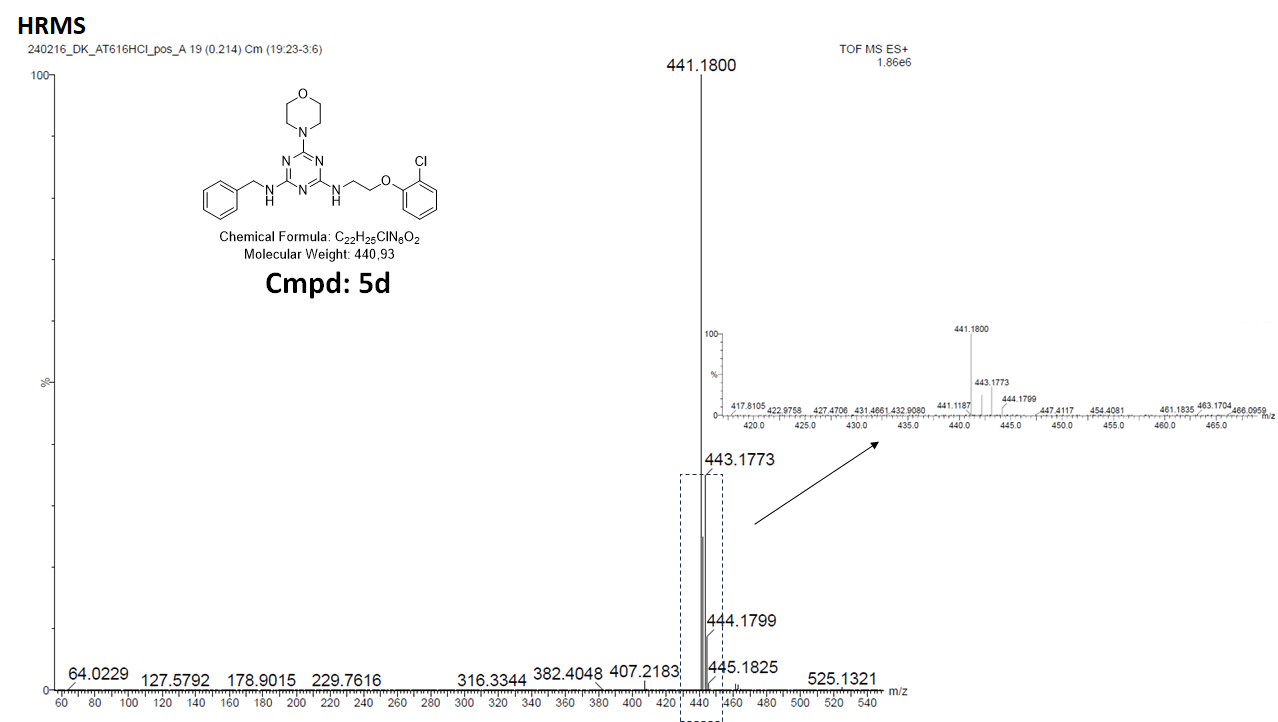


**Lipophilicity, drug-plasma protein binding, and phospholipids affinity assays**

All measurements were carried out using protocols proposed by Valko et al. [2,3] and adopted in our laboratory [4–6]. Briefly, these methods determined the Chromatographic Hydrophobicity Index (CHI) and % plasma protein binding (%PPB) using one gradient elution experiment and comparison to reference substances.

Each chromatographic experiment was carried out using the Prominence-1 LC-2030C 3D HPLC system (Shimadzu, Japan) controlled by the LabSolution system (version 5.90 Shimadzu, Japan). During the study, three chromatographic columns with varying chemical modifications of stationary phases were applied:

- IAM.PC.DD2 (10 × 4.6 mm x 10.0 µm with a guard column; Regis Technologies; USA);
- C_18_ Hypersil GOLD^TM^ (50 mm × 4.6 mm; 5.0 µm with a guard column; Thermo Scientific, USA);
- Chiralpak^®^ HSA (100 × 4 mm; 5 µm with a safety guard column; Daicel Chiral Technologies, USA)

The reference substances for the calibration of HSA, C_18,_ and IAM columns were purchased: acetanilide, butyrophenone, diclofenac and octanophenone (Alfa Aesar, Haverhill, USA); acetophenone, benzimidazole, colchicine, indole, indomethacin, paracetamol and theophylline (Sigma-Aldrich, Steinheim, Germany); nicardipine and nizatidine (Cayman Chemical, Michigan, USA); carbamazepine, heptanophenone, hexanophenone, propiophenone and valerophenone (Acros Organic, Pittsburg, USA).

For all measurements, mobile phase A was aqueous 50 mM ammonium acetate (VWR International, Leuven, Belgium) adjusted to pH 7.4 with concentrated ammonia solution (Avantor Performance Materials Poland S.A., Gliwice, Poland). Ultrapure water with a resistivity of 18.2 MΩ was obtained from a Milli-Q water purification system (Merck Millipore, Darmstadt, Germany).

For measurements of phospholipid binding, mobile phase B was HPLC-grade acetonitrile (Chempur, Piekary Śląskie, Poland) with a linear gradient of 0 to 85% B for 5.25 min and then held at 85% ACN for 0.5 minutes. The mobile phase flow rate was 1.5 mL/min and the IAM.PC.DD2 column was maintained at 30°C.

For lipophilicity measurements, solvents and the flow rate were the same as for IAM chromatography. The C_18_ Hypersil GOLD^TM^ column was maintained at 40°C. Similarly, a linear gradient was applied from 0 to 5.25 min, but in this case, 2 to 98% ACN was used and held at the maximum phase B concentration for 1.75 minutes.

For the determination of %PPB binding, HPLC grade isopropanol (VWR International, Leuven, Belgium) was used as mobile phase B. For the first 15 minutes, a linear gradient from 0 to 20% isopropanol was applied and then held at 20% isopropanol for 12 minutes. In the last 5 minutes of the sequence, the mobile phase mixture was again pure ammonium acetate solution. The column was held at 30°C, whereas the flow rate was 0.9 mL/min.

Before chromatographic experiments, solutes were dissolved in dimethyl sulfoxide (Avantor Performance Materials Poland S.A., Poland) to obtain 200 µg/mL concentration. Detection was performed in the UV region at a wavelength of 190 nm to 300 nm. The injected volume was 5 μL, and each compound was analyzed in triplicate. Retention times for the studied molecules are listed in supplementary materials (SI2).

**References**

[1] D. Kułaga, A.K. Drabczyk, G. Satała, G. Latacz, A. Boguszewska-Czubara, D. Plażuk, J. Jaśkowska, Int J Mol Sci., 2022, 23 (21), 13308

[2] K.L. Valko, T. Zhang, ADMET DMPK 2021, 9 (2), 151–165

[3] K.L. Valkó, J. Pharmaceut. Biomed. 2016, 130, 35–54

[4] D. Szulczyk, M. Woziński, M. Koliński, S. Kmiecik, A. Głogowska, E. Augustynowicz-Kopeć, M.A. Dobrowolski, P. Roszkowski, M. Struga, K. Ciura, Sci. Rep. 2023, 13, 16328

[5] S. Ulenberg, K. Ciura, P. Georgiev, M. Pastewska, G. Ślifirski, M. Król, F. Herold, T. Bączek, Microchem. J., 2022, 175, 107183

[6] K. Ciura, S. Kovačević, M. Pastewska, H. Kapica, M. Kornela, W. Sawicki, J. Chromatogr. A 2021, 1660, 462666
