# Supplementary material for: Green synthesis of 1,3,5-triazine derivatives using a sonochemical protocol
Source: Ultrason Sonochem. 2024 Jun 12;108:106951. doi: 10.1016/j.ultsonch.2024.106951 (PMC11227021; doi:10.1016/j.ultsonch.2024.106951)
Supplement: Supplementary Data 2 [file mmc2.pdf]

	IAM						C18 pH 2.6						C18 pH 7.4						C18 pH 10.4						HSA							
	tr1	tr2	tr3	tmean	SD	CHI IAM	tr1	tr2	tr3	tmean	SD	CHI pH 2.6	tr1	tr2	tr3	tmean	SD	CHI pH 7.4	tr1	tr2	tr3	tmean	SD	CHI pH 10.4	tr1	tr2	tr3	tmean	logt	SD	logKHSA	%HSA
3	5,20	5,20	5,08	5,16	0,07	43,35	3,76	3,74	3,74	3,75	0,01	67,30	4,35	4,34	4,35	4,35	0,00	85,33	3,72	3,70	3,74	3,72	0,02	84,93	18,25	18,27	18,26	18,26	1,26	0,01	1,32	96,36
3a	5,13	5,15	4,97	5,08	0,10	42,14	3,71	3,69	3,68	3,69	0,01	65,39	4,28	4,27	4,26	4,27	0,01	83,04	3,68	3,64	3,66	3,66	0,02	83,07	18,20	17,93	18,05	18,06	1,26	0,13	1,31	96,25
3b	5,10	5,12	4,93	5,05	0,10	41,65	3,77	3,75	3,77	3,76	0,01	67,84	4,31	4,32	4,32	4,31	0,01	84,32	3,70	3,69	3,78	3,72	0,05	85,00	16,59	16,36	16,46	16,47	1,22	0,12	1,22	95,26
3c	5,50	5,50	5,49	5,50	0,00	48,51	4,16	4,09	4,08	4,11	0,04	80,31	4,65	4,64	4,64	4,64	0,01	94,36	4,04	3,99	4,03	4,02	0,03	94,14	17,62	17,56	17,60	17,59	1,25	0,03	1,28	95,99
3d	5,10	5,10	4,96	5,05	0,08	41,66	3,77	3,69	3,67	3,71	0,06	65,88	4,25	4,25	4,25	4,25	0,00	82,34	3,63	3,61	3,63	3,62	0,02	81,98	16,02	15,96	16,00	15,99	1,20	0,03	1,19	94,91
4a	5,13	5,16	4,99	5,09	0,09	42,25	3,99	4,01	4,03	4,01	0,02	76,73	4,36	4,36	4,35	4,36	0,00	85,66	3,80	3,73	3,88	3,80	0,08	87,41	13,05	12,98	13,03	13,02	1,11	0,04	1,00	91,82
4b	5,04	5,04	4,85	4,98	0,11	40,50	4,06	4,04	4,05	4,05	0,01	78,12	4,30	4,32	4,31	4,31	0,01	84,33	3,70	3,69	3,71	3,70	0,01	84,27	12,27	12,29	12,32	12,29	1,09	0,02	0,95	90,74
4c	5,69	5,68	5,58	5,65	0,07	50,88	4,57	4,57	4,57	4,57	0,00	96,90	4,73	4,74	4,74	4,74	0,01	97,13	4,08	4,11	4,07	4,09	0,02	96,10	13,50	13,52	13,59	13,53	1,13	0,05	1,04	92,50
4d	5,91	5,91	5,80	5,87	0,06	54,25	4,97	4,96	4,94	4,96	0,02	110,76	5,04	5,03	5,05	5,04	0,01	106,34	4,47	4,42	4,43	4,44	0,03	106,85	21,91	22,01	22,00	21,98	1,34	0,05	1,49	97,84
5a	5,17	5,18	5,06	5,13	0,07	42,94	4,36	4,36	4,37	4,37	0,00	89,53	4,96	4,96	4,96	4,96	0,01	103,85	4,28	4,29	4,33	4,30	0,03	102,70	15,40	15,22	15,36	15,32	1,19	0,09	1,15	94,37
5b	5,93	5,94	5,86	5,91	0,05	54,84	5,68	5,71	5,72	5,70	0,02	137,46	5,78	5,80	5,78	5,79	0,01	128,90	5,19	5,14	5,15	5,16	0,03	128,93	33,19	33,21	33,26	33,22	1,52	0,04	1,88	99,68
5c	5,20	5,20	5,02	5,14	0,10	42,96	4,48	4,48	4,48	4,48	0,00	93,62	4,98	4,97	4,99	4,98	0,01	104,54	4,30	4,30	4,34	4,31	0,02	103,07	16,31	16,26	16,36	16,31	1,21	0,05	1,21	95,15
5d	4,70	4,71	4,43	4,61	0,16	34,93	4,39	4,38	4,36	4,38	0,01	89,85	4,40	4,41	4,40	4,40	0,01	87,05	3,76	3,76	3,80	3,77	0,02	86,49	8,92	8,99	8,99	8,97	0,95	0,04	0,65	82,56
